# Development of indicators to assess quality and patient pathways in interdisciplinary care for patients with 14 ambulatory-care-sensitive conditions in Germany

**DOI:** 10.1186/s12913-022-08327-1

**Published:** 2022-08-09

**Authors:** Wiebke Schüttig, Ronja Flemming, Christiane Höhling Mosler, Verena Leve, Olaf Reddemann, Annemarie Schultz, Emmanuelle Brua, Matthias Brittner, Frank Meyer, Johannes Pollmanns, Johnannes Martin, Thomas Czihal, Dominik von Stillfried, Stefan Wilm, Leonie Sundmacher

**Affiliations:** 1 Chair of Health Economics, Technical University of Munich, Georg-Brauchle-Ring 60/62, 80992 Munich, Germany; 2 Department for Health Services Management, Ludwig-Maximilian-University Munich, Munich, Germany; 3 AOK Health Insurance Rhineland / Hamburg, Kasernenstraße 61, 40213 Duesseldorf, Germany; 4 University Hospital Düsseldorf, Office of Quality Management and Patient Safety, Moorenstr. 5, 40225 Duesseldorf, Germany; 5 Institute of General Practice (ifam), Centre for Health and Society (chs), Medical Faculty, Heinrich Heine University Düsseldorf, Moorenstr. 5, 40225 Düsseldorf, Germany; 6 Regional Association of Statutory Health Insurance Physicians Hamburg, Humboldtstraße 56, 22083 Hamburg, Germany; 7 Regional Association of Statutory Health Insurance Physicians Westphalia Lip, Robert-Schimrigk-Straße 4-6, 44141 Dortmund, Germany; 8 Regional Association of Statutory Health Insurance Physicians North Rhine, Tersteegenstraße 9, 40474 Duesseldorf, Germany; 9 Zentralinstitut für die Kassenärztliche Versorgung in der Bundesrepublik Deutschland, Salzufer 8, 10587 Berlin, Germany

**Keywords:** Indicators, Quality, Coordination, Ambulatory care sensitive conditions, Ambulatory care, Quality circles, Interdisciplinary, Accountable care, Germany

## Abstract

**Background:**

In settings like the ambulatory care sector in Germany, where data on the outcomes of interdisciplinary health services provided by multiple office-based physicians are not always readily available, our study aims to develop a set of indicators of health care quality and utilization for 14 groups of ambulatory-care-sensitive conditions based on routine data. These may improve the provision of health care by informing discussions in quality circles and other meetings of networks of physicians who share the same patients.

**Methods:**

Our set of indicators was developed as part of the larger Accountable Care in Deutschland (ACD) project using a pragmatic consensus approach. The six stages of the approach drew upon a review of the literature; the expertise of physicians, health services researchers, and representatives of physician associations and statutory health insurers; and the results of a pilot study with six informal network meetings of office-based physicians who share the same patients.

**Results:**

The process resulted in a set of 248 general and disease specific indicators for 14 disease groups. The set provides information on the quality of care provided and on patient pathways, covering patient characteristics, physician visits, ambulatory care processes, pharmaceutical prescriptions and outcome indicators. The disease groups with the most indicators were ischemic heart diseases, diabetes and heart failure.

**Conclusion:**

Our set of indicators provides useful information on patients’ health care use, health care processes and health outcomes for 14 commonly treated groups of ambulatory-care-sensitive conditions. This information can inform discussions in interdisciplinary quality circles in the ambulatory sector and foster patient-centered care.

**Supplementary Information:**

The online version contains supplementary material available at 10.1186/s12913-022-08327-1.

## Background

Audit and feedback in health care can be used as a strategy to improve professional practice and the quality of care [1]. It is based on the idea that health care professionals will aim to modify their behavior when feedback shows that the results of their clinical practice differ substantially from a desired standard, metric or other benchmark. An important form of audit and feedback are quality circles, which consist of physicians meeting in small groups to reflect upon common practice and tackle common problems [2]. Such meetings have been shown to be effective when participants are provided with individualized feedback on their own practice and given the opportunity to compare this feedback to metrics of group performance [3]. Providing such feedback, however, requires having measures, or indicators, of the quality of health services that allow for processes and outcomes of care relevant to the discussion group to be assessed, compared and ultimately improved [4, 5]. The provision of such feedback must be tailored and timely.

Often, quality indicators are based on patient outcomes. These alone are not meaningful; further information is needed to evaluate the results of professional practice and change behavior. In Germany, a few initiatives born by health insurance claims or regional health care projects have developed indicator sets on certain diseases, health care performance of networks or specific regional projects [6–8]. Also in Switzerland an indicator list for primary care was developed [9]. However, to date, no mutually agreed set of indicators has been set up or reported to enable informed discussions in interdisciplinary quality circles in ambulatory care in a regular manner that incorporates cooperation and coordination among the health care providers. Ambulatory-care-sensitive hospitalizations, for instance, are events that are assumed to be avoidable if timely and effective ambulatory treatment is available. It is well known that not only ambulatory care determines the number of hospitalizations due to ambulatory-care-sensitive conditions [10]. The condition of patients, disease prevalence, socioeconomics, supply side and other factors confound the relationship [11–14]. It seems reasonable to also look at patient characteristics, as well as processes and pathways of care to gain insights into the quality of care.

Ambulatory care differs from hospital care insofar as it must address a wider range of patient needs, and patients often have multiple problems where desirable outcomes are contested [15]. Approaches of assessing the quality of ambulatory care therefore differ from those used to assess hospital care [16]. Outcomes in ambulatory care are fairly unclear since many issues are less measurable compared to hospital care.

Multiple diseases and their interplay is relevant in the treatment and discussion of patient outcomes [17]. An interdisciplinary approach is often needed to coordinate care among providers. Indeed, in many cases, physicians are not informed about the whole patient pathway: they do not know which providers are consulted by patients, the treatments conducted, and medication received. In Germany, where there is no formal system of gatekeeping to office-based specialist care and patients have a free choice of primary care and specialist physicians, this information is regularly lacking. In addition, no electronic health records or comparable sources of joined-up information are currently available [18]. This situation has led to a lack of information and coordination among providers. Physicians regularly only have insights into their data perspective, but information on the patient pathways beyond physicians’ and sectors’ boundaries is hampered.

This study therefore choses a patient-focused view. It does not aim to develop indicators to describe the treatment of a single disease but focuses on multiple ambulatory-care-sensitive diseases. It aims to reflect the reality where patients face multiple diseases, where a discussion of physicians may involve the interplay of different diseases, and coordination is needed. We chose to include diseases in our study that are ambulatory-care-sensitive, have a high prevalence, and are therefore frequently treated by ambulatory physicians. The diseases are ambulatory-care-sensitive insofar that for the set of conditions timely and effective outpatient care can help to reduce the risks of hospitalization by either preventing the onset of an illness or condition, controlling an acute episodic illness or condition, or managing a chronic disease [12]. Ambulatory care for these diseases is of the utmost importance. Indicators for quality improvements in ambulatory care are missing yet, audit and feedback may improve health outcomes. Furthermore, the diseases included in our set are treated by different disciplines and are therefore suitable for an interdisciplinary discussion. Fourteen ambulatory-care-sensitive conditions based on a German catalogue of conditions [19] meet these criteria. The 14 disease groups include ischemic heart diseases (International Classification of Diseases (ICD) I20–I25), heart failure (I50), other diseases of the heart and circulatory system (I05, I06, I09, I08, I49, I48, I67, I70, I73, I78, I80, I83, I87, I95, R00, I42, I74), chronic obstructive pulmonary disease (J44, J47), mental and behavioral disorders due to use of alcohol and opioids (F10, F11), dorsopathies/back pain (M42, M47, M53, M54, M50, M51), bronchitis (J20, J21, J22, J40, J41, J42, J43), hypertension (I10–I15), gastroenteritis and other diseases of the intestine (K52, K57, K58, K59), intestinal infectious diseases (A00–A09), influenza and pneumonia (J10–J16, J18), infections of the ear, nose and throat (H66, J01–J04, J06, J31, J32, J35, H65, H73, R07.0), depressive disorders (F32, F33), diabetes mellitus (E10, E11, E13, E14, E16) and gonarthrosis (M17).

Hospitalizations for these fourteen conditions indicate a progressed disease state [20]. All these conditions have an estimated high level of preventability of hospitalization [19], which means that access to effective ambulatory care is assumed to have a relevant impact on the number of hospitalizations with these conditions.

The development of indicators in this study focuses on those indicators that are derivable using routine data (specifically insurance claims data) because of their specific advantages for health services research [21, 22]: Detailed patient information such as age, gender, morbidities, mortality and health care utilization can be used in a pseudonymized manner and across providers seen in a patient-focused view. It furthermore includes ambulatory and hospital data and prescriptions with provider identifiers. Data generation costs are comparably low as data have been collected for their primary purpose of reimbursement. The risk of data manipulation is assumed to be minimal since data is formally approved [22]. Moreover, a large patient collective can be included at ease in assessments.

The aim of this study is to develop an indicator set to serve in interdisciplinary discussions within network meetings or quality circles aiming to improve coordination among physicians. It focuses on the treatment of ambulatory-care-sensitive conditions and indicators that can be computed using routine data. The indicators are developed within the German setting and the project Accountable Care in Deutschland (ACD) in a pragmatic consensus process. However, the set can be transferred to other settings. The following chapters describe the method, as well as core questions when developing indicator sets and results of the consensus group process.

## Methods

**Fig. 1:**
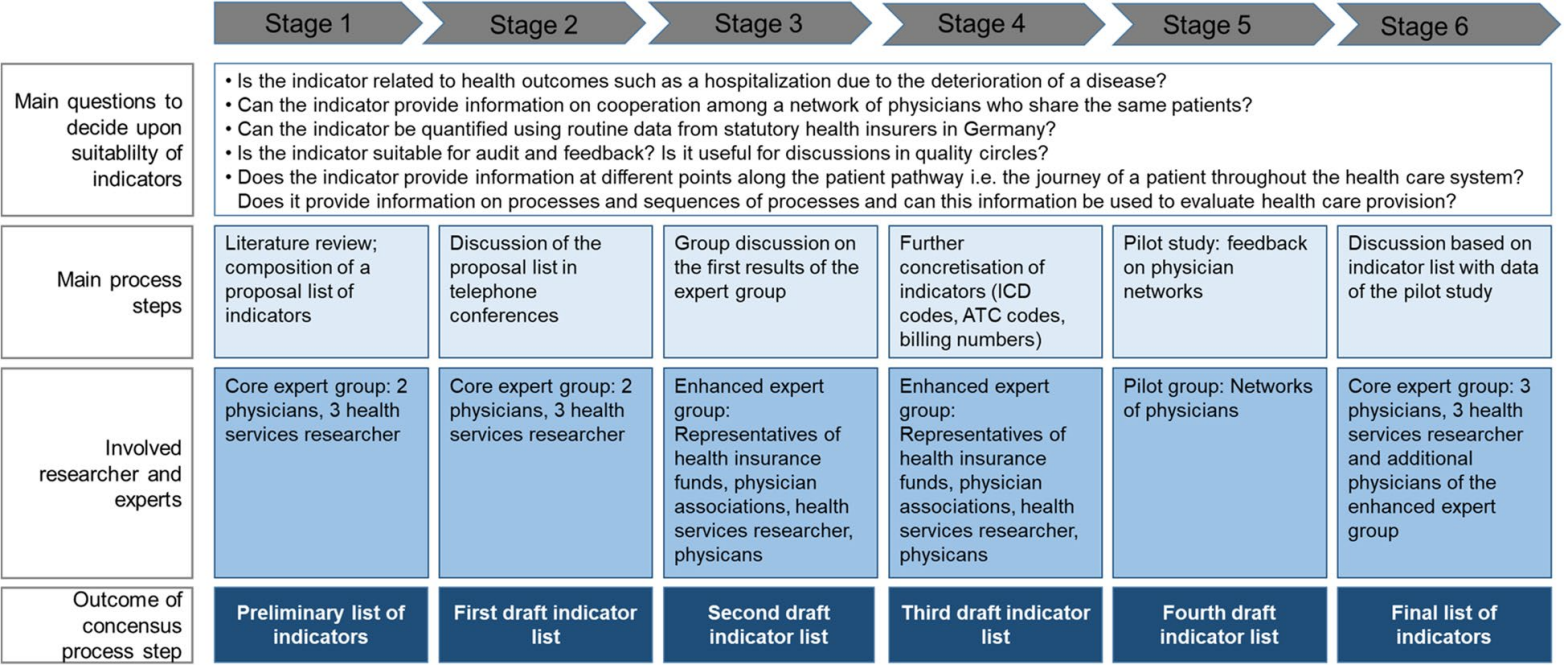
The process used for developing a set of interdisciplinary quality indicators

We developed a set of interdisciplinary indicators for 14 ambulatory-care-sensitive conditions using a pragmatic consensus process that took place from July 2017 through October 2018 and consisted of the six stages shown in Fig. 1. The process drew upon a combination of evidence from the literature and expert opinions, and followed the framework and research methods set out by Campbell et al. [23, 24] for developing and implementing quality indicators in primary care.

The consensus process involved a core expert group (CEG) and an enhanced expert group (EEG). The former consisted of two to three physicians and three health services researchers to ensure adequate representation of expertise in medicine and data structure. The latter consisted of the core expert group and representative from physician associations, statutory health insurers and health services researchers with expertise in medicine, reimbursement specifics, data structure and routine data analysis. In our study, no patient representative was involved in the development process because the indicators derived were aimed to enable discussions among physicians based on professional standards, medical guidelines, health outcomes and information on the patient pathway through the health care system.

To decide upon the suitability of an indicator in terms of acceptability, feasibility, reliability, sensitivity to change and validity [15, 23], the CEG developed the following research questions:

- Is the indicator related to health outcomes such as a hospitalization due to the deterioration of a disease?
- Can the indicator provide information on cooperation among a network of physicians who share the same patients?
- Can the indicator be quantified using routine data from statutory health insurers in Germany?
- Is the indicator suitable for audit and feedback? Is it useful for discussions in quality circles?
- Does the indicator provide information at different points along the patient pathway, i.e. the journey of a patient throughout the health care system? Does it provide information on processes and sequences of processes, and can this information be used to evaluate health care provision?

The indicators were used as part of the larger ACD project to provide regular feedback to informal networks of physicians who shared the same patients on the health care they provided to these patients [25]. The informal networks consist of 20 to 120 physicians from different disciplines who are involved in the treatment of the included patients [26]. These physicians receive quarterly feedback in the form of selected indicators on their own treatment activities and those of their colleagues in the network and are invited to voluntary and moderated network meetings every six months. The indicators should thus allow an informed dialogue of the network members at the meetings.

The routine data used in this study was collected by three insurance claims, aggregated and sent to the research facility. Patient data was pseudonymised, and the physician’s office IDs were sent un-pseudonymised. A Trust Centre used a reversible dual technique to pseudonymise the physician data. The IDs of physicians who were part of a physician network was pseudonymised/replaced with the pseudonym from key lists. The pseudonymised data was secured and transferred to the analysts that calculated the indicator values in the study.

In the first stage of the consensus process, a preliminary set of indicators was generated by the expert group (CEG). This set was based on a structured literature review and quality indicators available from national guidelines and other settings (see Additional file 1: A1). Three researchers were involved in this stage of the consensus process. Disagreements upon inclusion of indicators were resolved by discussions of the reviewers. In the second stage of the development process, the preliminary set was distributed among all members of the CEG and discussed by them in telephone conferences using the above-mentioned five research questions. The resulting first draft of the indicator set was discussed in the third stage among the EEG, also using the five research questions. The usability of the indicators and coding were discussed within this group until consensus was reached, and a second draft of the indicator set was generated. This discussion identified further questions about quantifying the indicators – for example, which diagnoses, and operation and remuneration codes were relevant in this regard. These questions were considered in the fourth stage, first by the CEG drawing upon the expertise of the EEG, leading to a third draft of the indicator set.

In the fifth stage**,** we conducted a pilot study using this third draft of the set. Two informal networks of physicians derived from routine data and not part of the expert group chose diabetes, depression, hypertension, bronchitis and COPD as exemplary disease groups. They were provided by us during a series of meetings with feedback, using the third draft of the set, on the care they had provided to the patients they share. During the meetings, the physicians discussed the practicability of using these indicators to enrich such discussions about their performance, and provided us with comments in this regard. This information was subsequently used to inform the sixth stage: the CEG once again discussed the usefulness of the indicators, using the third draft of the indicator set, the comments provided by the physicians who participated in the pilot study, and information on indicator values from five informal networks of physicians who shared the same patients. The five networks were chosen by the study group. The pilot study values were used to check for plausibility and coding specifics. Lastly, the resulting set of indicators was approved by all members of the CEG and the EEG.

Compared to prior indicator lists our indicator set for the 14 ambulatory-care-sensitive conditions is not limited to one disease or one physician group, but allows informed discussions of physicians that treat the same patient groups with highly prevalent diseases. It addresses the results of health care provision for patients with multiple diseases and in need of interdisciplinary health care. The set of indicators thus provides information on the coordination and cooperation of physicians along the patient pathway.

## Results

### Results of the consensus process

The structured literature search conducted in stage 1 identified a range of relevant sources of indicators, including national clinical guidelines and the German indicator sets “Qualitätsindikatoren in der ambulanten Versorgung” (QISA) and “Ambulante Qualitätsindikatoren und Kennzahlen” (AQUIK). Further sets of indicators derived from health care systems in other countries and included the National Quality Forum (NQF) set, the Quality Outcomes Framework (QoF) indicator set, the Agency for Healthcare Research and Quality (AHRQ) set, Diabetes Quality Indicator Set (DQIS) set, the Pharmacy Quality Alliance (PQA) measures, the Physician Quality Reporting System (PQRS) set, the National Quality Measures Clearinghouse (NQMC) set, the Rand Assessing Care of Vulnerable Elders (ACOVE) 3 set, and indicators of the National Committee for Quality Asssurance (NCQA) [6, 27–35]. We extracted indicators for the diseases included in our study, resulting in a set of 229 indicators (see Table 1). In stage 2 of the consensus process, the CEG discussed the set of 229 indicators during four telephone conferences, and ultimately excluded 101 of these. One challenge of this stage was to add further information on the given indicators in order to quantify them in routine data, to aggregate redundant indicators and compare the indicators with the recommendations in medical guidelines. Indicators were excluded if they were redundant or if data restrictions did not allow for the indicator to be operationalized adequately. This was the case when diagnoses were not derivable using ICD-10 codes and when clinical values were relevant for an indicator. Furthermore, indicators were dropped from the set if a service was included as an optional service in a lump sum payment (i.e., by diagnosis-related group), or if the relevant observation time exceeded one year. Indicators were also dropped from the set if the effectiveness of a therapy was debatable, or no evidence of effectiveness was available. The CEG decided to modify seven indicators to the German setting and to gain further information on the relevance of eight indicators.

**Table 1:** Number of indicators per disease group and process stage

	Stage	1	2	3	4	5	6
	List	Preliminary list of indicators	First draft	Second draft	Third draft	Fourth draft	Final list of indicators
00	General indicators		7	7	7	7	7
01	Ischemic heart diseases	47	57	22	24	23	23
02	Heart failure	20	31	19	20	22	22
03	Other diseases of the circulatory system	3	9	7	8	10	10
04	Bronchitis & COPD	21	34	19	21	24	24
05	Mental and behavioral disorders due to use of alcohol or opioids	2	8	8	10	11	11
06	Back pain [dorsopathies]	4	10	13	9	13	13
07	Hypertension	10	16	11	12	14	14
08	Gastroenteritis and other diseases of intestines	2	8	8	11	13	13
09	Intestinal infectious diseases	0	6	7	8	10	10
10	Influenza and pneumonia	10	16	12	12	14	14
11	Ear nose throat infections	36	42	12	13	15	15
12	Depressive disorders	15	21	14	13	15	15
13	Diabetes mellitus type 1 and type 2	54	60	18	21	44	44
14	Gonarthrosis [arthrosis of knee]	5	11	11	11	13	13
	Sum	229	336	188	200	248	248
Number of indicators added from the literature within the stage		229	0	0	0	0	0
Number of indicators removed within the stage		0	101	148	1	0	0
Number of indicators added for patient pathway information		0	91	0	13	37	0

The resulting set of indicators was subsequently expanded by the researchers involved to include indicators that would give information on patient visits to general practitioners (GPs) and office-based specialists. These indicators included information on which of the relevant specialists had been involved in the treatment of a given patient and whether and how many multiple specialists from the same specialty area had been consulted by a given patient during the observation period. Physician groups defined as relevant for the treatment of a disease are listed in the Additional file 1: A2. Moreover, based on the group discussions an indicator was added that provided information on the number of calendar quarters during which patients with one of the included diseases saw at least one physician or specialist relevant for their disease. In this stage, the expert panel discussed if a maximum number of indicators should be defined. The expert panel decided not to limit the number of indicators in order to provide physicians a comprehensive set of indicators that allows the networks to discuss upon topics most relevant in the specific network. In total, the first draft of the set of indicators comprised 336 indicators.

In stage 3, the EEG focused on the plausibility of the information provided by each of these indicators. A total of 148 indicators were dropped from the set during this stage due to concerns among the experts about being able to quantify certain indicators using routine data, as well as about missing evidence on the relevance of indicators. In this stage, the EEG faced the challenge to decide the extent to which an indicator was quantifiable in routine data or dropped from the list. The discussion yielded a second draft containing 188 plausible indicators.

In stage 4 the members of the EEG searched for further information on coding and reimbursement rules for the indicators with the aim to operationalize the indicators with German routine data. A major concern in this stage was to ensure that the indicators were relevant within the current coding system. This stage led to additional indicators being added to the set to gain precision with regard to the information provided, yielding a third draft of the set comprising 200 indicators.

The results of our pilot study suggested that the indicators were useful in group discussions. They found the summary table listing all available indicators helpful to gain an overview over their indicator values. Many of the indicators were discussed in depth, and others stimulated detailed discussions on topics such as the strengths and weaknesses of regional health care provision. In the group discussions, physicians were given feedback forms listing indicators from the third draft of the indicator set for a selection of disease groups, including diabetes, depression, hypertension, bronchitis, and COPD. The aggregation level of the information provided was at the network level, comparing data of the given network with other networks. Physicians participating in the pilot study were also interested in the results of patients treated in their practices and how these compared to those of the other patients within the given network. Besides, they were not only interested in physician visits, but also on the share of patients who had been referred to a specialist. Therefore, indicators for all relevant specialists’ visits for all disease groups were added to the fourth draft (i.e., pre-final) set of indicators. The physicians participating in the pilot study also suggested that information on diabetes-related indicators should be reported separately for patients with type 1 or type 2 diabetes. These were added to the indicator set and resulted in a pre-final set of 248 indicators.

This list was finally proofed by the CEG and additional physicians of the EEG in the project. The list of indicators was confirmed, leading to a final set consisting of 248 indicators describing the treatment of patients in 14 disease groups along the entire patient pathway (see Additional file 1: A3 and A4).

### Description of the indicators derived through the consensus process

The 248 indicators were derived at the patient level to ensure that all treatments beyond the sector boundaries of the health care system would be accounted for. The observation period for each of the indicators was one year. The set comprises indicators as recommended in medical guidelines and avoidable events and processes, such as potentially avoidable hospitalizations or opioid prescriptions for patients with back pain.

Exemplary for type 2 diabetes patients the indicator set includes amongst other indicators the following information: For all patients with type 2 diabetes the rate of patients with a rehospitalisation due diabetes within the observation period is reported for all networks as well as the regional average. In case the result of this outcome indicator differs to those values of the region, physicians may also compare their performance on the indicators recommended in clinical guidelines such as the biannual funduscopy or laboratory tests to be conducted regularly. Additionally the indicator set offers information about the share of patients with type 2 diabetes with their frequency of physician visits. They are for instance informed about the share of patients that visited any physicians’ office in only one quarter of the year. Furthermore, the share of patients visiting a diabetic specialist practice is reported as well as the share of patients visiting other relevant specialists within the observation period. Addressing coordination problems, also the share of patients visiting more than one physician of one specialist group were reported. Physicians were thus able to discuss about the local specific health care provision of their commonly treated type two diabetes patients. Consequently, they could work on specific problems of their network.

The indicator set offers a minimum of 10 indicators per disease group to provide information on the characteristics of patients, their pathways through ambulatory care, their treatment within ambulatory care, and outcome indicators often occurring in the hospital sector. The number of indicators in each disease group differs substantially because it was not always possible to operationalize an indicator using routine data. Among the 14 disease groups, ischemic heart diseases, diabetes and heart failure had the largest number of indicators. For some disease groups, it proved difficult to operationalize disease severity and stages in routine data, for instance for diabetes, resulting in a limited number of severity-specific medication indicators. Additionally, participants in the consensus process raised concerns for depression about the coding of the disease and its severity, which have to be taken into account when investigating the indicators reported. Depression may not be coded adequately in routine data due to disease-specific reasons such as the stigma associated with mental illness [36].

The indicators in each disease group can be subdivided into the following three categories: patient characteristics, patient pathways (i.e., physician visits, ambulatory care processes, and pharmaceutical prescriptions), and outcome indicators. An overview of the number of indicators per category and disease group is provided in Table 2.

**Table 2:** Categorization of indicators in the final indicator set

	Characteristics of the network	Characteristics of patients	Physican visits	Ambulatory care processes	Prescriptions	Outcome indicators	Sum
General indicators	4	2	0	1	0	0	7
Ischemic heart diseases	1	4	4	3	7	4	23
Heart failure	1	3	5	3	7	3	22
Other diseases of the circulatory system	1	3	4	0	0	2	10
Bronchitis & COPD	1	4	5	5	4	5	24
Mental and behavioral disorders due to use of alcohol or opioids	1	3	4	1	0	2	11
Back pain [dorsopathies]	1	3	4	2	1	2	13
Hypertension	1	3	4	0	4	2	14
Gastroenteritis and other diseases of intestines	1	3	4	0	2	3	13
Intestinal infectious diseases	1	3	4	0	0	2	10
Influenza and pneumonia	1	3	5	1	2	2	14
Ear nose throat infections	1	3	4	0	5	2	15
Depressive disorders	1	3	4	1	4	2	15
Diabetes mellitus, type 1	1	4	5	6	2	3	21
Diabetes mellitus, type 2	1	4	5	6	4	3	23
Gonarthrosis [arthrosis of knee]	1	3	4	0	1	4	13
Sum of indicators	19	51	65	29	43	41	248

Indicators describing patient characteristics of the patient population investigated include patient age, gender, multimorbidity, participation in disease management programs, and mortality during the observation period.

For each disease group a set of relevant specialists was identified with the aim to indicate visits at GPs and at each of the relevant specialists for every disease group as well as the share of patients with referral to each specialist group. In order to provide information on patient pathways within the health care system, physician visit indicators also provide information on (a) the number of patients who visited more than one specialist within the same field of specialization and (b) the number of quarters patients visited at least one disease-relevant physician during the observation year.

Ambulatory care processes and pharmaceutical prescriptions are depicted for all disease groups where relevant indicators were available. This includes, for instance, the share of patients with back pain who underwent an X-ray examination during the observation period. Additionally, the share of back pain patients who received opioids is shown.

Moreover, for all disease groups at least two outcome indicators were provided: the rate of patients who were not hospitalized due to one of the included diseases during the observation period, adjusted for age and gender, as well as the adjusted rate of patients with less than two such hospitalizations during the observation period. When available, additional outcome indicators were part of the set, such as survival after a percutaneous coronary intervention.

## Discussion

We used a pragmatic, six-stage consensus process within the broader Accountable Care in Deutschland (ACD) project to develop a set of 248 indicators of the quality and use of interdisciplinary health care for 14 groups of ambulatory-care-sensitive conditions. The indicators are based on routine data from an observation period of one year to allow for an in-depth view on health services provided. The indicator list adds to the literature of indicator sets based on routine data [6–9]. The indicators can be used to foster patient-focused care by informing interdisciplinary discussions in quality circles of physicians who share the same patients, as well as the practice of individual physicians in order to investigate the results of their common and coordinated care.

Indeed, in the pilot study of our indicators, physicians commented that they were interested in using the indicators for both purposes. They were interested in a benchmark for the indicator values to be able to interpret the results of the common work, and to see the results for a group of patients they were even more responsible for. The study group also discussed to provide more detailed information such as the names of hospitals patients were admitted to. This would have allowed physicians to also invite hospital representatives to group discussions to commonly discuss the situation at a local level. The demand for specific information such as patient or hospital identities would allow specific recommendations to action and change but raised questions of data protection. Participants in the consensus process aimed to develop indicators that would be specific while meeting data protection of individual entities. The study group had to deal with this trade-off between very specific information and data protection regulations when developing the indicators.

Some physicians, such as psychotherapists, treat a comparatively small number of patients throughout any given year. This raises the question of which diseases to focus on when developing sets of indicators for interdisciplinary discussions among physicians. In the ACD project, we chose diseases that had a high prevalence, required interdisciplinary treatment and showed potential for improvements in quality of care. By focusing on the 14 diseases included in our study, we may have created a situation in which physicians may have not felt responsible for the results for each of the diseases or indicators and may find interdisciplinary discussions not productive. An ophthalmologist may not feel accountable for the depression of his patient but may have treated him related to his diabetic retinopathy. However, specifically the interdisciplinary treatments and management of chronic diseases are seen as reasons for problems in the health provision and reason for adverse events [37]. The 14 disease groups included may lead to a high number of involved disciplines. The whole set of indicators may consequently not be relevant for all physicians and specialists. An alternative would be to only focus on one disease and only to include physicians that inevitably should cooperate in the treatment of patients with the specific disease. One important argument against this approach is the focus on patients who have more than one disease. Their health care outcomes may not only be dependent on the treatment of one disease, but the interplay of different disciplines [38, 39]. Indeed, in the Accountable Care study first results show that more than 50% of the patients have more than one of the fourteen diseases. The patient-focused view is important to report on patient relevant results. However, it also brings up concerns regarding the accountability of results.

Another important aspect in the development of the set was the overall objective of the indicator set to be provided within audit and feedback processes. When results on performance indicators are made available to health professionals, different approaches and claims are made to the approach: It may include very detailed recommendations on processes to proceed. This is, for instance, the case in feedbacks provided in disease management programs, where physicians receive lists of patients for which specific processes should be conducted [40]. A further approach is to define a target range for each indicator and to indicate the results of physicians, using colors and icons. For instance, the results of physicians of the QUATRO networks in Germany are accompanied by icons in the indicator sets [41]. Both approaches need a set of indicators with clear targets, critical values and existing evidence on these indicators [42]. A further approach, which was chosen in this study, is to provide an exhaustive set of indicators where physicians themselves can benchmark their results and discuss on differences in outcomes. A complete list can be perceived as flood of information for many physicians. One solution chosen in our project was to sum up feedback reports with a selection of indicators to generate interest about further indicators. Furthermore, only two disease groups were reported in each quarterly feedback report. The aim was to strengthen the interest in the joint work and to provide a basis for further informed discussions about the joint work.

### Limitations

Our study has a number of important limitations. First, we did not use formal consensus methods like a Delphi process to develop our set of indicators, potentially leading to biases associated with certain forms or social interaction or subordination among the participants. While we sought to minimize the risk of such bias by drawing additionally upon multiple sources of information, including existing lists of indicators and structured reviews of the published evidence, future projects in this area may wish to employ more iterative methods with controlled feedback, such as the nominal group technique [43]. In our study, we drew upon a pragmatic consensus method because we aimed to incorporate the existing evidence on quality assurance results with expert opinion. Within these expert group discussions, indicator sets were intensively discussed and amended so that indicators were appraised as clinically relevant and derivable in routine data and could inform about cooperation of physicians treating the same patients. Second, the experts involved in the development process had clinical expertise specifically in general practice, internal medicine, geriatrics, cardiology and emergency medicine, but were not representative for all groups of specialists.

Third, our set contains only those indicators that can be calculated using routine data from health insurance claims. While this has the advantage of readily available data of different health care providers, it would be useful if our set of indicators could draw additionally on clinical information from providers so that further outcome indicators could be added to the list. This is important because routine data has several pitfalls: for example, it provides information only related to the services that have been billed with the insurance claim and therefore does not capture services paid for out-of-pocket and over-the-counter medications [44]. In addition, aspects of health care provision such as physician-patient communication, patient experience, and patient satisfaction are not included in most forms of routine data. We nevertheless chose routine data as a starting point for our project because it is readily available and mostly reliable.

## Conclusion

Based on prior literature, existing quality indicators, an expert consensus process and a pilot study with networks of physicians in ambulatory care, a set of 248 indicators was identified. The set provides a broad description of patient health care use, processes as well as outcomes, for 14 commonly treated groups of ambulatory-care-sensitive conditions and general indicators which can be used for interdisciplinary network meetings.

Our set of indicators can be used to analyze health services provided in defined settings and identify differences in the services provided from a patient-focused perspective. It allows for further exploration of health services differences. The indicator set can be tested in audit and feedback projects to inform physicians in ambulatory care on the results of their work. It may be investigated if these indicators are suitable for discussions in interdisciplinary meetings and quality circles.

## Supplementary Information

**Additional file 1.**

## Data Availability

All data generated or analysed during this study are included in this published article.
