# Supplementary material for: Development of indicators to assess quality and patient pathways in interdisciplinary care for patients with 14 ambulatory-care-sensitive conditions in Germany
Source: BMC Health Serv Res. 2022 Aug 9;22:1015. doi: 10.1186/s12913-022-08327-1 (PMC9364554; doi:10.1186/s12913-022-08327-1)
Supplement: Supplementary file 1 — Additional file 1. [file 12913_2022_8327_MOESM1_ESM.pdf]

Appendix

A 1 – Information on the literature search of existing quality indicators

Literature Search on Quality Indicator Sets for Patients with the 14 Ambulatory-Care-Sensitive Conditions

Identification	Quality indicators on ambulatory care-sensitive conditions in the literature
Information Sources	Medline, EMBASE, the Cochrane collaboration and the internet
Screening	Additional screening of references, indicators lists and reference lists
Eligibility	Full-text articles with quality indicator sets were assessed for eligibility
Inclusion Criterion	Quality indicator sets for at least one of the included ambulatory care-sensitive conditions<br>- ischemic heart diseases<br>- heart failure<br>- other diseases of the heart and circulatory system<br>- chronic obstructive pulmonary disease and bronchitis<br>- mental and behavioral disorders due to use of alcohol and opioids<br>- dorsopathies/back pain<br>- hypertension<br>- gastroenteritis and other diseases of the intestine<br>- intestinal infectious diseases<br>- influenza and pneumonia<br>- infections of the ear, nose and throat<br>- depressive disorders<br>- diabetes mellitus<br>- gonarthrosis
Specification	Indicators were specified in terms their numerator, denominator, exclusion reason, limitation
Synthesis of results	All relevant indicators were listed in an proposal indicator list

A 2 – List of 14 disease groups with ICD codes

Nr.	Disease Group	ICD-10
1	Ischemic heart diseases	I20, I25.0, I25.1, I25.5, I25.6, I25.8, I25.9
2	Heart failure	I50
3	Other diseases of the circulatory system	I05, I06, I08.0, I49.8, I49.9, I67.2, I67.4, I70, I73, I78, I80.0, I80.80, I83, I86, I87, I95, R00.0, R00.2, R47.0
4	Bronchitis & COPD	J20, J21, J40-J44, J47
5	Mental and behavioral disorders due to use of alcohol or opioids	F10, F11
6	Back pain [dorsopathies]	M42, M47, M53, M54
7	Hypertension	I10-I15
8	Gastroenteritis and other diseases of intestines	K52.2, K52.8, K52.9, K57, K58, K59.0
9	Intestinal infectious diseases	A01, A02, A04, A05, A07-A09
10	Influenza and pneumonia	J10, J11, J13, J14, J15.3, J15.4, J15.7, J15.8, J15.9, J16.8, J18.0, J18.1, J18.8, J18.9
11	Ear nose throat infections	H66, J01-J03, J06, J31, J32, J35
12	Depressive disorders	F32, F33
13	Diabetes mellitus type 1 and 2	E10.2-E10.6, E10.8, E10.9, E11, E13.6, E13.7, E13.9, E14, E16.2
14	Gonarthrosis [arthrosis of knee]	M17.0, M17.1, M17.4, M17.5, M17.9

A3 –Table of Indicators

Group	Ind. No.	Cate gory*	Name of the indicator(s)	Numerator	Denominator
General indicators	G1	V	Number of distinct patients per network	Number of jointly treated patients with at least one associated disease in the network	Total number of shared patients with at least one ACD disease
	G2	V	Average number of shared patients	Average number of patients commonly treated by two physicians in the network	
	G3	V	Number of physicians in the network (per specialist group)	Number of physicians	
	G4	V	Loyalty of patients to network physicians	Network patients treated by physicians within the network	Network patients treated by physicians outside the network
	G5	G	Proportion of male / female patients	Proportion of male or female patients	Total number of patients with at least one ACD disease
	G6	G	Number of patients in age groups (<45, 45-54, 55-64, 65-74, 75-84, >85)	Number of patients in an age group	Total number of patients with at least one disease
	G7	V	Rate of patients older than 65 years who received influenza vaccination in the observation year	Patients older than 65 years with the ACD disease groups who received at least one influenza vaccination (EBM 89111 / 89112 or ATC J07BB) during the observation year	Patients over 65 years of age with at least one ACD disease
Ischemic heart diseases	D1-1	V	Number of jointly treated patients with ischemic heart diseases in the network	Number of patients shared with a disease group	
	D1-2	V	Number of treated patients with coronary heart diseases	Number of patients with the disease group	
	D1-3	G	Multimorbidity rate of CHD patients	Patients with more than one disease of the 14 disease groups	Patients with ischemic heart diseases
	D1-4	G	Share of patients enrolled in the disease management program "ischemic heart disease"	Enrollment in the disease management program	Patients with ischemic heart diseases
	D1-5	G	Mortality rate of CHD patients within the observation year	Patients who died within the observation period	Patients with ischemic heart diseases
	D1-6	V	Rate of patients in the disease group with at least one billing contact with a specialist in general medicine / general practitioner (family doctor) / family internal medicine / internist / specialist internal medicine / nuclear medicine / psychosomatic medicine and psychotherapy / physical and rehabilitative medicine / cardiology within the observation year	Patients for whom at least one billing item was billed by a specialist in the respective specialty group	Patients with ischemic heart diseases
	D1-7	V	Rate of patients in the disease group with at least one billing contact to each of the above specialist groups with referral within the observation year.	Rate of patients with at least one billing contact to each of the above specialist groups with referral within the observation year.	Patients with ischemic heart diseases
	D1-8	V	Proportion of patients with billing contact in at least a) one, b) two, c) three d) four of four quarters for above-mentioned physician groups	Proportion of patients with billing contact in at least a) one, b) two, c) three d) four of four quarters for above-	Patients with ischemic heart diseases

Group	Ind. No.	Cate gory*	Name of the indicator(s)	Numerator	Denominator
				mentioned physician groups	
	D1-9	V	Rate of patients in the disease group with at least one billing contact with two different specialists in the above-mentioned disciplines (each per discipline) within the year of observation	Patients for whom at least one billing item was billed by two different specialists in the respective specialty with different facility identities and with the type of physician utilization "original" in the observation year	Patients with ischemic heart diseases
	D1-10	V	Patients with ischemic heart diseases with an influenza vaccination	CHD patients for whom at least one outpatient influenza vaccination was billed (EBM 89111 / 89112) or who were prescribed ATC J07BB at least once.	Patients with ischemic heart diseases
	D1-11	V	Patients with ischemic heart disease who participated in a disease management training	CHD patients for whom training was billed (EBM region specific)	Patients with ischemic heart diseases enrolled in the disease management program
	D1-12	V	Rate of CHD patients whose LDL and HDL levels were examined at least once in the observation year	Ambulatory billed position for the named evaluations (GOP 32060 or (32061 and 32062 and 32063)).	Patients with ischemic heart diseases
	D1-13	V	Rate of CHD patients who were prescribed statins at least once per quarter during the year of observation	Prescription of statins each quarter (ATC C10AA*, C10BA* or C10BX*).	Patients with ischemic heart diseases
	D1-14	V	Rate of CHD patients who were prescribed statins at least once during the observation year	Prescription of statins (ATC C10AA*, C10BA* or C10BX*).	Patients with ischemic heart diseases
	D1-15	V	Prescription of statin, betablocker and ACE inhibitor/ AT1 receptor blocker	Prescription of statin (ATC C01AA *, C10BA*, C10BX*), beta-blocker (ATC C07A*) and ACE inhibitor/ AT1 receptor blocker (ATC C09A*, C09B*, C09C*, C09D*).	Patients with ischemic heart diseases
	D1-16	V	Patients with MI and prescription of beta-blocker	CHD patients with myocardial infarction (ICD I21*, I22*, I23* as main or secondary inpatient diagnosis) who were prescribed beta-blockers (ATC C07*) in the same or following quarter (prescription date). For patients with multiple myocardial infarctions, only the first is considered. Note: Patients with index event in the 4th quarter not considered.	Patients with ischemic heart diseases with a MI (ICD I21.-, I22.-, I23.- discharge or secondary diagnosis in the hospital) during the first three quarters of the observation year.
	D1-17	V	Patients with MI and prescription of ACE inhibitor or ARB	CHD patients diagnosed with myocardial infarction (inpatient as principal or secondary diagnosis ICD I21*, I22*, I23*) and prescribed ACE inhibitor/ARB therapy (ATC C09A / C09B / C09C / C09D) in the same or subsequent quarter. For	Patients with ischemic heart diseases with a MI (ICD I21.-, I22.-, I23.- discharge or secondary diagnosis in the hospital) during the first three quarters of the observation year.

Group	Ind. No.	Cate gory*	Name of the indicator(s)	Numerator	Denominator
				patients with multiple myocardial infarctions, only the first one is considered. Patients with myocardial infarction in the 4th quarter not considered.	
	D1-18	V	Proportion of CHD patients with type 2 diabetes who were prescribed lipid-lowering agents in the year of observation.	CHD patients who are also diabetes type 2 patients and were prescribed lipid-lowering agents (ATC C10A / C10B) at least once during the observation year.	CHD patients with at least 2 confirmed outpatient or one inpatient E11.* diagnosis/s.
	D1-19	V	Rate of CHD patients with hypertension who were prescribed antihypertensives at least once during the observation year.	CHD patients with hypertension with at least one prescription ATC C02 / C03 / C07 / C08 / C09 in the observation year.	Patients with ischemic heart diseases and hypertension
	D1-20	O	Proportion of CHD patients who received PCI in the observation year and survived the following month	CHD patients who received PCI at least once during the observation year (OPS 8-837) survived at least 30 days	Patients with ischemic heart diseases with PCI in the hospital (OPS code 8-837* or 1-275*) in the first 11 months of the observation year
	D1-21	O	Proportion of CHD patients who received PCI in the observation year and did not die in the same six months	CHD patients who received PCI at least once in the observation year (OPS 8-837*, 8-83d*) and did not die in the same six months	Patients with ischemic heart diseases with PCI in the hospital (OPS code 8-837* or 1-275*) in the first 6 months of the observation year
	D1-22	O	Proportion of CHD patients without hospitalization for ischemic heart disease within the observation year (risk-adjusted).	Patients without an inpatient hospital case in the observation year due to ICDs (principal diagnosis) of the corresponding disease group	Patients with ischemic heart diseases
	D1-23	O	Proportion of patients with fewer than two hospitalizations for ischemic heart disease within the observation year (risk-adjusted).	Patients with fewer than two inpatient hospitalizations due to ICDs (principal discharge diagnosis) in the disease group	Patients with ischemic heart diseases
Heart failure	D2-1	V	Number of jointly treated patients with heart failure in the network	Number of patients	
	D2-2	V	Number of treated patients with heart failure	Number of patients	
	D2-3	G	Multimorbidity rate of heart failure patients	Patients with more than one disease of the 14 disease groups	Patients with heart failure
	D2-4	G	Mortality rate of heart failure patients within the observation year	Patients who died within the observation period	Patients with heart failure
	D2-5	V	Rate of patients in the disease group with at least one billing contact with a specialist in general medicine / general practitioner (family doctor) / family internal medicine / internist / internal medicine / cardiology / nephrology / psychosomatic medicine and psychotherapy / radiology / physical and rehabilitative medicine within the observation year	Patients for whom at least one billing item was billed by a specialist in the respective specialty group	Patients with heart failure

Group	Ind. No.	Cate gory*	Name of the indicator(s)	Numerator	Denominator
	D2-6	V	Rate of patients in the disease group with at least one billing contact with two different specialists in the above-mentioned disciplines (each per discipline) within the year of observation	Patients for whom at least one billing item was billed by two different specialists in the respective specialty with different facility identities and with the type of physician utilization "original" in the observation year	Patients with heart failure
	D2-7	V	Rate of patients in the disease group with at least one billing contact to each of the above specialist groups with referral within the observation year.	Rate of patients with at least one billing contact to each of the above specialist groups with referral within the observation year.	Patients with heart failure
	D2-8	V	Proportion of patients with billing contact in at least a) one, b) two, c) three d) four of four quarters for above-mentioned physician groups	Proportion of patients with billing contact in at least a) one, b) two, c) three d) four of four quarters for above-mentioned physician groups	Patients with heart failure
	D2-9	V	Rate of heart failure patients with inpatient hospitalization for heart failure and follow-up with an outpatient physician in the same or subsequent quarter	Heart failure patients who had an inpatient hospitalization with principal diagnosis ICD I50* and were coded I50* by an outpatient physician in the same quarter or in the following quarter.	Heart failure patients who had an inpatient hospitalization in the first, second, or third quarter of the observation period with principal discharge diagnosis I50*.
	D2-10	V	Rate of heart failure patients with at least one laboratory service (potassium / sodium / creatinine) in the observation year	Patients restricted to index event in penultimate observation quarter at the latest.	Patients with heart failure
	D2-11	V	Rate of heart failure patients who received at least one echocardiogram with a cardiologist in the observation year	Heart failure patients who were billed for at least EBM billing item EBM 13545 or 13550 (echocardiography) by a specialist in cardiology within the observation year	Patients with heart failure
	D2-12	V	Rate of heart failure patients with at least one home visit with a diagnosis of heart failure in the observation year	Number of home visits (EBM 01410 / 01411 / 01413 / 01415 / 01418 / 38100 / 38105 / 03062 / 03063) with the diagnosis heart failure in heart failure patients	Patients with heart failure
	D2-13	V	Rate of heart failure patients prescribed both beta-blockers and ACE inhibitor / AT1 blocker and diuretic at least once in the observation year	Prescription of beta-blocker (ATC C07*) and ACE inhibitor/ AT1 receptor blocker (ATC C09A*, C09B*, C09C* or C09D*) and diuretic (ATC C03*).	Patients with heart failure
	D2-14	V	Rate of heart failure patients prescribed beta-blockers in the observation year	Prescription of beta-blocker (ATC C07*).	Patients with heart failure
	D2-15	V	Rate of heart failure patients prescribed ACE inhibitors or AT1 blockers in the observation year	Prescription of ACE inhibitor/ AT1 receptor blocker (ATC C09A*, C09B*, C09C* or C09D*).	Patients with heart failure
	D2-16	V	Rate of heart failure patients prescribed NSAIDs in the observation year	Prescription of NSAID (ATC M01A* or M01B*).	Patients with heart failure

Group	Ind. No.	Cate gory*	Name of the indicator(s)	Numerator	Denominator
	D2-17	V	Rate of heart failure patients prescribed glitazones or thiazolidinediones in the observation year	Prescription of glitazone or thiazolidindione (ATC A10BG*, A10BD03*, A10BD04*, A10BD05*, A10BD06*, A10BD09* or A10BD12*) .	Patients with heart failure
	D2-18	V	Rate of heart failure Patients prescribed diltiazem or verapamil in the observation year	Prescription of diltiazem or verapamil (ATC C08D*).	Patients with heart failure
	D2-19	V	Rate of heart failure Patients with atrial fibrillation who were prescribed oral anticoagulants in the observation year	Heart failure patients and atrial fibrillation (diagnosis confirmed outpatient I48*) with at least one prescription for vitamin K antagonists (ATC B01AA), factor Xa inhibitors (B01AF), or thrombin inhibitors (B01AE)	Patients with heart failure and atrial fibrillation (ambulatory secured ICD I48)
	D2-20	O	Number of patients with at least one outpatient case in hospital emergency departments during the observation year for patients with heart failure.	Number of heart failure patients for whom an emergency was billed as an outpatient from a hospital (EBM 01205 / 01207 / 01210 / 01212 / 01214 / 01216 / 01218)	Patients with heart failure
	D2-21	O	Proportion of heart failure patients without hospitalization for heart failure within the observation year (risk-adjusted).	Patients without an inpatient hospital case in the observation year due to ICDs (principal diagnosis) of the corresponding disease group	Patients with heart failure
	D2-22	O	Proportion of patients with fewer than two hospitalizations for heart failure within the observation year (risk-adjusted).	Patients with fewer than two inpatient hospitalizations due to ICDs (principal discharge diagnosis) in the disease group	Patients with heart failure
Other diseases of the circulatory system	D3-1	V	Number of jointly treated patients with other diseases of the circulatory system in the network	Number of patients	
	D3-2	V	Number of treated patients with other diseases of the circulatory system	Number of patients	
	D3-3	G	Multimorbidity rate of patients with other diseases of the circulatory system	Patients with more than one disease of the 14 disease groups	Patients with other diseases of the circulatory system
	D3-4	G	Mortality rate of patients with other diseases of the circulatory system within the observation year	Patients who died within the observation period	Patients with other diseases of the circulatory system
	D3-5	V	Rate of patients in the disease group with at least one billing contact with a specialist in general medicine / general practitioner (family doctor) / family internal medicine / internist / specialist internal medicine / cardiology / psychosomatic medicine and psychotherapy / radiology / physical and rehabilitative medicine within the observation year	Patients for whom at least one billing item was billed by a specialist in the respective specialty group	Patients with other diseases of the circulatory system
	D3-6	V	Rate of patients in the disease group with at least one billing contact with two different specialists	Patients for whom at least one billing item was billed by two different specialists	Patients with other diseases of the circulatory system

Group	Ind. No.	Cate gory*	Name of the indicator(s)	Numerator	Denominator
			in the above-mentioned disciplines (each per discipline) within the year of observation	in the respective specialty with different facility identities and with the type of physician utilization "original" in the observation year	
	D3-7	V	Rate of patients in the disease group with at least one billing contact to each of the above specialist groups with referral within the observation year.	Rate of patients with at least one billing contact to each of the above specialist groups with referral within the observation year.	Patients with other diseases of the circulatory system
	D3-8	V	Proportion of patients with billing contact in at least a) one, b) two, c) three d) four of four quarters for above-mentioned physician groups	Proportion of patients with billing contact in at least a) one, b) two, c) three d) four of four quarters for above-mentioned physician groups	Patients with other diseases of the circulatory system
	D3-9	O	Proportion of patients other diseases of the circulatory system without hospitalization for other diseases of the circulatory system within the observation year (risk-adjusted).	Patients without an inpatient hospital case in the observation year due to ICDs (principal diagnosis) of the corresponding disease group	Patients with other diseases of the circulatory system
	D3-10	O	Proportion of patients with fewer than two hospitalizations for other diseases of the circulatory system within the observation year (risk-adjusted).	Patients with fewer than two inpatient hospitalizations due to ICDs (principal discharge diagnosis) in the disease group	Patients with other diseases of the circulatory system
Bronchitis & COPD	D4-1	V	Number of jointly treated patients with bronchitis or COPD in the network	Number of patients	
	D4-2	V	Number of treated patients with bronchitis & COPD	Number of patients	
	D4-3	G	Multimorbidity rate of bronchitis & COPD patients	Patients with more than one disease of the 14 disease groups	Patients with bronchitis & COPD
	D4-4	G	Share of patients enrolled in the disease management program "COPD"	Enrollment in the disease management program	COPD Patients in the bronchitis / COPD group and at least one diagnosis (inpatient or outpatient) J43*, J44* or J47*.
	D4-5	G	Mortality rate of bronchitis & COPD patients within the observation year	Patients who died within the observation period	Patients with bronchitis & COPD
	D4-6	V	Rate of patients in the disease group with at least one billing contact with a specialist in general medicine / general practitioner (family doctor) / family internal medicine / internist / specialist internal medicine / otorhinolaryngology / cardiology / pneumology / radiology / physical and rehabilitative medicine within the observation year	Patients for whom at least one billing item was billed by a specialist in the respective specialty group	Patients with bronchitis or COPD
	D4-7	V	Rate of patients in the disease group with at least one billing contact with two different specialists in the above-mentioned disciplines (each per discipline) within the year of observation	Patients for whom at least one billing item was billed by two different specialists in the respective specialty with different facility identities and with the type of physician utilization	Patients with bronchitis or COPD

Group	Ind. No.	Cate gory*	Name of the indicator(s)	Numerator	Denominator
				"original" in the observation year	
	D4-8	V	Rate of patients in the disease group with at least one billing contact to each of the above specialist groups with referral within the observation year.	Rate of patients with at least one billing contact to each of the above specialist groups with referral within the observation year.	Patients with bronchitis or COPD
	D4-9	V	Rate of COPD patients with at least one billing contact from a pulmonologist	Number of COPD patients for whom at least one billing item was billed by a pulmonology specialist.	COPD Patients in the bronchitis / COPD group and at least one diagnosis (inpatient or outpatient) J43*, J44*, J47*
	D4-10	V	Proportion of patients with billing contact in at least a) one, b) two, c) three d) four of four quarters for above-mentioned physician groups	Proportion of patients with billing contact in at least a) one, b) two, c) three d) four of four quarters for above-mentioned physician groups	Patients with bronchitis or COPD
	D4-11	V	Patients with COPD who participated in a disease management training	COPD patients, for whom a DMP training course was billed during the observation period (EBM region specific)	Patients with COPD enrolled in the disease management program
	D4-12	V	Rate of bronchitis / COPD patients who received an influenza vaccination in the observation year	Patients with bronchitis/COPD as main diagnosis in hospital or confirmed outpatient, who received at least one influenza vaccination (EBM 89111 / 89112 or ATC J07BB) in the observation year	Bronchitis / COPD patients
	D4-13	V	Rate of COPD patients who had at least one pulmonary function test (outpatient) in the observation year	Number of COPD patients for whom a pulmonary function test (EBM 13650 / 03330 (KVNO)) was billed at least once in the year of observation	COPD patients defined by group of bronchitis / COPD and diagnosis (inpatient or outpatient) J43*, J44*, J47*
	D4-14	V	Rate of COPD patients who had an outpatient chest x-ray during the observation year	COPD patients for whom a physician (outpatient) billed for a chest x-ray (EBM 34220) at least once in the observation year	COPD patients defined by group Bronchitis / COPD patients and diagnosis (inpatient or outpatient) J43*, J44*, J47*
	D4-15	V	Rate of COPD patients with at least one home visit with a diagnosis of COPD in the observation year	Home visits with the diagnosis ICD J43*, J44*, J47* for COPD patients (EBM 01410 / 01411 / 01413 / 01415 / 01418 / 38100 / 38105 / 03062 or 03063)	COPD patients defined by group of bronchitis / COPD and at least one diagnosis (inpatient or outpatient) J43*, J44*, J47*
	D4-16	V	Rate of patients with acute bronchitis/bronchiolitis who were prescribed antibiotics in the quarter of the diagnosis	Patients with acute bronchitis/bronchiolitis who were prescribed antibiotics (ATC J01*) in the quarter of diagnosis	Patients with acute bronchitis/bronchiolitis Patients in the bronchitis / COPD group with at least one of the following diagnoses coded as outpatients or inpatients: J20* or J21*
	D4-17	V	Rate of patients with acute bronchitis/bronchiolitis who were prescribed guideline-based antibiotics in the quarter of diagnosis	Patients with acute bronchitis/bronchiolitis who were prescribed guideline-based antibiotics (ATC J01CA / J01CR / J01FA / J01DB / J01DC /	Acute bronchitis/bronchiolitis patients, bronchitis / COPD group, who had at least one diagnosis J20* or

Group	Ind. No.	Cate gory*	Name of the indicator(s)	Numerator	Denominator
				J01DD / J01DE / J01AA or J01MA) in the quarter of diagnosis	J21* coded as outpatients or inpatients
	D4-18	V	Rate of COPD patients who were prescribed beta2mimetics or anticholinergics at least once during the observation year	COPD patients who were prescribed medication of ATC R03A or R03BB during the observation year	COPD patients in the bronchitis / COPD group and diagnosis (inpatient or outpatient) J43*, J44*, J47*
	D4-19	V	Rate of COPD patients who were prescribed inhaled medication (beta2mimetics, anticholinergics, or inhaled glucocorticoids) at least once per quarter during the year of observation	COPD patients who were prescribed inhaled medication (ATC R03A, R03B) at least once in each quarter during the observation year (based on date of prescription)	COPD patients from the group of COPD / bronchitis with the outpatient or inpatient diagnosis J43*, J44*, J47*
	D4-20	O	Number of hospital emergencies with a COPD diagnosis J44* in COPD patients	Number of patients for whom acute exacerbations (ICD J44*) were coded as a confirmed diagnosis and an emergency was billed for the same case number (EBM 01205 / 01207 / 01210 / 01212 / 01214 / 01216 or 01218)	Patients with COPD (discharge diagnosis in hospital or ambulatory diagnosed with confidence "secure" J40.-, J41.-, J42.-, J43.-, J44.-, J47.-)
	D4-21	O	Number of hospital emergencies with COPD patients	Number of COPD patients for whom an emergency was billed in the hospital (EBM 01205 / 01207 / 01210 / 01212 / 01214 / 01216 / 01218)	Patients with COPD (discharge diagnosis in hospital or ambulatory diagnosed with confidence "secure" J40.-, J41.-, J42.-, J43.-, J44.-, J47.-)
	D4-22	O	Proportion of patients with bronchitis or COPD without hospitalization for bronchitis or COPD within the observation year (risk-adjusted)	Patients without an inpatient hospital case in the observation year due to ICDs (principal diagnosis) of the corresponding disease group	Patients with bronchitis or COPD
	D4-23	O	Proportion of patients with fewer than two hospitalizations for bronchitis or COPD within the observation year (risk-adjusted)	Patients with fewer than two inpatient hospitalizations due to ICDs (principal discharge diagnosis) in the disease group	Patients with bronchitis or COPD
	D4-24	O	Proportion of patients with fewer than two hospitalizations with COPD within the observation year (risk-adjusted)	Patients with fewer than two inpatient hospitalizations due to COPD ICDs	Patients with COPD
Mental and behavioral disorders due to use of alcohol or opioids	D5-1	V	Number of jointly treated patients with mental and behavioral disorders due to use of alcohol or opioids in the network	Number of patients	
	D5-2	V	Number of treated patients with mental and behavioral disorders due to use of alcohol or opioids	Number of patients	
	D5-3	G	Multimorbidity rate of patients with mental and behavioral disorders due to use of alcohol or opioids	Patients with more than one disease of the 14 disease groups	Patients with mental and behavioral disorders due to use of alcohol or opioids
	D5-4	G	Mortality rate of patients with mental and behavioral disorders due to use of alcohol or opioids within the observation year	Patients who died within the observation period	Patients with mental and behavioral disorders due to use of alcohol or opioids

Group	Ind. No.	Cate gory*	Name of the indicator(s)	Numerator	Denominator
	D5-5	V	Rate of patients in the disease group with at least one billing contact with a specialist in general medicine / general practitioner (family doctor) / family internal medicine / Internist / internal medicine / otolaryngology / neurology / neurosurgery / psychiatry and psychotherapy / neurology and psychiatry / psychosomatic medicine and psychotherapy / psychotherapeutic physician / psychological psychotherapist within the observation year	Patients for whom at least one billing item was billed by a specialist in the respective specialty group	Patients with mental and behavioral disorders due to use of alcohol or opioids
	D5-6	V	Rate of patients in the disease group with at least one billing contact with two different specialists in the above-mentioned disciplines (each per discipline) within the year of observation	Patients for whom at least one billing item was billed by two different specialists in the respective specialty with different facility identities and with the type of physician utilization "original" in the observation year	Patients with mental and behavioral disorders due to use of alcohol or opioids
	D5-7	V	Rate of patients in the disease group with at least one billing contact to each of the above specialist groups with referral within the observation year.	Rate of patients with at least one billing contact to each of the above specialist groups with referral within the observation year.	Patients with mental and behavioral disorders due to use of alcohol or opioids
	D5-8	V	Proportion of patients with billing contact in at least a) one, b) two, c) three d) four of four quarters for above-mentioned physician groups	Proportion of patients with billing contact in at least a) one, b) two, c) three d) four of four quarters for above-mentioned physician groups	Patients with mental and behavioral disorders due to use of alcohol or opioids
	D5-9	V	Rate of patients with mental and behavioral disorders caused by alcohol and opioids with psychosomatic consultations in the year under observation	Patients with mental and behavioral disorders caused by alcohol and opioids for whom one of the following EBM billing items was billed at least once during the observation year: 35100, 35110, 22221, 22222	Patients with mental and behavioral disorders due to use of alcohol or opioids
	D5-10	O	Proportion of patients with mental and behavioral disorders due to use of alcohol or opioids without hospitalization for mental and behavioral disorders due to use of alcohol or opioids within the observation year (risk-adjusted)	Patients without an inpatient hospital case in the observation year due to ICDs (principal diagnosis) of the corresponding disease group	Patients with mental and behavioral disorders due to use of alcohol or opioids
	D5-11	O	Proportion of patients with fewer than two hospitalizations for mental and behavioral disorders due to use of alcohol or opioids within the observation year (risk-adjusted)	Patients with fewer than two inpatient hospitalizations due to ICDs (principal discharge diagnosis) in the disease group	Patients with mental and behavioral disorders due to use of alcohol or opioids
Back pain [dorsopathies]	D6-1	V	Number of jointly treated patients with back pain [dorsopathies] in the network	Number of patients	
	D6-2	V	Number of treated patients with back pain [dorsopathies]	Number of patients	

Group	Ind. No.	Cate gory*	Name of the indicator(s)	Numerator	Denominator
	D6-3	G	Multimorbidity rate of patients with back pain [dorsopathies]	Patients with more than one disease of the 14 disease groups	Patients with back pain [dorsopathies]
	D6-4	G	Mortality rate of with back pain [dorsopathies] within the observation year	Patients who died within the observation period	Patients with back pain [dorsopathies]
	D6-5	V	Rate of patients in the disease group with at least one billing contact with a specialist in general medicine / general practitioner (family doctor) / family internal medicine / internist / specialist internal medicine / anesthesiology / surgery / rheumatology / neurology / neurosurgery / orthopedics or trauma surgery / psychiatry and psychotherapy / neurology and psychiatry / psychosomatic medicine and psychotherapy / psychotherapeutic physician / psychological psychotherapist / radiology / physical and rehabilitative medicine within the observation year	Patients for whom at least one billing item was billed by a specialist in the respective specialty group	Patients with back pain [dorsopathies]
	D6-6	V	Rate of patients in the disease group with at least one billing contact with two different specialists in the above-mentioned disciplines (each per discipline) within the year of observation	Patients for whom at least one billing item was billed by two different specialists in the respective specialty with different facility identities and with the type of physician utilization "original" in the observation year	Patients with back pain [dorsopathies]
	D6-7	V	Rate of patients in the disease group with at least one billing contact to each of the above specialist groups with referral within the observation year.	Rate of patients with at least one billing contact to each of the above specialist groups with referral within the observation year.	Patients with back pain [dorsopathies]
	D6-8	V	Proportion of patients with billing contact in at least a) one, b) two, c) three d) four of four quarters for above-mentioned physician groups	Proportion of patients with billing contact in at least a) one, b) two, c) three d) four of four quarters for above-mentioned physician groups	Patients with back pain [dorsopathies]
	D6-9	V	Rate of back pain patients who had an X-ray of the spine at least once as an outpatient in the observation year	Back pain patients for whom EBM 34221 or 34222 was billed at least once on an outpatient basis in the observation year	Patients with back pain [dorsopathies]
	D6-10	V	Rate of back pain patients who had an outpatient MRI of the spine at least once during the observation year	Back pain patients for whom EBM 34411 was billed at least once on an outpatient basis in the observation year	Patients with back pain [dorsopathies]
	D6-11	V	Rate of back pain patients who were not prescribed opioids during the observation year	Back pain patients who were not prescribed ATC N02A at least once during the observation year	Patients with back pain [dorsopathies]
	D6-12	O	Proportion of back pain patients without hospitalization for back pain within the observation year (risk-adjusted)	Patients without an inpatient hospital case in the observation year due to ICDs (principal diagnosis) of the	Patients with back pain [dorsopathies]

Group	Ind. No.	Cate gory*	Name of the indicator(s)	Numerator	Denominator
				corresponding disease group	
	D6-13	O	Proportion of patients with fewer than two hospitalizations for back pain within the observation year (risk-adjusted).	Patients with fewer than two inpatient hospitalizations due to ICDs (principal discharge diagnosis) in the disease group	Patients with back pain [dorsopathies]
Hypertension	D7-1	V	Number of jointly treated patients with hypertension in the network	Number of patients	
	D7-2	V	Number of treated patients with hypertension	Number of patients	
	D7-3	G	Multimorbidity rate of patients with hypertension	Patients with more than one disease of the 14 disease groups	Patients with hypertension
	D7-4	G	Mortality rate of patients with hypertension within the observation year	Patients who died within the observation period	Patients with hypertension
	D7-5	V	Rate of patients in the disease group with at least one billing contact with two different specialists in the above-mentioned disciplines (each per discipline) within the year of observation	Patients for whom at least one billing item was billed by two different specialists in the respective specialty with different facility identities and with the type of physician utilization "original" in the observation year	Patients with hypertension
	D7-6	V	Rate of patients in the disease group with at least one billing contact with a specialist in general medicine / general practitioner (family doctor) / family internal medicine / internist / specialist internal medicine / anesthesiology / ophthalmology / cardiology / nephrology within the observation year	Patients for whom at least one billing item was billed by a specialist in the respective specialty group	Patients with hypertension
	D7-7	V	Rate of patients in the disease group with at least one billing contact to each of the above specialist groups with referral within the observation year.	Rate of patients with at least one billing contact to each of the above specialist groups with referral within the observation year.	Patients with hypertension
	D7-8	V	Proportion of patients with billing contact in at least a) one, b) two, c) three d) four of four quarters for above-mentioned physician groups	Proportion of patients with billing contact in at least a) one, b) two, c) three d) four of four quarters for above-mentioned physician groups	Patients with hypertension
	D7-9	V	Rate of hypertension Patients who were prescribed an antihypertensive drug at least once per quarter during the observation year	Patients with hypertension who were prescribed an antihypertensive drug (ATC C02 / C03 / C07 / C08 / C09) at least once in each quarter	Patients with hypertension
	D7-10	V	Rate of hypertension Patients with kidney disease who were prescribed ACE inhibitors or ARB therapy during the observation year.	Hypertensive patients with renal disease (ICD N18* inpatient (principal or secondary diagnosis) or confirmed outpatient) who were prescribed C09A, C09B, C09C, or C09D	Hypertension patients with renal disease (ICD code N18)

Group	Ind. No.	Cate gory*	Name of the indicator(s)	Numerator	Denominator
	D7-11	V	Subgroup: rate of hypertension Patients with advanced kidney disease who received ACE inhibitors or ARB therapy in the observation year	Hypertension Patients with advanced kidney disease (ICD N18.4 or N18.5 inpatient (principal or secondary diagnosis) or confirmed outpatient) who were prescribed ATC C09A / C09B / C09C / C09D	Hypertension patients with advanced kidney disease (ICD codes N18.4, N18.5)
	D7-12	V	Rate of CHD patients with hypertension who were prescribed antihypertensives at least once during the observation year.	CHD patients with hypertension with at least one prescription ATC C02 / C03 / C07 / C08 / C09 in the observation year.	Patients with ischemic heart diseases and hypertension
	D7-13	O	Proportion of hypertension patients without hospitalization for hypertension within the observation year (risk-adjusted)	Patients without an inpatient hospital case in the observation year due to ICDs (principal diagnosis) of the corresponding disease group	Patients with hypertension
	D7-14	O	Proportion of patients with fewer than two hospitalizations for hypertension within the observation year (risk-adjusted)	Patients with fewer than two inpatient hospitalizations due to ICDs (principal discharge diagnosis) in the disease group	Patients with hypertension
Gastroenteritis and other diseases of intestines	D8-1	V	Number of jointly treated patients with gastroenteritis and other diseases of intestines in the network	Number of patients	
	D8-2	V	Number of treated patients with gastroenteritis and other diseases of intestines	Number of patients	
	D8-3	G	Multimorbidity rate of gastroenteritis and other diseases of intestines patients	Patients with more than one disease of the 14 disease groups	Patients with gastroenteritis and other diseases of intestines
	D8-4	G	Mortality rate of patients with gastroenteritis and other diseases of intestines within the observation year	Patients who died within the observation period	Patients with gastroenteritis and other diseases of intestines
	D8-5	V	Rate of patients in the disease group with at least one billing contact with a specialist in general medicine / general practitioner (family doctor) / family internal medicine / internist / specialist internal medicine / gastroenterology within the observation year	Patients for whom at least one billing item was billed by a specialist in the respective specialty group	Patients with gastroenteritis and other diseases of intestines
	D8-6	V	Rate of patients in the disease group with at least one billing contact with two different specialists in the above-mentioned disciplines (each per discipline) within the year of observation	Patients for whom at least one billing item was billed by two different specialists in the respective specialty with different facility identities and with the type of physician utilization "original" in the observation year	Patients with gastroenteritis and other diseases of intestines
	D8-7	V	Rate of patients in the disease group with at least one billing contact to each of the above specialist groups with referral within the observation year.	Rate of patients with at least one billing contact to each of the above specialist groups with referral within the observation year.	Patients with gastroenteritis and other diseases of intestines

Group	Ind. No.	Category*	Name of the indicator(s)	Numerator	Denominator
	D8-8	V	Proportion of patients with billing contact in at least a) one, b) two, c) three d) four of four quarters for above-mentioned physician groups	Proportion of patients with billing contact in at least a) one, b) two, c) three d) four of four quarters for above-mentioned physician groups	Patients with gastroenteritis and other diseases of intestines
	D8-9	V	Rate of patients with gastroenteritis and certain diseases of the intestine who were prescribed opioids and concomitant agents to treat constipation that may have been caused by them	Gastroenteritis and certain diseases of the intestine patients prescribed ATC N02A and A06A in the observation year.	Patients with gastroenteritis and certain diseases of the intestine who were prescribed N02A
	D8-10	V	Rate of patients with diverticulosis/diverticulitis who were prescribed an antibiotic	Rate of patients with diverticulosis/diverticulitis who were prescribed an antibiotic (ATC J01*)	Patients in the disease group with a diagnosis of diverticulosis/diverticulitis
	D8-11	O	Proportion of patients in the disease group with inpatient hospitalization with the diagnosis of diverticulosis / diverticulitis in the observation year	Number of patients with the diagnosis of diverticulosis / diverticulitis in the hospital	Patients with gastroenteritis and other diseases of intestines
	D8-12	O	Proportion of patients with gastroenteritis and other diseases of intestines without hospitalization for gastroenteritis and other diseases of intestines within the observation year (risk-adjusted)	Patients without an inpatient hospital case in the observation year due to ICDs (principal diagnosis) of the corresponding disease group	Patients with gastroenteritis and other diseases of intestines
	D8-13	O	Proportion of patients with fewer than two hospitalizations for gastroenteritis and other diseases of intestines within the observation year (risk-adjusted)	Patients with fewer than two inpatient hospitalizations due to ICDs (principal discharge diagnosis) in the disease group	Patients with gastroenteritis and other diseases of intestines
Intestinal infectious diseases	D9-1	V	Number of jointly treated patients with intestinal infectious diseases in the network	Number of patients	
	D9-2	V	Number of treated patients with intestinal infectious diseases	Number of patients	
	D9-3	G	Multimorbidity rate of patients with intestinal infectious diseases	Patients with more than one disease of the 14 disease groups	Patients with intestinal infectious diseases
	D9-4	G	Mortality rate of patients with intestinal infectious diseases within the observation year	Patients who died within the observation period	Patients with intestinal infectious diseases
	D9-5	V	Rate of patients in the disease group with at least one billing contact with a specialist in general medicine / general practitioner (family doctor) / family internal medicine / internist / specialist internal medicine / gastroenterology / laboratory medicine within the observation year	Patients for whom at least one billing item was billed by a specialist in the respective specialty group	Patients with intestinal infectious diseases
	D9-6	V	Rate of patients in the disease group with at least one billing contact with two different specialists in the above-mentioned disciplines (each per discipline) within the year of observation	Patients for whom at least one billing item was billed by two different specialists in the respective specialty with different facility identities and with the type of physician utilization "original" in the observation year	Patients with intestinal infectious diseases

Group	Ind. No.	Cate gory*	Name of the indicator(s)	Numerator	Denominator
	D9-7	V	Rate of patients in the disease group with at least one billing contact to each of the above specialist groups with referral within the observation year.	Rate of patients with at least one billing contact to each of the above specialist groups with referral within the observation year.	Patients with intestinal infectious diseases
	D9-8	V	Proportion of patients with billing contact in at least a) one, b) two, c) three d) four of four quarters for above-mentioned physician groups	Proportion of patients with billing contact in at least a) one, b) two, c) three d) four of four quarters for above-mentioned physician groups	Patients with intestinal infectious diseases
	D9-9	O	Proportion of patients with intestinal infectious diseases without hospitalization for intestinal infectious diseases within the observation year (risk-adjusted)	Patients without an inpatient hospital case in the observation year due to ICDs (principal diagnosis) of the corresponding disease group	Patients with intestinal infectious diseases
	D9-10	O	Proportion of patients with fewer than two hospitalizations for intestinal infectious diseases within the observation year (risk-adjusted)	Patients with fewer than two inpatient hospitalizations due to ICDs (principal discharge diagnosis) in the disease group	Patients with intestinal infectious diseases
Influenza and pneumonia	D10-1	V	Number of jointly treated patients with influenza and pneumonia in the network	Number of patients	
	D10-2	V	Number of treated patients with influenza and pneumonia	Number of patients	
	D10-3	G	Multimorbidity rate of patients with influenza and pneumonia	Patients with more than one disease of the 14 disease groups	Patients with influenza and pneumonia
	D10-4	G	Mortality rate of patients with influenza and pneumonia within the observation year	Patients who died within the observation period	Patients with influenza and pneumonia
	D10-5	V	Rate of patients in the disease group with at least one billing contact with a specialist in general medicine / general practitioner (family doctor) / family internal medicine / internist / specialist internal medicine / otorhinolaryngology / pneumology / radiology within the observation year	Patients for whom at least one billing item was billed by a specialist in the respective specialty group	Patients with influenza and pneumonia
	D10-6	V	Rate of patients in the disease group with at least one billing contact with two different specialists in the above-mentioned disciplines (each per discipline) within the year of observation	Patients for whom at least one billing item was billed by two different specialists in the respective specialty with different facility identities and with the type of physician utilization "original" in the observation year	Patients with influenza and pneumonia
	D10-7	V	Rate of patients in the disease group with at least one billing contact to each of the above specialist groups with referral within the observation year.	Rate of patients with at least one billing contact to each of the above specialist groups with referral within the observation year.	Patients with influenza and pneumonia

Group	Ind. No.	Cate gory*	Name of the indicator(s)	Numerator	Denominator
	D10-8	V	Proportion of patients with billing contact in at least a) one, b) two, c) three d) four of four quarters for above-mentioned physician groups	Proportion of patients with billing contact in at least a) one, b) two, c) three d) four of four quarters for above-mentioned physician groups	Patients with influenza and pneumonia
	D10-9	V	Rate of influenza and pneumonia patients who consulted a primary care physician after an inpatient hospitalization for pneumonia in the same or subsequent quarter	Patients who were coded ICD J12* / J13* / J14* / J15* as principal inpatient diagnosis and for whom any EBM billing item was billed by a primary care physician (general practitioner (family physician), physician/practitioner (family physician), internist (family physician)) in the same or subsequent quarter	Influenza and pneumonia patients who were coded ICD J12* / J13* / J14* / J15* as main inpatient diagnosis. Only patients with index event in the 1st to 3rd quarter considered.
	D10-10	V	Rate of influenza/pneumonia patients with at least one home visit diagnosed with influenza/pneumonia in the observation year	Number of influenza/pneumonia patients with at least one home visit diagnosed as influenza and pneumonia patients (EBM 01410 / 01411 / 01413 / 01415 / 01418 / 38100 / 38105 / 03062 / 03063)	Influenza and pneumonia patients
	D10-11	V	Rate of patients with (viral or bacterial) pneumonia who were prescribed an antibiotic in the observation year	Pneumonia Patients who were prescribed an antibiotic (ATC J01) at least once during the observation year	Patients with viral or bacterial pneumonia defined as patients in the influenza and pneumonia group and at least one of the following diagnoses outpatient (confirmed) or inpatient (principal diagnosis): J12*, J13*, J14*, J15*
	D10-12	V	Rate of patients with pneumonia (viral or bacterial) who were prescribed one of the antibiotics recommended for use in pneumonia during the year of observation	Pneumonia Patients who were prescribed one of the antibiotics recommended for use in pneumonia (ATC J01CR / J01FA / J01DB / J01DC / J01DD / J01DE / J01DH / J01AA / J01MA or J01G) at least once during the observation year	Patient with viral or bacterial pneumonia. Patient is part of influenza and pneumonia patients and has received at least one of the following diagnoses outpatient (confirmed) or inpatient (principal diagnosis): J12*, J13*, J14*, J15*
	D10-13	O	Proportion of patients with influenza and pneumonia without hospitalization for influenza and pneumonia within the observation year (risk-adjusted)	Patients without an inpatient hospital case in the observation year due to ICDs (principal diagnosis) of the corresponding disease group	Patients with influenza and pneumonia
	D10-14	O	Proportion of patients with fewer than two hospitalizations for influenza and pneumonia within the observation year (risk-adjusted)	Patients with fewer than two inpatient hospitalizations due to ICDs (principal discharge diagnosis) in the disease group	Patients with influenza and pneumonia

Group	Ind. No.	Cate gory*	Name of the indicator(s)	Numerator	Denominator
Ear nose throat infections	D11-1	V	Number of jointly treated patients with ear nose throat infections in the network	Number of patients	
	D11-2	V	Number of treated patients with ear nose throat infections	Number of patients	
	D11-3	G	Multimorbidity rate of patients with ear nose throat infections	Patients with more than one disease of the 14 disease groups	Patients with ear nose throat infections
	D11-4	G	Mortality rate of patients with ear nose throat infections within the observation year	Patients who died within the observation period	Patients with ear nose throat infections
	D11-5	V	Rate of patients in the disease group with at least one billing contact with a specialist in general medicine / general practitioner (family doctor) / family internal medicine / otorhinolaryngology / pneumology / radiology within the observation year	Patients for whom at least one billing item was billed by a specialist in the respective specialty group	Patients with ear nose throat infections
	D11-6	V	Rate of patients in the disease group with at least one billing contact with two different specialists in the above-mentioned disciplines (each per discipline) within the year of observation	Patients for whom at least one billing item was billed by two different specialists in the respective specialty with different facility identities and with the type of physician utilization "original" in the observation year	Patients with ear nose throat infections
	D11-7	V	Rate of patients in the disease group with at least one billing contact to each of the above specialist groups with referral within the observation year.	Rate of patients with at least one billing contact to each of the above specialist groups with referral within the observation year.	Patients with ear nose throat infections
	D11-8	V	Proportion of patients with billing contact in at least a) one, b) two, c) three d) four of four quarters for above-mentioned physician groups	Proportion of patients with billing contact in at least a) one, b) two, c) three d) four of four quarters for above-mentioned physician groups	Patients with ear nose throat infections
	D11-9	V	Rate of sinusitis patients who were prescribed an antibiotic in the observation year	Sinusitis patients who were prescribed an antibiotic (ATC J01) in the observation year	Sinusitis patients defined as patients in the group with ENT infections and at least one of the following diagnoses inpatient (confirmed) or outpatient (principal diagnosis) J32 or J01
	D11-10	V	Rate of ENT infections patients with otitis media or myringitis who were prescribed an antibiotic in the observation year	Rate of influenza and pneumonia patients with otitis media or myringitis who were prescribed an antibiotic (ATC J01) during the observation year	Flu and pneumonia group patients with otitis media or myringitis with at least one diagnosis inpatient (principal diagnosis) or outpatient (confirmed) H65*/ H66*/ H73*/ H73*/ H67* / H68* / H69* / H70*
	D11-11	V	Rate of ENT infections Patients with otitis media or myringitis who were prescribed recommended antibiotics (amoxicillin (penicillins), sulfonamide/trimethoprim, or phenoxymethyl penicillins) during the observation year	Rate of influenza and pneumonia patients with otitis media or myringitis who were prescribed amoxicillin (penicillins) (ATC J01CA / J01E / J01CE) in the observation year. This does not include quinolones (ATC J01M)	Influenza and pneumonia patients with otitis media or myringitis with at least one diagnosis inpatient (principal diagnosis) or outpatient (confirmed) H65*/ H66*/ H73*/ H73*/ H67* / H68* / H69* / H70*

Group	Ind. No.	Cate gory*	Name of the indicator(s)	Numerator	Denominator
	D11-12	V	Rate of pharyngitis patients who were prescribed recommended antibiotics (phenoxymethylpenicillin or erythromycin)	Phrayngitis patients who were prescribed phenoxymethylpenicillin or erythromycin (ATC J01CE02 or J01FA01)	Pharyngitis patients defined as patients in the group with ENT infections and at least one of the following diagnoses outpatient (confirmed) or inpatient (principal diagnosis) R07.0, J03, J02, J06.0, J35
	D11-13	V	Rate of pharyngitis patients who were prescribed antibiotics other than recommended phenoxymethylpenicillin or erythromycin	Pharyngitis patients who were prescribed ATC J01A / J01D / J01E / J01F / J01M / J01X but not J01CE02 / J01FA01	Pharyngitis patients defined as patients with at least one of the following diagnoses outpatient (confirmed) or inpatient (principal diagnosis) R07.0, J03, J02, J06.0, J35
	D11-13	O	Proportion of patients with ear nose throat infections without hospitalization for ear nose throat infections within the observation year (risk-adjusted)	Patients without an inpatient hospital case in the observation year due to ICDs (principal diagnosis) of the corresponding disease group	Patients with ear nose throat infections
	D11-15	O	Proportion of patients with fewer than two hospitalizations for ear nose throat infections within the observation year (risk-adjusted)	Patients with fewer than two inpatient hospitalizations due to ICDs (principal discharge diagnosis) in the disease group	Patients with ear nose throat infections
Depressive disorders	D12-1	V	Number of jointly treated patients with depressive disorders in the network	Number of patients	
	D12-2	V	Number of treated patients with depressive disorders	Number of patients	
	D12-3	V	Multimorbidity rate of patients with depressive disorders	Patients with more than one disease of the 14 disease groups	Patients with depressive disorders
	D12-4	G	Mortality rate of patients with depressive disorders within the observation year	Patients who died within the observation period	Patients with depressive disorders
	D12-5	G	Rate of patients in the disease group with at least one billing contact with a specialist in general medicine / general practitioner (family doctor) / family internal medicine / gynecology / neurology / neurosurgery psychiatry and psychotherapy / neurology and psychiatry / psychosomatic medicine and psychotherapy / psychotherapeutic physician / psychological psychotherapist within the observation year	Patients for whom at least one billing item was billed by a specialist in the respective specialty group	Patients with depressive disorders
	D12-6	V	Rate of patients in the disease group with at least one billing contact with two different specialists in the above-mentioned disciplines (each per discipline) within the year of observation	Patients for whom at least one billing item was billed by two different specialists in the respective specialty with different facility identities and with the type of physician utilization "original" in the observation year	Patients with depressive disorders

Group	Ind. No.	Cate gory*	Name of the indicator(s)	Numerator	Denominator
	D12-7	V	Rate of patients in the disease group with at least one billing contact to each of the above specialist groups with referral within the observation year.	Rate of patients with at least one billing contact to each of the above specialist groups with referral within the observation year.	Patients with depressive disorders
	D12-8	V	Proportion of patients with billing contact in at least a) one, b) two, c) three d) four of four quarters for above-mentioned physician groups	Proportion of patients with billing contact in at least a) one, b) two, c) three d) four of four quarters for above-mentioned physician groups	Patients with severe depressive episode (hospital discharge diagnosis or ambulatory diagnosed with confidence "secure" (F32.2.-, F32.3.-, F33.2.- or F33.3.-) within the first 9 months of the observation year.
	D12-9	V	Rate of patients with depressive disorders with a psychosomatic consultation at least once during the observation year	Patients with depressive Disorders for whom one of the following billing items was billed at least once during the observation year: EBM 35100 / 35110 / 22221 / 22222	Patients with depressive disorders
	D12-10	V	Rate of patients with depressive disorders who were prescribed anxiolytics, hypnotics or sedatives in the observation year	Patients with depressive disorders who were prescribed anxiolytics, hypnotics, or sedatives (ATC N05B or N05C) at least once during the year of observation.	Patients with depressive disorders
	D12-11	V	Rate of patients with depressive disorders who were prescribed antidepressants and at the same time anxiolytics, hypnotics, or sedatives during the observation year	Patients with depressive disorders who were prescribed antidepressants (ATC N06A) and at the same time anxiolytics, hypnotics, or sedatives (ATC N05B or N05C) at least once during the observation year	Patients with depressive disorders who were prescribed antidepressants (ATC code N06A)
	D12-12	V	Rate of patients with depressive disorders with at least a moderate episode who were prescribed an antidepressant during the observation year	Patients with depressive disorders with at least moderate depression who were prescribed an antidepressant (ATC N06a)	Patients with Depressive Disorders with at least moderate depression defined from the group of patients with Depressive Disorders and outpatient (confirmed) or inpatient (principal diagnosis) at least one of the following diagnoses: ICD F32.1 / F32.2 / F32.3 / F33.1 / F33.2 / F33.3 / F33.4
	D12-13	V	Rate of patients with depressive disorders with at least a moderate episode who received another prescription for an antidepressant in the observation year after receiving an antidepressant within the same or the following quarter	Patients with depressive disorders with at least a moderate episode who, after receiving an antidepressant for the first time (ATC N06A), received another prescription for an antidepressant within the same or subsequent quarter	Patients with Depressive Disorders with at least moderate depression defined as either secured outpatient or inpatient (principal diagnosis) at least one of the following diagnoses: F32.1 / F32.2 / F32.3 / F33.1 / F33.2 / F33.3 / F33.4. Here, the denominator is narrowed to patients who received a prescription in the first, second, or third quarter

Group	Ind. No.	Cate gory*	Name of the indicator(s)	Numerator	Denominator
	D12-14	O	Proportion of patients with depressive disorders without hospitalization for depressive disorders within the observation year (risk-adjusted)	Patients without an inpatient hospital case in the observation year due to ICDs (principal diagnosis) of the corresponding disease group	Patients with depressive disorders
	D12-15	O	Proportion of patients with fewer than two hospitalizations for depressive disorders within the observation year (risk-adjusted)	Patients with fewer than two inpatient hospitalizations due to ICDs (principal discharge diagnosis) in the disease group	Patients with depressive disorders
Diabetes mellitus	D13-1	V	Number of jointly treated patients with diabetes type 1 in the network	Number of patients	
	D13-2	V	Number of jointly treated patients with diabetes type 2 in the network	Number of patients	
	D13-3	V	Number of treated patients with diabetes mellitus, type 1	Number of patients	
	D13-4	V	Number of treated patients with diabetes mellitus, type 2	Number of patients	
	D13-5	G	Multimorbidity rate of diabetes mellitus, type 2 patients	Patients with more than one disease of the 14 disease groups	Patients with diabetes mellitus, type 2
	D13-6	G	Multimorbidity rate of diabetes mellitus, type 1 patients	Patients with more than one disease of the 14 disease groups	Patients with diabetes mellitus, type 1
	D13-7	G	Share of patients enrolled in the disease management program "diabetes mellitus, type 1"	Enrollment in the disease management program	Patients with diabetes mellitus, type 1
	D13-8	G	Share of patients enrolled in the disease management program "diabetes mellitus, type 2"	Enrollment in the disease management program	Patients with diabetes mellitus, type 2
	D13-9	G	Mortality rate of patients with diabetes mellitus, type 1 within the observation year	Patients who died within the observation period	Patients with diabetes mellitus, type 1
	D13-10	G	Mortality rate of patients with diabetes mellitus, type 2 within the observation year	Patients who died within the observation period	Patients with diabetes mellitus, type 2
	D13-11	V	Rate of patients in the disease group with at least one billing contact with a specialist in general medicine / general practitioner (family doctor) / family internal medicine / internist / specialist internal medicine / nephrology / ophthalmology / diabetic focus practice within the observation year	Patients for whom at least one billing item was billed by a specialist in the respective specialty group	Patients with diabetes mellitus, type 1
	D13-12	V	Rate of patients in the disease group with at least one billing contact with a specialist in general medicine / general practitioner (family doctor) / family internal medicine / internist / specialist internal medicine / nephrology / ophthalmology / diabetic focus practice within the observation year	Patients for whom at least one billing item was billed by a specialist in the respective specialty group	Patients with diabetes mellitus, type 2
	D13-13	V	Rate of patients in the disease group with at least one billing contact with two different specialists in the above-mentioned disciplines (each per discipline) within the year of observation	Patients for whom at least one billing item was billed by two different specialists in the respective specialty with different facility identities and with the type of physician utilization	Patients with diabetes mellitus, type 1

Group	Ind. No.	Cate gory*	Name of the indicator(s)	Numerator	Denominator
				"original" in the observation year	
	D13-14	V	Rate of patients in the disease group with at least one billing contact with two different specialists in the above-mentioned disciplines (each per discipline) within the year of observation	Patients for whom at least one billing item was billed by two different specialists in the respective specialty with different facility identities and with the type of physician utilization "original" in the observation year	Patients with diabetes mellitus, type 2
	D13-15	V	Rate of patients with type 1 diabetes who had outpatient contact with a primary care physician or internist at least twice during the year of observation.	Type 1 diabetes patients who have been billed at least twice by a specialist in the following specialist groups: general practitioner (family physician), physician/practitioner (family physician), internist (family physician), internist).	Patients with diabetes mellitus, type 1
	D13-16	V	Rate of patients with type 2 diabetes who had outpatient contact with a primary care physician or internist at least twice during the year of observation.	Type 2 diabetes patients who have been billed at least twice by a specialist in the following specialist groups: general practitioner (family physician), physician/practitioner (family physician), internist (family physician), internist).	Patients with diabetes mellitus, type 2
	D13-17	V	Rate of patients in the disease group with at least one billing contact to each of the above specialist groups with referral within the observation year.	Rate of patients with at least one billing contact to each of the above specialist groups with referral within the observation year.	Patients with diabetes mellitus, type 1
	D13-18	V	Rate of patients in the disease group with at least one billing contact to each of the above specialist groups with referral within the observation year.	Rate of patients with at least one billing contact to each of the above specialist groups with referral within the observation year.	Patients with diabetes mellitus, type 2
	D13-19	V	Proportion of patients with billing contact in at least a) one, b) two, c) three d) four of four quarters for above-mentioned physician groups	Proportion of patients with billing contact in at least a) one, b) two, c) three d) four of four quarters for above-mentioned physician groups	Patients with diabetes mellitus, type 1
	D13-20	V	Proportion of patients with billing contact in at least a) one, b) two, c) three d) four of four quarters for above-mentioned physician groups	Proportion of patients with billing contact in at least a) one, b) two, c) three d) four of four quarters for above-mentioned physician groups	Patients with diabetes mellitus, type 2
	D13-21	V	Rate of diabetes type 1 patients who participated in DMP training during the observation year	Diabetes patients type I, for whom a training course was billed (EBM region specific)	Patients with diabetes mellitus type 1 enrolled in the disease management program
	D13-22	V	Rate of diabetes type 2 patients who participated in DMP training in the observation year	Type 2 diabetes patients for whom training was billed (EBM region specific)	Patients with diabetes mellitus type 2 enrolled in the disease management program

Group	Ind. No.	Cate gory*	Name of the indicator(s)	Numerator	Denominator
	D13-23	V	Rate of patients diagnosed with diabetes type 1 who received an influenza vaccination in the observation year	Type 1 diabetes patients who received at least one influenza vaccination (EBM 89111 / 89112 or ATC J07BB) during the observation year	Patients with diabetes mellitus, type 1
	D13-24	V	Rate of patients diagnosed with diabetes type 2 who received an influenza vaccination in the observation year	Type 2 diabetes patients who received at least one influenza vaccination (EBM 89111 / 89112 or ATC J07BB) during the observation year	Patients with diabetes mellitus, type 2
	D13-25	V	Rate of type 1 diabetes patients who underwent ocular fundus examination in the observation year	Diabetes patients type 1, for whom an ocular fundus examination EBM 06333 (and regional reimbursement item) was billed at least once as an outpatient in the observation year.	Patients with diabetes mellitus, type 1
	D13-26	V	Rate of type 2 diabetes patients who underwent ocular fundus examination in the observation year	Diabetes patients type 2, for whom an outpatient ocular fundus examination 06333 (as well regional reimbursement items) was billed at least once during the observation year.	Patients with diabetes mellitus, type 2
	D13-27	V	Rate of type 1 diabetes patients with at least one HDL test in the observation year.	Type 1 diabetes patients who were billed for at least one HDL (EBM 32061) in the observation year	Patients with diabetes mellitus, type 1
	D13-28	V	Rate of type 2 diabetes patients with at least one HDL test in the observation year.	Type 2 diabetes patients who were billed for at least one HDL (EBM 32061) in the year of observation	Patients with diabetes mellitus, type 2
	D13-29	V	Rate of diabetes mellitus type 1 patients with an Hba1c determination as outpatients in the observation year	Diabetes patients type 1, for whom an Hba1c determination was billed at least once on an outpatient basis (EBM 32094 or regional reimbursement items)	Patients with diabetes mellitus, type 1
	D13-30	V	Rate of diabetes mellitus type 2 patients with an Hba1c determination as outpatients in the observation year	Type 2 diabetes patients for whom an Hba1c determination was billed at least once on an outpatient basis (EBM 32094 / 90310 (KVNO) / 90321 (KVNO))	Patients with diabetes mellitus, type 2
	D13-31	V	Rate of diabetes type 1 patients with at least two HbA1c determinations and at least one LDL test in the observation year	Diabetes type 1 patients who were billed at least twice for an HbA1c test (EBM 32094 or regional reimbursement items) and at least once for an LDL test (EBM 32062) during the observation year.	Patients with diabetes mellitus, type 1
	D13-32	V	Rate of diabetes type 2 patients with at least two HbA1c determinations and at least one LDL test in the observation year	Diabetes type 2 patients who were billed at least twice for an HbA1c test (EBM 32094 / regional reimbursement item) and at least one LDL test (EBM 32062) in the observation year.	Patients with diabetes mellitus, type 2

Group	Ind. No.	Cate gory*	Name of the indicator(s)	Numerator	Denominator
	D13-33	V	Rate of type 1 diabetes patients with prescriptions for oral antidiabetic drugs in the year of observation	Diabetes patients type 1 with prescription of oral antidiabetic drugs (ATC A10B) during the observation period	Patients with diabetes mellitus, type 1
	D13-34	V	Proportion of CHD patients with type II diabetes who were prescribed lipid-lowering agents in the year of observation.	CHD patients who are also diabetes type 2 patients and were prescribed lipid-lowering agents (ATC C10A / C10B) at least once during the observation year	CHD patients with at least 2 confirmed outpatient or one inpatient E11.* diagnosis/s
	D13-35	V	Rate of type 2 diabetes patients with prescriptions for oral antidiabetic drugs in the year of observation	Diabetes patients type 2 with prescription of oral antidiabetic drugs (ATC A10B) during the observation period	Patients with diabetes mellitus, type 2
	D13-36	V	Rate of diabetes type II patients with metformin prescription in observation year	Type 2 diabetes patients prescribed metformin (ATC A10BA02 or A10BD*) in the observation year	Patients with diabetes mellitus, type 2
	D13-37	V	Rate of diabetes type 1 patients with prescription of insulin or analogues in the observation year	Type 1 patients with prescription of insulin or analogs (ATC A10A* and / or A10B*) during the observation period.	Patients with diabetes mellitus, type 1
	D13-38	V	Rate of diabetes type 2 patients with prescription of insulin or analogues in the observation year	Type 2 patients with prescription of insulin or analogs (ATC A10A* and / or A10B*) during the observation period.	Patients with diabetes mellitus, type 2
	D13-39	O	Rate of diabetes type 1 patients with at least one myocardial infarction within the observation year	Rate of diabetes type I patients with at least one myocardial infarction	Patients with diabetes mellitus, type 1
	D13-40	O	Rate of diabetes type 2 patients with at least one myocardial infarction within the observation year	Rate of diabetes type II patients with at least one myocardial infarction	Patients with diabetes mellitus, type 2
	D13-41	O	Proportion of patients with diabetes mellitus, type 1 without hospitalization for diabetes mellitus, type 1 within the observation year (risk-adjusted)	Patients without an inpatient hospital case in the observation year due to ICDs (principal diagnosis) of the corresponding disease group	Patients with diabetes mellitus, type 1
	D13-42	O	Proportion of patients with fewer than two hospitalizations for diabetes mellitus, type 2 within the observation year (risk-adjusted)	Patients with fewer than two inpatient hospitalizations due to ICDs (principal discharge diagnosis) in the disease group	Patients with diabetes mellitus, type 2
	D13-43	O	Proportion of patients with fewer than two hospitalizations for diabetes mellitus, type 1 within the observation year (risk-adjusted)	Patients without an inpatient hospital case in the observation year due to ICDs (principal diagnosis) of the corresponding disease group	Patients with diabetes mellitus, type 1
	D13-44	O	Proportion of patients with diabetes mellitus, type 2 without hospitalization for diabetes mellitus, type 2 within the observation year (risk-adjusted)	Patients with fewer than two inpatient hospitalizations due to ICDs (principal discharge diagnosis) in the disease group	Patients with diabetes mellitus, type 2

Group	Ind. No.	Cate gory*	Name of the indicator(s)	Numerator	Denominator
Gonarthrosis [arthrosis of knee]	D14-1	V	Number of jointly treated patients with gonarthrosis [arthrosis of knee] in the network	Number of patients	
	D14-2	V	Number of treated patients with gonarthrosis [arthrosis of knee]	Number of patients	
	D14-3	G	Multimorbidity rate of patients with gonarthrosis [arthrosis of knee]	Patients with more than one disease of the 14 disease groups	Patients with gonarthrosis [arthrosis of knee]
	D14-4	G	Mortality rate of patients gonarthrosis [arthrosis of knee] within the observation year	Patients who died within the observation period	Patients with gonarthrosis [arthrosis of knee]
	D14-5	V	Rate of patients in the disease group with at least one billing contact with a specialist in general medicine / general practitioner (family doctor) / family internal medicine / anesthesiology / surgery / orthopedic or trauma surgeons / rheumatology / psychosomatic Medicine and Psychotherapy / Radiology / Physical and Rehabilitation Medicine within the observation year	Patients for whom at least one billing item was billed by a specialist in the respective specialty group	Patients with gonarthrosis [arthrosis of knee]
	D14-6	V	Rate of patients in the disease group with at least one billing contact with two different specialists in the above-mentioned disciplines (each per discipline) within the year of observation	Patients for whom at least one billing item was billed by two different specialists in the respective specialty with different facility identities and with the type of physician utilization "original" in the observation year	Patients with gonarthrosis [arthrosis of knee]
	D14-7	V	Rate of patients in the disease group with at least one billing contact to each of the above specialist groups with referral within the observation year.	Rate of patients with at least one billing contact to each of the above specialist groups with referral within the observation year.	Patients with gonarthrosis [arthrosis of knee]
	D14-8	V	Proportion of patients with billing contact in at least a) one, b) two, c) three d) four of four quarters for above-mentioned physician groups	Proportion of patients with billing contact in at least a) one, b) two, c) three d) four of four quarters for above-mentioned physician groups	Patients with gonarthrosis [arthrosis of knee]
	D14-9	V	Rate of gonarthrosis patients who were not prescribed opioids within the observation year.	Gonarthrosis patients who were not prescribed ATC N02A at least once during the observation year	Patients with gonarthrosis [arthrosis of knee]
	D14-10	O	Proportion of with gonarthrosis patients without hospitalization for gonarthrosis within the observation year (risk-adjusted).	Patients without an inpatient hospital case in the observation year due to ICDs (principal diagnosis) of the corresponding disease group	Patients with gonarthrosis [arthrosis of knee]
	D14-11	V	Number of patients with gonarthrosis who received knee arthroplasty during the observation period	Number of patients with gonarthrosis who received knee arthroplasty	Patients with gonarthrosis [arthrosis of knee]

Group	Ind. No.	Cate gory*	Name of the indicator(s)	Numerator	Denominator
	D14-12	O	Thrombosis or pulmonary embolism within 6 months after a knee endoprothesis	Patients with a thrombosis or pulmonary embolism within the same or the following two quarters of the implementation (hospital discharge diagnosis or ambulatory diagnosed with confidence "secure" I80.1.-, I80.2.-, I80.3.-, 182.2.- or I26.-).	Gonarthrosis patients with implementation of a knee endoprothesis (OPS 5-822*) within the two first quarters of the observation year.
	D14-13	O	Proportion of patients with fewer than two hospitalizations for gonarthrosis within the observation year (risk-adjusted).	Patients with fewer than two inpatient hospitalizations due to ICDs (principal discharge diagnosis) in the disease group	Patients with gonarthrosis [arthrosis of knee]

* Indicator categories: G- patient characteristics, V – information on patient pathways, O – outcome indicators

A4 –Table of Indicators [German]

Nr.	Kat.*	Indikatorbeschreibung	Zähler	Nenner
**00 Indikationsübergreifende Indikatoren**				
G1	V	Anzahl unterschiedlicher Patient:innen pro Netzwerk	Anzahl gemeinsam behandelter Patient:innen mit mindestens einer Krankheitsgruppe im Netzwerk	
G2	V	Anzahl durchschnittlich geteilter Patient:innen pro Netzwerk	Anzahl durchschnittlich geteilter Patient:innen pro Netzwerk	
G3	V	Größe des Netzwerks: Anzahl an Ärzt:innen im Netz	Anzahl an Ärzt:innen im Netzwerk	
G4	V	Netzwerktreue - Wie viele Ärzt:innen werden von den Netzwerkpatient:innen innerhalb des Netzes in Relation zu denen außerhalb des Netzes aufgesucht	Netzwerkpatient:innen, die durch Ärzt:innen innerhalb des Netzwerks behandelt wurden	Netzwerkpatient:innen, die durch Ärzt:innen außerhalb des Netzwerks behandelt wurden
G5	G	Anteil männlicher / weiblicher Netzwerkpatient:innen	Anzahl männliche / weibliche Netzwerkpatient:innen	Anzahl Netzwerkpatient:innen
G6	G	Anzahl der Patient:innen in Altersgruppen (<45, 45-54, 55-64, 65-74, 75-84, >85)	Anzahl der Patient:innen in der jeweiligen Altersgruppe	Anzahl der Patient:innen mit mindestens einer Krankheitsgruppe
G7	V	Rate der Patient:innen älter als 65 Jahre, die im Beobachtungsjahr eine Grippeimpfung erhielten	Patient:innen älter als 65 Jahre mit den ACD Krankheitsgruppen, die im Beobachtungsjahr mindestens eine Grippeimpfung (EBM 89111 / 89112 oder ATC J07BB) erhielten	Patient:innen über 65 Jahr mit den ACD Krankheitsgruppen
**01 Ischämische Herzkrankheiten**				
D1-1	V	Patient:innen mit Ischämischen Herzkrankheiten: Anteil der Patient:innen im Verhältnis zu den Netzwerkpatient:innen	KHK-Patient:innen	Netzwerkpatient:innen
D1-2	V	Ischämische Herzkrankheiten: Anzahl der gemeinsam behandelten Patient:innen mit mindestens einer der Diagnosen im Netzwerk	Anzahl der behandelten Patient:innen mit mindestens einer der relevanten Diagnosen	
D1-3	G	Anteil der multimorbiden Patient:innen: Ischämische Herzkrankheiten	Patient:innen der Krankheitsgruppe, die mindestens zwei verschiedene Krankheitsgruppen im Beobachtungsjahr kodiert bekamen	KHK Patient:innen
D1-4	V	Rate der KHK Patient:innen, die im Beobachtungsjahr im KHK-DMP eingeschrieben waren	KHK Patient:innen, die am KHK-DMP teilnehmen (Einschreibung über den gesamten Beobachtungszeitraum)	KHK Patient:innen
D1-5	G	Mortalitätsrate der KHK Patient:innen innerhalb des Beobachtungsjahrs	Patient:innen der Krankheitsgruppe, die im Beobachtungsjahr verstarben	KHK Patient:innen
D1-6	V	Rate der KHK Patient:innen mit mindestens einem Abrechnungskontakt zu einem/einer Fachärzt:in für (1) Allgemeinmedizin / Praktische/r Ärzt:in (Hausärzt:in) / hausärztliche Innere Medizin (2) Internistin / fachärztliche Innere Medizin (3) Nuklearmedizin (4) Psychosomatische Medizin und Psychotherapie (5) Physikalische und Rehabilitative Medizin (6) Kardiologie innerhalb des Beobachtungsjahrs	Patient:innen der Krankheitsgruppe, für die mindestens eine Abrechnungsposition von einem / einer Fachärzt:in für (1) – (6) abgerechnet wurde	KHK Patient:innen
D1-7	V	Rate der KHK Patient:innen mit mindestens einem Abrechnungskontakt zu (2) – (6) mit Überweisung innerhalb des Beobachtungsjahrs	KHK Patient:innen, für die mindestens eine Abrechnungsposition von einem/r (2) – (6) mit Überweisung abgerechnet wurde	KHK Patient:innen

Nr.	Kat.*	Indikatorbeschreibung	Zähler	Nenner
D1-8	V	Anteil der Patient:innen mit Abrechnungskontakt in mindestens einem von vier Quartalen bei oben genannten Arztgruppen	Patient:innen mit Abrechnungskontakt in mindestens einem von vier Quartalen	Patient:innen der Krankheitsgruppe
D1-9	V	Rate der KHK Patient:innen mit mindestens einem Abrechnungskontakt zu zwei verschiedenen Fachärzt:innen (1) – (6) innerhalb des Beobachtungsjahrs	KHK Patient:innen, für die mindestens eine Abrechnungsposition von zwei Fachärzt:innen für (1) – (6) unterschiedlicher BSNR und mit der Art der ärztlichen Inanspruchnahme "O" abgerechnet wurde	KHK Patient:innen
D1-10	V	Rate der KHK Patient:innen mit Grippeimpfung im Beobachtungsjahr	KHK Patient:innen, für die mindestens einmal ambulant eine Grippeimpfung abgerechnet wurde (EBM 89111 / 89112) oder die mindestens einmal ATC J07BB verordnet bekamen	KHK Patient:innen
D1-11	V	Rate der KHK Patient:innen, die im Beobachtungsjahr an einer DMP Schulung teilnahmen	KHK Patient:innen, für die eine Schulung abgerechnet wurde (EBM 90283 (KV WL) / 90285 (KV WL) / 90287 (KV WL) / 90513 (KV NO) / 90514 (KV NO) / 90515 (KV NO) / 90517 (KV NO) / 99436 (KV HH) /99786H/J/L (KV SH) / 99786N (KV SH) / 997860 (KV SH))	KHK Patient:innen mit DMP Einschreibung im Beobachtungsjahr
D1-12	V	Rate der KHK Patient:innen, deren LDL und HDL Werte mindestens einmal im Beobachtungsjahr ambulant untersucht wurden	KHK Patient:innen, für die mindestens einmal im Beobachtungsjahr ambulant EBM 32060 Gesamtcholesterin oder 32061 und 32062 abgerechnet wurde	KHK Patient:innen
D1-13	V	Rate der KHK Patient:innen, die im Beobachtungsjahr mindestens einmal pro Quartal Statine verordnet bekamen	KHK Patient:innen, die mindestens einmal in jedem Quartal (Verordnungsdatum) während des Beobachtungsjahres ATC C10AA, C10BA oder C10BX verordnet bekamen	KHK Patient:innen
D1-14	V	Rate der KHK Patient:innen, die im Beobachtungsjahr mindestens einmal Statine verschrieben bekamen	KHK Patient:innen mit mindestens einer Verschreibung ATC C10AA, C10BA oder C10BX im Beobachtungsjahr (Verordnungsdatum)	KHK Patient:innen
D1-15	V	Rate an KHK Patient:innen, die mindestens einmal Statine, Betablocker und ACE Hemmer/AT1 Blocker im Beobachtungsjahr verordnet bekamen	KHK Patient:innen mit mindestens einer Verordnung Statine (ATC C10AA / C10BA / C10BX), Betablocker (C07A) und ACE-Hemmer/AT1 Blocker (C09A / C09B / C09C / C09D) innerhalb des Beobachtungsjahres	KHK Patient:innen
D1-16	V	Rate der KHK Patient:innen mit Myokardinfarkt im Beobachtungsjahr, die im gleichen und / oder folgenden Quartal Betablocker verschrieben bekamen	KHK Patient:innen mit Myokardinfarkt (ICD I21*, I22*, I23* als Haupt- oder Nebendiagnose stationär), die im gleichen oder folgenden Quartal (Verordnungsdatum) Betablocker (ATC C07*) verschrieben bekamen. Bei Patient:innen mit mehreren Myokardinfarkten wird nur der erste betrachtet. Patient:innen mit Indexevent im 4. Quartal nicht beachtet.	KHK Patient:innen, mit Myokardinfarkt (ICD I21, I22, I23 als Haupt- oder Nebendiagnose stationär) im Beobachtungsjahr
D1-17	V	Rate der KHK Patient:innen mit Myokardinfarkt im Beobachtungsjahr und anschließender Verschreibung von ACE Hemmern oder ARB Therapie	KHK Patient:innen, die einen Myokardinfarkt (stationär als Hauptdiagnose oder Nebendiagnose ICD I21*, I22*, I23*) diagnostiziert bekamen und im gleichen oder Folgequartal ACE Hemmer/ARB	KHK Patient:innen mit Myokardinfarkt (ICD I21*, I22*, I23* als Hauptdiagnose oder Nebendiagnose stationär im Krankenhaus) im Beobachtungsjahr

Nr.	Kat.*	Indikatorbeschreibung	Zähler	Nenner
			Therapie (ATC C09A / C09B / C09C / C09D) verschrieben bekamen. Bei Patient:innen mit mehreren Myokardinfarkten wird nur der erste betrachtet. Patient:innen mit Myokardinfarkt im 4. Quartal nicht beachtet.	
D1-18	V	Anteil der KHK Patient:innen mit Diabetes mellitus Typ-2, die im Beobachtungsjahr Lipidsenker verordnet bekamen	KHK Patient:innen, die auch Diabetes Typ-2 Patient:innen sind und mindestens einmal im Beobachtungsjahr Lipidsenker (ATC C10A / C10B) verordnet bekamen	KHK Patient:innen mit mindestens 2 ambulanten gesicherten oder einer stationären E11.* Diagnose/n
D1-19	V	Rate der KHK Patient:innen mit Hypertonie, die im Beobachtungsjahr mindestens einmal Antihypertensiva verschrieben bekamen	KHK Patient:innen mit Hypertonie mit mindestens einer Verschreibung ATC C02 / C03 / C07 / C08 / C09 im Beobachtungsjahr	Patient:innen mit KHK und Hypertonie
D1-20	O	Anteil der KHK Patient:innen, die im Beobachtungsjahr eine PCI erhielten und den Folgemonat überlebten	KHK Patient:innen, die im Beobachtungsjahr mindestens einmal eine PCI erhielten (OPS 8-837) mindestens 30 Tage überlebten	KHK Patient:innen, die im Beobachtungsjahr mindestens einmal eine PCI erhielten (OPS 8-837). Patient:innen mit PCI im letzten Beobachtungsmonat ausgeschlossen.
D1-21	O	Anteil der KHK Patient:innen, die im Beobachtungsjahr eine PCI erhielten und im selben halben Jahr nicht verstarben	KHK Patient:innen, die im Beobachtungsjahr mindestens einmal eine PCI erhielten (OPS 8-837*, 8-83d*) und im selben halben Jahr nicht verstarben	KHK Patient:innen, die im Beobachtungsjahr mindestens einmal eine PCI erhielten (OPS 8-837*, 8-83d*)
D1-22	/G	Anteil der KHK Patient:innen ohne Krankenhausaufenthalt aufgrund von ischämischen Herzkrankheiten innerhalb des Beobachtungsjahres (risikoadjustiert)	Patient:innen ohne einen stationären Krankenhausfall im Beobachtungsjahr aufgrund von ICDs (Hauptdiagnose) der entsprechenden Krankheitsgruppe	KHK Patient:innen
D1-23	/G	Anteil der Patient:innen mit weniger als zwei Krankenhausaufenthalten aufgrund von ischämischen Herzkrankheiten innerhalb des Beobachtungsjahres (risikoadjustiert)	Patient:innen mit weniger als zwei stationären Krankenhausaufenthalten aufgrund von ICDs (Hauptdiagnose) der Krankheitsgruppe	KHK Patient:innen
**02 Herzinsuffizienz**				
D2-1	V	Patient:innen mit Herzinsuffizienz: Anteil der Patient:innen im Verhältnis zu den Netzwerkpatient:innen	Herzinsuffizienz Patient:innen	Netzwerkpatient:innen
D2-2	V	Herzinsuffizienz: Anzahl der gemeinsam behandelten Patient:innen mit mindestens einer der Diagnosen im Netzwerk	Anzahl der Patient:innen in der Krankheitsgruppe	
D2-3	G	Anteil der multimorbiden Patient:innen: Herzinsuffizienz	Patient:innen der Krankheitsgruppe, die mindestens zwei verschiedene Krankheitsgruppen im Beobachtungsjahr kodiert bekamen	Herzinsuffizienz Patient:innen
D2-4	G	Mortalitätsrate Herzinsuffizienz Patient:innen im Beobachtungsjahr	Patient:innen der Krankheitsgruppe, die im Beobachtungsjahr verstarben	Patient:innen der Krankheitsgruppe
D2-5	V	Rate der Herzinsuffizienz Patient:innen mit mindestens einem Abrechnungskontakt zu einem/einer Fachärzt:in für (1) Allgemeinmedizin / Praktische/r Ärzt:in (Hausärzt:in) / hausärztliche Innere Medizin (2) Internisten / fachärztliche Innere Medizin (3) Kardiologie (4) Nephrologie / (5) Psychosomatische Medizin und Psychotherapie (6) Radiologie (7)	Patient:innen der Krankheitsgruppe, für die mindestens eine Abrechnungsposition von einem / einer Fachärzt:in für (1) – (7) abgerechnet wurde	Herzinsuffizienz Patient:innen

Nr.	Kat.*	Indikatorbeschreibung	Zähler	Nenner
		Physikalische und Rehabilitative Medizin innerhalb des Beobachtungsjahrs		
D2-6	V	Rate der KHK Patient:innen mit mindestens einem Abrechnungskontakt zu zwei verschiedenen Ärzt:innen (1) – (7) innerhalb des Beobachtungsjahrs	Herzinsuffizienz Patient:innen, für die mindestens eine Abrechnungsposition von zwei Fachärzt:innen für (1) – (7) unterschiedlicher BSNR und mit der Art der ärztlichen Inanspruchnahme "O" abgerechnet wurde	Herzinsuffizienz Patient:innen
D2-7	V	Rate der Herzinsuffizienz Patient:innen mit mindestens einem Abrechnungskontakt zu einem/r Ärzt:in (2) – (7) mit Überweisung innerhalb des Beobachtungsjahrs	Herzinsuffizienz Patient:innen, für die mindestens eine Abrechnungsposition von einem/r (2) – (7) mit Überweisung abgerechnet wurde	Herzinsuffizienz Patient:innen
D2-8	V	Anteil der Patient:innen mit Abrechnungskontakt in mindestens einem von vier Quartalen bei oben genannten Arztgruppen	Patient:innen mit Abrechnungskontakt in mindestens einem von vier Quartalen	Patient:innen der Krankheitsgruppe
D2-9	V	Rate der Herzinsuffizienz Patient:innen mit stationärem Krankenhausaufenthalt aufgrund von Herzinsuffizienz und Weiterbehandlung bei einem/r ambulanten Ärzt:in im gleichen oder Folgequartal	Herzinsuffizienz Patient:innen, die einen stationären Krankenhausaufenthalt mit Hauptdiagnose ICD I50* hatten und im gleichen Quartal oder im Folgequartal bei einem/r ambulanten Ärzt:in die Diagnose I50* kodiert bekamen. Patient:innen eingeschränkt auf Indexevent spätestens im vorletzten Beobachtungsquartal.	Herzinsuffizienz Patient:innen, die einen stationären Krankenhausaufenthalt im ersten, zweiten oder dritten Quartal des Beobachtungszeitraums mit Hauptdiagnose I50* hatten
D2-10	V	Rate der Herzinsuffizienz Patient:innen mit mindestens einer Laborleistung (Kalium / Natrium / Kreatinin) im Beobachtungsjahr	Herzinsuffizienz Patient:innen mit mindestens einer abgerechneten Laborleistung (EBM 32066 / 32067 / 32081 oder 32083)	Herzinsuffizienz Patient:innen
D2-11	V	Rate der Herzinsuffizienz Patient:innen, die mindestens eine Echokardiografie im Beobachtungsjahr ambulant bei einem/r Kardiolog:in erhielten	Herzinsuffizienz Patient:innen, die innerhalb des Beobachtungsjahres mindestens EBM Abrechnungsposition EBM 13545 oder 13550 (Echokardiografie) bei einem/r Fachärzt:in der Kardiologie abgerechnet bekamen	Herzinsuffizienz Patient:innen
D2-12	V	Rate der Herzinsuffizienz Patient:innen mit mindestens einem Hausbesuch mit der Diagnose Herzinsuffizienz im Beobachtungsjahr	Anzahl der Hausbesuche (EBM 01410 / 01411 / 01413 / 01415 / 01418 / 38100 / 38105 / 03062 / 03063) mit der Diagnose Herzinsuffizienz bei Herzinsuffizienz Patient:innen	Herzinsuffizienz Patient:innen
D2-13	V	Rate der Herzinsuffizienz Patient:innen, die mindestens einmal im Beobachtungsjahr sowohl Betablocker, als auch ACE Hemmer / AT1-Blocker und Diuretikum verschrieben bekamen	Herzinsuffizienz Patient:innen mit mindestens einer Verschreibung Beta-Blocker (ATC C07) und ACE-Hemmer/AT1 Blocker (C09A / C09B / C09C oder C09D) und Diuretikum, Aldosteronantagonist (C03) im Beobachtungsjahr	Herzinsuffizienz Patient:innen
D2-14	V	Rate der Herzinsuffizienz Patient:innen, die Betablocker im Beobachtungsjahr verordnet bekamen	Herzinsuffizienz Patient:innen mit mindestens einer Verschreibung ATC C07 im Beobachtungsjahr	Herzinsuffizienz Patient:innen
D2-15	V	Rate der Herzinsuffizienz Patient:innen, die ACE Hemmer oder AT1 Blocker im Beobachtungsjahr verordnet bekamen	Herzinsuffizienz Patient:innen mit mindestens einer Verschreibung ATC C09A / C09B / C09C oder C09D im Beobachtungsjahr	Herzinsuffizienz Patient:innen

Nr.	Kat.*	Indikatorbeschreibung	Zähler	Nenner
D2-16	V	Rate der Herzinsuffizienz Patient:innen, die im Beobachtungsjahr NSAID verordnet bekamen	Herzinsuffizienz Patient:innen, die mindestens einmal im Beobachtungsjahr ATC M01A oder M01B verordnet bekamen	Herzinsuffizienz Patient:innen
D2-17	V	Rate der Herzinsuffizienz Patient:innen, die im Beobachtungsjahr Glitazone oder Thiazolidindione verordnet bekamen	Herzinsuffizienz Patient:innen die mindestens einmal im Beobachtungsjahr eine der folgenden ATC verordnet bekamen: A10BG / A10BD03 / A10BD04 / A10BD05 / A10BD06 / A10BD09 oder A10BD12	Herzinsuffizienz Patient:innen
D2-18	V	Rate der Herzinsuffizienz Patient:innen, die im Beobachtungsjahr Diltiazem oder Verapamil verordnet bekamen	Herzinsuffizienz Patient:innen, die mindestens einmal im Beobachtungsjahr ATC C08D verordnet bekamen	Herzinsuffizienz Patient:innen
D2-19	V	Rate der Herzinsuffizienz Patient:innen mit Vorhofflimmern, denen im Beobachtungsjahr orale Antikoagulantien verordnet wurden	Herzinsuffizienz Patient:innen und Vorhofflimmern (Diagnose ambulant gesichert I48*) mit mindestens einer Verschreibung Vitamin K Antagonisten (ATC B01AA), Faktor-Xa-Hemmer (B01AF) oder Thrombinhemmer (B01AE) im Beobachtungsjahr	Patient:innen mit Herzinsuffizienz und Vorhofflimmern (ambulant gesichert I48)
D2-20	O	Anzahl der Patient:innen mit mind. Einem ambulanten Fall in der Notaufnahme von Krankenhäusern im Beobachtungsjahr bei Patient:innen mit Herzinsuffizienz	Anzahl der Herzinsuffizienz Patient:innen, bei denen ambulant von einem Krankenhaus ein Notfall abgerechnet wurde (EBM 01205 / 01207 / 01210 / 01212 / 01214 / 01216 / 01218)	Herzinsuffizienz Patient:innen
D2-21	O	Anteil der Herzinsuffizienz Patient:innen ohne Krankenhausaufenthalt aufgrund von Herzinsuffizienz innerhalb des Beobachtungsjahres (risikoadjustiert)	Patient:innen ohne einen stationären Krankenhausfall im Beobachtungsjahr aufgrund von ICDs (Hauptdiagnose) der entsprechenden Krankheitsgruppe	Herzinsuffizienz Patient:innen
D2-22	O	Anteil der Herzinsuffizienz Patient:innen mit weniger als zwei Krankenhausaufenthalten aufgrund von Herzinsuffizienz innerhalb des Beobachtungsjahres (risikoadjustiert)	Patient:innen mit weniger als zwei stationären Krankenhausaufenthalten aufgrund von ICDs (Hauptdiagnose) der Krankheitsgruppe	Herzinsuffizienz Patient:innen
**03 Sonstige Herz-Kreislauf-Erkrankungen**				
D3-1	V	Patient:innen mit sonstigen Herz-Kreislauf-Erkrankungen: Anteil der Patient:innen im Verhältnis zu den Netzwerkpatient:innen	Anzahl der gemeinsam behandelten Patient:innen mit mindestens einer der Diagnosen im Netzwerk	Anzahl der Patient:innen im Netzwerk
D3-2	V	Sonstige Herz-Kreislauf-Erkrankungen: Anzahl der gemeinsam behandelten Patient:innen mit mindestens einer der Diagnosen im Netzwerk	Anzahl der Patienten in der Krankheitsgruppe im Netzwerk	
D3-3	G	Anteil der multimorbiden Patient:innen: Sonstige Herz-Kreislauf-Erkrankungen	Patient:innen der Krankheitsgruppe, die mindestens zwei verschiedene Krankheitsgruppen im Beobachtungsjahr kodiert bekamen	Patient:innen der Krankheitsgruppe
D3-4	G	Mortalitätsrate Patient:innen mit sonstigen Herz- Kreislauferkrankungen	Patient:innen der Krankheitsgruppe, die im Beobachtungsjahr verstarben	Patient:innen der Krankheitsgruppe
D3-5	V	Rate der Patient:innen der Krankheitsgruppe mit mindestens einem Abrechnungskontakt zu einem/einer Fachärzt:in für (1) Allgemeinmedizin / Praktische/r Ärzt:in (Hausärzt:in) / hausärztliche Innere Medizin (2) Internist / fachärztliche Innere Medizin (3) Kardiologie (4) Psychosomatische	Patient:innen der Krankheitsgruppe, für die mindestens eine Abrechnungsposition von einem / einer Fachärzt:in für (1) – (6) abgerechnet wurde	Patient:innen der Krankheitsgruppe

Nr.	Kat.*	Indikatorbeschreibung	Zähler	Nenner
		Medizin und Psychotherapie (5) Radiologie (6) Physikalische und Rehabilitative Medizin innerhalb des Beobachtungsjahrs		
D3-6	V	Rate der Patient:innen der Krankheitsgruppe mit mindestens einem Abrechnungskontakt zu zwei verschiedenen Fachärzt:innen (1) – (6) innerhalb des Beobachtungsjahrs	Patient:innen der Krankheitsgruppe, für die mindestens eine Abrechnungsposition von zwei verschiedenen Fachärzt:innen für (1) – (6) unterschiedlicher BSNR und mit der Art der ärztlichen Inanspruchnahme "O" abgerechnet wurde	Patient:innen der Krankheitsgruppe
D3-7	V	Rate der Patient:innen der Krankheitsgruppe mit mindestens einem Abrechnungskontakt zu einem/ r Fachärzt:in (2) – (6) mit Überweisung innerhalb des Beobachtungsjahrs	Patient:innen der Krankheitsgruppe, für die mindestens eine Abrechnungsposition von einem/r (2) – (6) mit Überweisung abgerechnet wurde	Patient:innen der Krankheitsgruppe
D3-8	V	Anteil der Patient:innen mit Abrechnungskontakt in mindestens einem von vier Quartalen bei oben genannten Arztgruppen	Patient:innen mit Abrechnungskontakt in mindestens einem von vier Quartalen	Patient:innen der Krankheitsgruppe
D3-9	O	Anteil der Patient:innen mit sonstigen Herz-Kreislauf Erkrankungen ohne Krankenhausaufenthalt aufgrund von sonstigen Herz-Kreislauf Erkrankungen innerhalb des Beobachtungsjahres (risikoadjustiert)	Patient:innen ohne einen stationären Krankenhausfall im Beobachtungsjahr aufgrund von ICDs (Hauptdiagnose) der entsprechenden Krankheitsgruppe	Patient:innen mit sonstigen Herz-Kreislauf Erkrankungen
D3-10	O	Anteil der Patient:innen mit sonstigen Herz-Kreislauf Erkrankungen mit weniger als zwei Krankenhausaufenthalten aufgrund von sonstigen Herz-Kreislauf Erkrankungen innerhalb des Beobachtungsjahres (risikoadjustiert)	Patient:innen mit weniger als zwei stationären Krankenhausaufenthalten aufgrund von ICDs (Hauptdiagnose) der Krankheitsgruppe	Patient:innen mit sonstigen Herz-Kreislauf Erkrankungen
**04 Bronchitis & COPD**				
D4-1	V	Bronchitis & COPD: Anzahl der gemeinsam behandelten Patient:innen mit mindestens einer der Diagnosen im Netzwerk	Anzahl der gemeinsam behandelten Patient:innen mit mindestens einer der Diagnosen im Netzwerk	
D4-2	V	Patient:innen mit Bronchitis & COPD: Anteil der Patient:innen im Verhältnis zu den Netzwerkpatient:innen	Anzahl der gemeinsam behandelten Patient:innen mit mindestens einer der Diagnosen im Netzwerk	Anzahl der Patient:innen im Netzwerk
D4-3	G	Anteil der multimorbiden Patient:innen: Bronchitis & COPD	Patient:innen der Krankheitsgruppe, die mindestens zwei verschiedene Krankheitsgruppen im Beobachtungsjahr kodiert bekamen	Patient:innen der Krankheitsgruppe
D4-4	V	Rate der COPD Patient:innen, die im Beobachtungsjahr im COPD-DMP eingeschrieben waren	COPD Patient:innen, die im Beobachtungszeitraum im COPD-DMP Programm eingeschrieben waren	COPD Patient:innen der Gruppe Bronchitis / COPD und mindestens einer Diagnose (stationär oder ambulant) J43*, J44* oder J47*
D4-5	G	Mortalitätsrate Patient:innen mit Bronchitis / COPD	Patient:innen der Krankheitsgruppe, die im Beobachtungsjahr verstarben	Patient:innen der Krankheitsgruppe
D4-6	V	Rate der Patient:innen der Krankheitsgruppe mit mindestens einem Abrechnungskontakt zu einem/einer Fachärzt:in für (1) Allgemeinmedizin / Praktische/r Ärzt:in (Hausärzt:in) / hausärztliche Innere Medizin (2) Internist / fachärztliche Innere Medizin (3) Kardiologie (4) Pneumologie (5) Radiologie (6) Physikalische und	Patient:innen der Krankheitsgruppe, für die mindestens eine Abrechnungsposition von einem / einer Fachärzt:in für (1) – (6) abgerechnet wurde	Patient:innen der Krankheitsgruppe

Nr.	Kat.*	Indikatorbeschreibung	Zähler	Nenner
		Rehabilitative Medizin innerhalb des Beobachtungsjahrs		
D4-7	V	Rate der Patient:innen der Krankheitsgruppe mit mindestens einem Abrechnungskontakt zu zwei verschiedenen Fachärzt:innen für (1) – (6) innerhalb des Beobachtungsjahrs	Patient:innen der Krankheitsgruppe, für die mindestens eine Abrechnungsposition von zwei verschiedenen Fachärzt:innen für (1) – (6) unterschiedlicher BSNR und mit der Art der ärztlichen Inanspruchnahme "O" abgerechnet wurde	Patient:innen der Krankheitsgruppe
D4-8	V	Rate der Patient:innen der Krankheitsgruppe mit mindestens einem Abrechnungskontakt zu einem/ Fachärzt:in für (2) – (6) mit Überweisung innerhalb des Beobachtungsjahrs	Patient:innen der Krankheitsgruppe, für die mindestens eine Abrechnungsposition von einem/r Fachärzt:in für (2) – (6) mit Überweisung abgerechnet wurde	Patient:innen der Krankheitsgruppe
D4-9	V	Rate der COPD Patient:innen mit mindestens einem Abrechnungskontakt eines/r Pneumolog:in	Anzahl an COPD Patient:innen, für die mindestens eine Abrechnungsposition von einem/r Fachärzt:in der Pneumologie abgerechnet wurde	COPD Patient:innen der Gruppe Bronchitis / COPD und mindestens einer Diagnose (stationär oder ambulant) J43*, J44*, J47*
D4-10	V	Anteil der Patient:innen mit Abrechnungskontakt in mindestens einem von vier Quartalen bei oben genannten Arztgruppen	Patient:innen mit Abrechnungskontakt in mindestens einem von vier Quartalen	Patient:innen der Krankheitsgruppe
D4-11	V	Rate der COPD Patient:innen, die im Beobachtungsjahr an einer DMP Schulung teilnahmen	COPD Patient:innen, für die im Beobachtungszeitraum eine DMP Schulung abgerechnet wurde (EBM 91232 (KVWL) / 91232E (KVWL) / 91232W (KVWL) / 91234E/N/W (KVWL) / 91235E/N/W (KVWL) / 90240 (KV NO) / 90241 (KV NO) / 90242 (KV NO) / 99435 (KV HH) / 99436 (KV HH) / 99798J (KV SH) / oder 99798Q (KV SH) / 99798S (KVSH)/ 99798K (KVSH))	COPD Patient:innen mit DMP Einschreibung im Beobachtungsjahr
D4-12	V	Rate der Bronchitis / COPD Patient:innen, die im Beobachtungsjahr eine Grippeimpfung erhielten	Patient:innen mit Bronchitis/COPD als Hauptdiagnose im Krankenhaus oder gesichert ambulant, die im Beobachtungsjahr mindestens eine Grippeimpfung (EBM 89111 / 89112 oder ATC J07BB) erhielten	Bronchitis / COPD Patient:innen
D4-13	V	Rate der COPD Patient:innen, bei denen im Beobachtungsjahr mindestens eine Lungenfunktionsprüfung (ambulant) durchgeführt wurde	Anzahl an COPD Patient:innen, für die mindestens einmal im Beobachtungsjahr eine Lungenfunktionsprüfung (EBM 13650 / 03330 (KVNO)) abgerechnet wurde	COPD Patient:innen der Gruppe Bronchitis / COPD und der Diagnose (stationär oder ambulant) J43*, J44*, J47*
D4-14	V	Rate der COPD Patient:innen, bei denen im Beobachtungsjahr ambulant ein Thorax Röntgen erstellt wurde	COPD Patient:innen, bei denen ein:e Ärzt:in (ambulant) mindestens einmal im Beobachtungsjahr ein Thorax Röntgen (EBM 34220) abgerechnet hat	COPD Patient:innen definiert über Gruppe der Bronchitis / COPD Patient:innen und der Diagnose (stationär oder ambulant) J43*, J44*, J47*
D4-15	V	Rate der COPD Patient:innen mit mindestens einem Hausbesuch mit der Diagnose COPD im Beobachtungsjahr	Hausbesuche mit der Diagnose ICD J43*, J44*, J47* bei COPD Patient:innen (EBM 01410 / 01411 / 01413 / 01415 / 01418 / 38100 / 38105 / 03062 oder 03063)	COPD Patient:innen der Gruppe Bronchitis / COPD und mindestens einer Diagnose (stationär oder ambulant) J43*, J44*, J47*
D4-16	V	Rate der Patient:innen mit akuter Bronchitis/Bronchiolitis, die im Quartal der Diagnosestellung Antibiotika verordnet bekamen	Patient:innen mit akuter Bronchitis/Bronchiolitis, die im Quartal der Diagnosestellung Antibiotika (ATC J01*) verordnet bekamen	Patient:innen mit akuter Bronchitis/Bronchiolitis Patient:innen der Gruppe Bronchitis / COPD mit mindestens einer der folgenden Diagnosen

Nr.	Kat.*	Indikatorbeschreibung	Zähler	Nenner
				ambulant oder stationär: J20* oder J21*
D4-17	V	Rate der Patient:innen mit akuter Bronchitis/Bronchiolitis, die im Quartal der Diagnosestellung leitliniengerechte Antibiotika verordnet bekamen	Patient:innen mit akuter Bronchitis/Bronchiolitis, die im Quartal der Diagnosestellung leitliniengerechte Antibiotika (ATC J01CA / J01CR / J01FA / J01DB / J01DC / J01DD / J01DE / J01AA oder J01MA) verordnet bekamen	Patient:innen mit akuter Bronchitis/Bronchiolitis Patient:innen, der Gruppe Bronchitis / COPD, die mindestens eine Diagnose J20* oder J21* ambulant oder stationär kodiert bekamen
D4-18	V	Rate der COPD Patient:innen, die im Beobachtungsjahr mindestens einmal Beta2Mimetika oder Anticholinergika verordnet bekamen	COPD Patient:innen, die im Beobachtungsjahr Medikation der ATC R03A oder R03BB verordnet bekamen	COPD Patient:innen der Gruppe Bronchitis / COPD und der Diagnose (stationär oder ambulant) J43*, J44*, J47*
D4-19	V	Rate der COPD Patient:innen, die mindestens einmal pro Quartal im Beobachtungsjahr inhalative Medikation (Beta2Mimetika, Anticholinergika oder Inhalative Glucocorticoide) verordnet bekamen	COPD Patient:innen, die (basierend auf dem Verordnungsdatum) mindestens einmal in jedem Quartal im Beobachtungsjahr inhalative Medikation (ATC R03A, R03B) verordnet bekamen	COPD Patient:innen aus der Gruppe der COPD / Bronchitis mit der ambulanten oder stationären Diagnose J43*, J44*, J47*
D4-20	O	Anzahl der ambulanten Notfälle im Krankenhaus mit einer COPD Diagnose J44* bei COPD Patient:innen	Anzahl der Patient:innen, bei denen akute Exazerbationen (ICD J44*) als gesicherte ambulante Diagnose kodiert wurde und bei gleicher Fallnummer ein Notfall abgerechnet wurde (EBM 01205 / 01207 / 01210 / 01212 / 01214 / 01216 oder 01218)	COPD Patient:innen definiert als Patient:innen mit der Diagnose (stationär oder ambulant) J43*/ J44* / J47*
D4-21	O	Anzahl der ambulanten Notfälle im Krankenhaus bei COPD Patient:innen	Anzahl der COPD Patient:innen, bei denen ambulant im Krankenhaus ein Notfall abgerechnet wurde (EBM 01205 / 01207 / 01210 / 01212 / 01214 / 01216 / 01218)	COPD Patient:innen aus der Gruppe Bronchitis / COPD und der Diagnose (stationär oder ambulant) J43* / J44* / J47*
D4-22	O	Anteil der COPD und Bronchitis Patient:innen ohne Krankenhausaufenthalt aufgrund von Bronchitis / COPD innerhalb des Beobachtungsjahres (risikoadjustiert)	Patient:innen ohne einen stationären Krankenhausfall im Beobachtungsjahr aufgrund von ICDs (Hauptdiagnose) der entsprechenden Krankheitsgruppe	Patient:innen der Gruppe Bronchitis und COPD
D4-23	O	Anteil der COPD und Bronchitis Patient:innen mit weniger als zwei Krankenhausaufenthalten aufgrund von Bronchitis / COPD innerhalb des Beobachtungsjahres (risikoadjustiert)	Patient:innen mit weniger als zwei stationären Krankenhausaufenthalten aufgrund von ICDs (Hauptdiagnose) der Krankheitsgruppe	Patient:innen der Gruppe Bronchitis und COPD
D4-24	O	Anteil der Patient:innen mit weniger als zwei Krankenhausaufenthalten aufgrund von COPD innerhalb des Beobachtungsjahres (risikoadjustiert)	Patient:innen mit mehreren Fällen mit Hauptdiagnose COPD J43* / J44* / J47*	COPD Patient:innen aus der Gruppe Bronchitis / COPD und der Diagnose (stationär oder ambulant) J43* / J44* / J47*
**05 Psychische u. Verhaltensstörungen durch Alkohol u. Opioide**				
D5-1	V	Psychische u. Verhaltensstörungen durch Alkohol o. Opioide: Anzahl der gemeinsam behandelten Patient:innen mit mindestens einer der Diagnosen im Netzwerk	Anzahl der gemeinsam behandelten Patient:innen mit mindestens einer der Diagnosen im Netzwerk	
D5-2	V	Patient:innen mit psychischen u. Verhaltensstörungen durch Alkohol o. Opioide: Anteil der Patient:innen im Verhältnis zu den Netzwerkpatient:innen	Anzahl der gemeinsam behandelten Patient:innen mit mindestens einer der Diagnosen im Netzwerk	Anzahl der Patient:innen im Netzwerk
D5-3	G	Anteil der multimorbiden Patient:innen: Psychische u. Verhaltensstörungen durch Alkohol o. Opioide	Patient:innen der Krankheitsgruppe, die mindestens zwei verschiedene Krankheitsgruppen im Beobachtungsjahr kodiert bekamen	Patient:innen der Krankheitsgruppe

Nr.	Kat.*	Indikatorbeschreibung	Zähler	Nenner
D5-4	G	Mortalitätsrate Patient:innen mit Psychischen u. Verhaltensstörungen durch Alkohol u. Opioide	Patient:innen der Krankheitsgruppe, die im Beobachtungsjahr verstarben	Patient:innen der Krankheitsgruppe
D5-5	V	Rate der Patient:innen der Krankheitsgruppe mit mindestens einem Abrechnungskontakt zu einem/einer Fachärzt:in für (1) Allgemeinmedizin / Praktische/r Ärzt:in (Hausärzt:in) / hausärztliche Innere Medizin (2) Hals-Nasen-Ohren-Heilkunde (3) Neurologie / Neurochirurgie (4) Psychiatrie und Psychotherapie (5) Nervenheilkunde oder Facharzt für Neurologie und Psychiatrie (6) Psychosomatische Medizin und Psychotherapie (7) Psychotherapeutisch tätigen Arzt / Psychologischen Psychotherapeut innerhalb des Beobachtungsjahrs	Patient:innen der Krankheitsgruppe, für die mindestens eine Abrechnungsposition von einem / einer Fachärzt:in für (1) – (7) abgerechnet wurde	Patient:innen der Krankheitsgruppe
D5-6	V	Rate der Patient:innen der Krankheitsgruppe mit mindestens einem Abrechnungskontakt zu zwei verschiedenen (1) – (7) innerhalb des Beobachtungsjahrs	Patient:innen der Krankheitsgruppe, für die mindestens eine Abrechnungsposition von zwei verschiedenen Fachärzt:innen für (1) – (7) unterschiedlicher BSNR und mit der Art der ärztlichen Inanspruchnahme "O" abgerechnet wurde	Patient:innen der Krankheitsgruppe
D5-7	V	Rate der Patient:innen der Krankheitsgruppe mit mindestens einem Abrechnungskontakt zu einem/r Fachärzt:in für (2) – (7) mit Überweisung innerhalb des Beobachtungsjahrs	Patient:innen der Krankheitsgruppe, für die mindestens eine Abrechnungsposition von einem/r Fachärzt:in für (2) – (7) mit Überweisung abgerechnet wurde	Patient:innen der Krankheitsgruppe
D5-8	V	Anteil der Patient:innen mit Abrechnungskontakt in mindestens einem von vier Quartalen bei oben genannten Arztgruppen	Patient:innen mit Abrechnungskontakt in mindestens einem von vier Quartalen	Patient:innen der Krankheitsgruppe
D5-9	V	Rate der Patient:innen mit Psychischen u. Verhaltensstörungen durch Alkohol u. Opioide mit psychosomatischen Gespräch im Beobachtungsjahr	Patient:innen mit Psychischen u. Verhaltensstörungen durch Alkohol u. Opioide, für die ambulant im Beobachtungsjahr mindestens einmal eine der folgenden EBM Abrechnungspositionen abgerechnet wurde: 35100, 35110, 22221, 22222	Patient:innen mit Psychischen u. Verhaltensstörungen durch Alkohol u. Opioide
D5-10	O	Anteil der Patient:innen mit Psychischen und Verhaltensstörungen durch Alkohol und Opioide ohne Krankenhausaufenthalt aufgrund von Psychischen und Verhaltensstörungen durch Alkohol und Opioide innerhalb des Beobachtungsjahres (risikoadjustiert)	Patient:innen ohne einen stationären Krankenhausfall im Beobachtungsjahr aufgrund von ICDs (Hauptdiagnose) der entsprechenden Krankheitsgruppe	Psychische u. Verhaltensstörungen durch Alkohol u. Opioide Patient:innen
D5-11	O	Anteil der Patient:innen mit Psychischen und Verhaltensstörungen durch Alkohol und Opioide mit weniger als zwei Krankenhausaufenthalten aufgrund von Psychischen und Verhaltensstörungen durch Alkohol und Opioide innerhalb des Beobachtungsjahres (risikoadjustiert)	Patient:innen mit weniger als zwei stationären Krankenhausaufenthalten aufgrund von ICDs (Hauptdiagnose) der Krankheitsgruppe	Psychische u. Verhaltensstörungen durch Alkohol u. Opioide Patient:innen
**06 Rückenbeschwerden**				
D6-1	V	Rückenbeschwerden: Anzahl der gemeinsam behandelten Patient:innen mit mindestens einer der Diagnosen im Netzwerk	Anzahl der gemeinsam behandelten Patient:innen mit mindestens einer der Diagnosen im Netzwerk	

Nr.	Kat.*	Indikatorbeschreibung	Zähler	Nenner
D6-2	V	Patient:innen mit Rückenbeschwerden: Anteil der Patient:innen im Verhältnis zu den Netzwerkpatient:innen	Anzahl der gemeinsam behandelten Patient:innen mit mindestens einer der Diagnosen im Netzwerk	Anzahl der Patient:innen im Netzwerk
D6-3	G	Anteil der multimorbiden Patient:innen: Rückenbeschwerden	Patient:innen der Krankheitsgruppe, die mindestens zwei verschiedene Krankheitsgruppen im Beobachtungsjahr kodiert bekamen	Patient:innen der Krankheitsgruppe
D6-4	G	Mortalitätsrate Rückenbeschwerden Patient:innen	Patient:innen der Krankheitsgruppe, die im Beobachtungsjahr verstarben	Patient:innen der Krankheitsgruppe
D6-5	V	Rate der Patient:innen der Krankheitsgruppe mit mindestens einem Abrechnungskontakt zu einem/einer Fachärzt:in für (1) Allgemeinmedizin / Praktische/r Ärzt:in (Hausärzt:in) / hausärztliche Innere Medizin (2) Anästhesiologie (3) Chirurgie (4) Rheumatologie (der Inneren Medizin) (5) Neurologie / Neurochirurgie (6) Orthopädie oder Unfallchirurgie (7) Psychiatrie und Psychotherapie (8) Nervenheilkunde oder Facharzt für Neurologie und Psychiatrie (9) Psychosomatische Medizin und Psychotherapie (10) Psychotherapeutisch tätigen Arzt / Psychologischen Psychotherapeut (11) Radiologie (12) Physikalische und Rehabilitative Medizin innerhalb des Beobachtungsjahrs	Patient:innen der Krankheitsgruppe, für die mindestens eine Abrechnungsposition von einem / einer Fachärzt:in für (1) – (12) abgerechnet wurde	Patient:innen der Krankheitsgruppe
D6-6	V	Rate der Patient:innen der Krankheitsgruppe mit mindestens einem Abrechnungskontakt zu zwei verschiedenen (1) – (12) innerhalb des Beobachtungsjahrs	Patient:innen der Krankheitsgruppe, für die mindestens eine Abrechnungsposition von zwei verschiedenen Fachärzt:innen für (1) – (12) unterschiedlicher BSNR und mit der Art der ärztlichen Inanspruchnahme "O" abgerechnet wurde	Patient:innen der Krankheitsgruppe
D6-7	V	Rate der Patient:innen der Krankheitsgruppe mit mindestens einem Abrechnungskontakt zu einem/r Fachärzt:in (2) – (12) mit Überweisung innerhalb des Beobachtungsjahrs	Patient:innen der Krankheitsgruppe, für die mindestens eine Abrechnungsposition von einem/r Fachärzt:in für (2) – (12) mit Überweisung abgerechnet wurde	Patient:innen der Krankheitsgruppe
D6-8	V	Anteil der Patient:innen mit Abrechnungskontakt in mindestens einem von vier Quartalen bei oben genannten Arztgruppen	Patient:innen mit Abrechnungskontakt in mindestens einem von vier Quartalen	Patient:innen der Krankheitsgruppe
D6-9	V	Rate der Rückenbeschwerden Patient:innen, bei denen im Beobachtungsjahr mindestens einmal eine Röntgenaufnahme der Wirbelsäule ambulant erstellt wurde	Rückenbeschwerden Patient:innen, für die im Beobachtungsjahr mindestens einmal ambulant EBM 34221 oder 34222 abgerechnet wurde	Rückenbeschwerden Patient:innen
D6-10	V	Rate der Rückenbeschwerden Patient:innen, bei denen im Beobachtungsjahr mindestens einmal ambulant ein MRT der Wirbelsäule erstellt wurde	Rückenbeschwerden Patient:innen, für die im Beobachtungsjahr mindestens einmal ambulant EBM 34411 abgerechnet wurde	Rückenbeschwerden Patient:innen
D6-11	V	Rate der Rückenbeschwerden Patient:innen, die im Beobachtungsjahr keine Opioide verordnet bekamen	Rückenbeschwerden Patient:innen, die nicht mindestens einmal im Beobachtungsjahr ATC N02A verordnet bekamen	Rückenbeschwerden Patient:innen

Nr.	Kat.*	Indikatorbeschreibung	Zähler	Nenner
D6-12	O	Anteil der Rückenbeschwerden Patient:innen ohne Krankenhausaufenthalt aufgrund von Rückenbeschwerden innerhalb des Beobachtungsjahres (risikoadjustiert)	Patient:innen ohne einen stationären Krankenhausfall im Beobachtungsjahr aufgrund von ICDs (Hauptdiagnose) der entsprechenden Krankheitsgruppe	Rückenbeschwerden Patient:innen
D6-13	O	Anteil der Rückenbeschwerden Patient:innen mit weniger als zwei Krankenhausaufenthalten aufgrund von Rückenbeschwerden innerhalb des Beobachtungsjahres (risikoadjustiert)	Patient:innen mit weniger als zwei stationären Krankenhausaufenthalten aufgrund von ICDs (Hauptdiagnose) der Krankheitsgruppe	Rückenbeschwerden Patient:innen
**07 Hypertonie (Hochdruckkrankheit)**				
D7-1	V	Hypertonie [Hochdruckkrankheit]: Anzahl der gemeinsam behandelten Patient:innen mit mindestens einer der Diagnosen im Netzwerk	Anzahl der gemeinsam behandelten Patient:innen mit mindestens einer der Diagnosen im Netzwerk	
D7-2	V	Patient:innen mit Hypertonie [Hochdruckkrankheit]: Anteil der Patient:innen im Verhältnis zu den Netzwerkpatient:innen	Anzahl der gemeinsam behandelten Patient:innen mit mindestens einer der Diagnosen im Netzwerk	Anzahl der Patient:innen im Netzwerk
D7-3	G	Multimorbiditätsrate Hypertonie Patient:innen	Patient:innen der Krankheitsgruppe, die mindestens zwei verschiedene Krankheitsgruppen im Beobachtungsjahr kodiert bekamen	Patient:innen der Krankheitsgruppe
D7-4	G	Mortalitätsrate Hypertonie Patient:innen	Patient:innen der Krankheitsgruppe, die im Beobachtungsjahr verstarben	Patient:innen der Krankheitsgruppe
D7-5	V	Rate der Patient:innen der Krankheitsgruppe mit mindestens einem Abrechnungskontakt zu einem/einer (1) Fachärzt:in für Allgemeinmedizin / Praktische/r Ärzt:in (Hausärzt:in) / hausärztliche Innere Medizin (2) Anästhesiologie (3) Augenheilkunde (4) Internisten / fachärztliche Innere Medizin (5) Kardiologie (6) Nephrologie innerhalb des Beobachtungsjahrs	Patient:innen der Krankheitsgruppe, für die mindestens eine Abrechnungsposition von einem / einer Fachärzt:in (1) – (6) abgerechnet wurde	Patient:innen der Krankheitsgruppe
D7-6	V	Rate der Patient:innen der Krankheitsgruppe mit mindestens einem Abrechnungskontakt zu zwei verschiedenen Fachärzt:innen für (1) – (6) innerhalb des Beobachtungsjahrs	Patient:innen der Krankheitsgruppe, für die mindestens eine Abrechnungsposition von zwei verschiedenen Fachärzt:innen für (1) – (6) und mit der Art der ärztlichen Inanspruchnahme "O" abgerechnet wurde	Patient:innen der Krankheitsgruppe
D7-7	V	Rate der Patient:innen der Krankheitsgruppe mit mindestens einem Abrechnungskontakt einem Fachärztin für (2) – (6) mit Überweisung innerhalb des Beobachtungsjahrs	Rate der Patient:innen der Krankheitsgruppe mit mindestens einem Abrechnungskontakt einem Fachärztin für (2) – (6) mit Überweisung innerhalb des Beobachtungsjahrs	Patient:innen der Krankheitsgruppe
D7-8	V	Anteil der Patient:innen mit Abrechnungskontakt in mindestens einem von vier Quartalen bei oben genannten Arztgruppen	Patient:innen mit Abrechnungskontakt in mindestens einem von vier Quartalen	Patient:innen der Krankheitsgruppe
D7-9	V	Rate der Hypertonie Patient:innen, die mindestens einmal pro Quartal im Beobachtungsjahr ein Antihypertensivum verordnet bekamen	Patient:innen mit Hypertonie, die mindestens einmal in jedem Quartal ein Antihypertensivum (ATC C02 / C03 / C07 / C08 / C09) verordnet bekamen	Hypertonie Patient:innen
D7-10	V	Rate der Hypertonie Patient:innen mit Nierenerkrankung, die im Beobachtungsjahr ACE-Hemmer oder ARB-Therapie verordnet bekamen	Hypertonie Patient:innen mit Nierenerkrankung (ICD N18*) stationär (Haupt- oder Nebendiagnose) oder ambulant	Hypertonie Patient:innen mit Nierenerkrankung (ICD Code N18*)

Nr.	Kat.*	Indikatorbeschreibung	Zähler	Nenner
			gesichert), die C09A, C09B, C09C oder C09D verordnet bekamen	
D7-11	V	Subgruppe: Rate der Hypertonie Patient:innen mit fortgeschrittener Nierenerkrankung, die im Beobachtungsjahr ACE-Hemmer oder ARB-Therapie erhielten	Hypertonie Patient:innen mit fortgeschrittener Nierenerkrankung (ICD N18.4 oder N18.5) stationär (Hauptdiagnose oder Nebendiagnose) oder ambulant gesichert), die ATC C09A / C09B / C09C / C09D verordnet bekamen	Hypertonie Patient:innen mit fortgeschrittener Nierenerkrankung (ICD Codes N18.4, N18.5)
D7-12	V	Rate der KHK Patient:innen mit Hypertonie, die im Beobachtungsjahr mindestens einmal Antihypertensiva verschrieben bekamen	KHK Patient:innen mit Hypertonie mit mindestens einer Verschreibung ATC C02 / C03 / C07 / C08 / C09 im Beobachtungsjahr	Patient:innen mit KHK und Hypertonie
D7-13	O	Anteil der Hypertonie Patient:innen ohne Krankenhausaufenthalt aufgrund von Hypertonie innerhalb des Beobachtungsjahres (risikoadjustiert)	Patient:innen ohne einen stationären Krankenhausfall im Beobachtungsjahr aufgrund von ICDs (Hauptdiagnose) der entsprechenden Krankheitsgruppe	Hypertonie Patient:innen
D7-14	O	Anteil der Hypertonie Patient:innen mit weniger als zwei Krankenhausaufenthalten aufgrund von Hypertonie innerhalb des Beobachtungsjahres (risikoadjustiert)	Patient:innen mit weniger als zwei stationären Krankenhausaufenthalten aufgrund von ICDs (Hauptdiagnose) der Krankheitsgruppe	Hypertonie Patient:innen
**08 Gastroenteritis und bestimmte Krankheiten des Darms**				
D8-1	V	Gastroenteritis und bestimmte Krankheiten des Darmes: Anzahl der gemeinsam behandelten Patient:innen mit mindestens einer der Diagnosen im Netzwerk	Anzahl der gemeinsam behandelten Patient:innen mit mindestens einer der Diagnosen im Netzwerk	
D8-2	V	Patient:innen mit Gastroenteritis und bestimmte Krankheiten des Darmes: Anteil der Patient:innen im Verhältnis zu den Netzwerkpatient:innen	Anzahl der gemeinsam behandelten Patient:innen mit mindestens einer der Diagnosen im Netzwerk	Anzahl der Patient:innen im Netzwerk
D8-3	G	Multimorbiditätsrate der Patient:innen mit Gastroenteritis und bestimmte Krankheiten des Darms	Patient:innen der Krankheitsgruppe, die mindestens zwei verschiedene Krankheitsgruppen im Beobachtungsjahr kodiert bekamen	Patient:innen der Krankheitsgruppe
D8-4	G	Mortalitätsrate Patient:innen mit Gastroenteritis und bestimmte Krankheiten des Darms	Patient:innen der Krankheitsgruppe, die im Beobachtungsjahr verstarben	Patient:innen der Krankheitsgruppe
D8-5	V	Rate der Patient:innen der Krankheitsgruppe mit mindestens einem Abrechnungskontakt zu einem/einer Fachärzt:in für (1) Allgemeinmedizin / Praktische/r Ärzt:in (Hausärzt:in) / hausärztliche Innere Medizin (2) Internist / fachärztliche Innere Medizin (3) Gastroenterologie innerhalb des Beobachtungsjahrs	Patient:innen der Krankheitsgruppe, für die mindestens eine Abrechnungsposition von einem / einer Fachärzt:in für (1) – (3) abgerechnet wurde	Patient:innen der Krankheitsgruppe
D8-6	V	Rate der Patient:innen der Krankheitsgruppe mit mindestens einem Abrechnungskontakt zu zwei verschiedenen Fachärzt:innen für (1) – (3) innerhalb des Beobachtungsjahrs	Patient:innen der Krankheitsgruppe, für die mindestens eine Abrechnungsposition von zwei verschiedenen Fachärzt:innen für (1) – (3) unterschiedlicher BSNR und mit der Art der ärztlichen Inanspruchnahme "O" abgerechnet wurde	Patient:innen der Krankheitsgruppe
D8-7	V	Rate der Patient:innen der Krankheitsgruppe mit mindestens einem Abrechnungskontakt zu einem/r Fachärzt:in für (2) – (3) mit Überweisung innerhalb des Beobachtungsjahrs	Patient:innen der Krankheitsgruppe, für die mindestens eine Abrechnungsposition von einem/r Fachärzt:in für (2) – (3) mit Überweisung abgerechnet wurde	Patient:innen der Krankheitsgruppe

Nr.	Kat.*	Indikatorbeschreibung	Zähler	Nenner
D8-8	V	Anteil der Patient:innen mit Abrechnungskontakt in mindestens einem von vier Quartalen bei oben genannten Arztgruppen	Patient:innen mit Abrechnungskontakt in mindestens einem von vier Quartalen	Patient:innen der Krankheitsgruppe
D8-9	V	Rate der Patient:innen mit Gastroenteritis und bestimmten Krankheiten des Darms, die Opioide und gleichzeitig Mittel zur Behandlung der dadurch möglicherweise verursachten Obstipation verordnet bekamen	Gastroenteritis und bestimmte Krankheiten des Darms Patient:innen, die im Beobachtungsjahr ATC N02A und A06A verordnet bekamen	Patient:innen mit Gastroenteritis und bestimmten Krankheiten des Darms, die N02A verordnet bekamen
D8-10	V	Rate der Patient:innen mit Divertikulose / Divertikulitis, die ein Antibiotikum verordnet bekamen	Rate der Patient:innen mit Divertikulose / Divertikulitis, die ein Antibiotikum (ATC J01*) verordnet bekamen	Patient:innen der Krankheitsgruppe mit der Diagnose Divertikulose / Divertikulitis
D8-11	O	Anteil der Patient:innen der Krankheitsgruppe mit stationären Krankenhausaufenthalt mit der Diagnose Divertikulose / Divertikulitits im Beobachtungsjahr	Stationäre Krankenhausfälle im Beobachtungsjahr mit der Haupt- oder Nebendiagnose ICD K57*	Gastroenteritis und bestimmte Krankheiten des Darms Patient:innen
D8-12	O	Anteil der Gastroenteritis und bestimmte Krankheiten des Darms Patient:innen ohne Krankenhausaufenthalt aufgrund von Gastroenteritis und bestimmten Krankheiten des Darms innerhalb des Beobachtungsjahres (risikoadjustiert)	Patient:innen ohne einen stationären Krankenhausfall im Beobachtungsjahr aufgrund von ICDs (Hauptdiagnose) der entsprechenden Krankheitsgruppe	Gastroenteritis und bestimmte Krankheiten des Darms Patient:innen
D8-13	O	Anteil der Gastroenteritis und bestimmte Krankheiten des Darms Patient:innen mit weniger als zwei Krankenhausaufenthalten aufgrund von Gastroenteritis und bestimmten Krankheiten des Darms innerhalb des Beobachtungsjahres (risikoadjustiert)	Patient:innen mit weniger als zwei stationären Krankenhausaufenthalten aufgrund von ICDs (Hauptdiagnose) der Krankheitsgruppe	Gastroenteritis und bestimmte Krankheiten des Darms Patient:innen
**09 Infektiöse Darmkrankheiten**				
D9-1	V	Infektiöse Darmkrankheiten: Anzahl der gemeinsam behandelten Patient:innen mit mindestens einer der Diagnosen im Netzwerk	Anzahl der gemeinsam behandelten Patient:innen mit mindestens einer der Diagnosen im Netzwerk	
D9-2	V	Patient:innen mit infektiösen Darmkrankheiten: Anteil der Patient:innen im Verhältnis zu den Netzwerkpatient:innen	Anzahl der gemeinsam behandelten Patient:innen mit mindestens einer der Diagnosen im Netzwerk	Anzahl der Patient:innen im Netzwerk
D9-3	G	Multimorbiditätsrate Patient:innen mit Infektiösen Darmkrankheiten	Patient:innen der Krankheitsgruppe, die mindestens zwei verschiedene Krankheitsgruppen im Beobachtungsjahr kodiert bekamen	Patient:innen der Krankheitsgruppe
D9-4	G	Mortalitätsrate Patient:innen Infektiöse Darmkrankheiten	Patient:innen der Krankheitsgruppe, die im Beobachtungsjahr verstarben	Patient:innen der Krankheitsgruppe
D9-5	V	Rate der Patient:innen der Krankheitsgruppe mit mindestens einem Abrechnungskontakt zu einem/einer Fachärzt:in für (1) Allgemeinmedizin / Praktische/r Ärzt:in (Hausärzt:in) / hausärztliche Innere Medizin (2) Laboratoriumsmedizin (3) Internist / fachärztliche Innere Medizin (4) Gastroenterologie innerhalb des Beobachtungsjahrs	Patient:innen der Krankheitsgruppe, für die mindestens eine Abrechnungsposition von einem / einer Fachärzt:in für (1) - (4) abgerechnet wurde	Patient:innen der Krankheitsgruppe
D9-6	V	Rate der Patient:innen der Krankheitsgruppe mit mindestens einem Abrechnungskontakt zu zwei verschiedenen Fachärzt:innen für (1) – (4) innerhalb des Beobachtungsjahrs	Patient:innen der Krankheitsgruppe, für die mindestens eine Abrechnungsposition von zwei verschiedenen Fachärzt:innen für (1) - (4) unterschiedlicher BSNR und mit der Art der ärztlichen	Patient:innen der Krankheitsgruppe

Nr.	Kat.*	Indikatorbeschreibung	Zähler	Nenner
			Inanspruchnahme "O" abgerechnet wurde	
D9-7	V	Rate der Patient:innen der Krankheitsgruppe mit mindestens einem Abrechnungskontakt zu einem/r Fachärzt:in für (2) - (4) mit Überweisung innerhalb des Beobachtungsjahrs	Patient:innen der Krankheitsgruppe, für die mindestens eine Abrechnungsposition von einem/r Fachärzt:in für (2) – (4) mit Überweisung abgerechnet wurde	Patient:innen der Krankheitsgruppe
D9-8	V	Anteil der Patient:innen mit Abrechnungskontakt in mindestens einem von vier Quartalen bei oben genannten Arztgruppen	Patient:innen mit Abrechnungskontakt in mindestens einem von vier Quartalen	Patient:innen der Krankheitsgruppe
D9-9	O	Anteil der Patient:innen mit infektiösen Darmkrankheiten ohne Krankenhausaufenthalt aufgrund von infektiösen Darmkrankheiten innerhalb des Beobachtungsjahres (risikoadjustiert)	Patient:innen ohne einen stationären Krankenhausfall im Beobachtungsjahr aufgrund von ICDs (Hauptdiagnose) der entsprechenden Krankheitsgruppe	Patient:innen mit Infektiösen Darmkrankheiten
D9-10	O	Anteil der Patient:innen mit infektiösen Darmkrankheiten mit weniger als zwei Krankenhausaufenthalten aufgrund von infektiösen Darmkrankheiten innerhalb des Beobachtungsjahres (risikoadjustiert)	Patient:innen mit weniger als zwei stationären Krankenhausaufenthalten aufgrund von ICDs (Hauptdiagnose) der Krankheitsgruppe	Patient:innen mit Infektiösen Darmkrankheiten
**10 Grippe und Pneumonie**				
D10-1	V	Grippe und Pneumonie: Anzahl der gemeinsam behandelten Patient:innen mit mindestens einer der Diagnosen im Netzwerk	Anzahl der gemeinsam behandelten Patient:innen mit mindestens einer der Diagnosen im Netzwerk	
D10-2	V	Patient:innen mit Grippe und Pneumonie: Anteil der Patient:innen im Verhältnis zu den Netzwerkpatient:innen	Anzahl der gemeinsam behandelten Patient:innen mit mindestens einer der Diagnosen im Netzwerk	Anzahl der Patient:innen im Netzwerk
D10-3	G	Multimorbiditätsrate Patient:innen mit Grippe und Pneumonie	Patient:innen der Krankheitsgruppe, die mindestens zwei verschiedene Krankheitsgruppen im Beobachtungsjahr kodiert bekamen	Patient:innen der Krankheitsgruppe
D10-4	G	Mortalitätsrate Grippe und Pneumonie Patient:innen	Patient:innen der Krankheitsgruppe, die im Beobachtungsjahr verstarben	Patient:innen der Krankheitsgruppe
D10-5	V	Rate der Patient:innen der Krankheitsgruppe mit mindestens einem Abrechnungskontakt zu einem/einer Fachärzt:in für (1) Allgemeinmedizin / Praktische/r Ärzt:in (Hausärzt:in) / hausärztliche Innere Medizin (2) Hals-Nasen-Ohren-Heilkunde (3) Internist / fachärztliche Innere Medizin (4) Pneumologie (5) Radiologie innerhalb des Beobachtungsjahrs	Patient:innen der Krankheitsgruppe, für die mindestens eine Abrechnungsposition von einem / einer Fachärzt:in für (1) – (5) abgerechnet wurde	Patient:innen der Krankheitsgruppe
D10-6	V	Rate der Patient:innen der Krankheitsgruppe mit mindestens einem Abrechnungskontakt zu zwei verschiedenen Fachärzt:innen für (1) – (5) innerhalb des Beobachtungsjahrs	Patient:innen der Krankheitsgruppe, für die mindestens eine Abrechnungsposition von zwei verschiedenen Fachärzt:innen für (1) – (5) unterschiedlicher BSNR und mit der Art der ärztlichen Inanspruchnahme "O" abgerechnet wurde	Patient:innen der Krankheitsgruppe
D10-7	V	Rate der Patient:innen der Krankheitsgruppe mit mindestens einem Abrechnungskontakt zu einem/r Fachärzt:in für (2) – (5) mit Überweisung innerhalb des Beobachtungsjahrs	Patient:innen der Krankheitsgruppe, für die mindestens eine Abrechnungsposition von einem/r Fachärzt:in für (2) – (5) mit Überweisung abgerechnet wurde	Patient:innen der Krankheitsgruppe
D10-8	V	Anteil der Patient:innen mit Abrechnungskontakt in mindestens	Patient:innen mit Abrechnungskontakt in mindestens einem von vier Quartalen	Patient:innen der Krankheitsgruppe

Nr.	Kat.*	Indikatorbeschreibung	Zähler	Nenner
		einem von vier Quartalen bei oben genannten Arztgruppen		
D10-9	V	Rate der Grippe und Pneumonie Patient:innen, die nach einem stationären Krankenhausaufenthalt aufgrund von Pneumonie im selben oder folgenden Quartal eine:n Hausärzt:in konsultierten	Patient:innen, die als Hauptdiagnose stationär ICD J12* / J13* / J14* / J15* kodiert bekamen und für die im selben oder im folgenden Quartal eine beliebige EBM Abrechnungsposition von einem/ Hausärzt:in (Allgemeinmediziner (Hausärzt:in), Ärzt:in /Praktische:r Ärzt:in (Hausärzt:in), Internist:in (Hausärzt:in)) abgerechnet wurde	Grippe und Pneumonie Patient:innen, die als Hauptdiagnose stationär ICD J12* / J13* / J14* / J15* kodiert bekamen. Nur Patient:innen mit Indexevent im 1. bis 3. Quartal berücksichtigt.
D10-10	V	Rate der Grippe / Pneumonie Patient:innen mit mindestens einem Hausbesuch mit der Diagnose Grippe / Pneumonie im Beobachtungsjahr	Anzahl der Grippe / Pneumonie Patient:innen mit mindestens einem Hausbesuch mit der Diagnose Grippe und Pneumonie Patient:innen (EBM 01410 / 01411 / 01413 / 01415 / 01418 / 38100 / 38105 / 03062 / 03063)	Grippe und Pneumonie Patient:innen
D10-11	V	Rate der Patient:innen mit (viraler oder bakterieller) Pneumonie, die im Beobachtungsjahr ein Antibiotikum verordnet bekamen	Pneumonie Patient:innen, die im Beobachtungsjahr mindestens einmal ein Antibiotikum (ATC J01) verordnet bekamen	Patient:innen mit viraler oder bakterieller Pneumonie definiert als Patient:innen der Gruppe Grippe und Pneumonie und mindestens eine der folgenden Diagnosen ambulant (gesichert) oder stationär (Hauptdiagnose): J12*, J13*, J14*, J15*
D10-12	V	Rate der Patient:innen mit (viraler oder bakterieller) Pneumonie, die im Beobachtungsjahr eines der zur Anwendung bei Pneumonie empfohlenen Antibiotika verordnet bekamen	Pneumonie Patient:innen, die im Beobachtungsjahr mindestens einmal eines der zur Anwendung bei Pneumonie empfohlenen Antibiotika (ATC J01CR / J01FA / J01DB / J01DC / J01DD / J01DE / J01DH / J01AA / J01MA oder J01G) verordnet bekamen	Patient:innen mit viraler oder bakterieller Pneumonie. Patient ist Teil der Gruppe Grippe und Pneumonie Patient:innen und hat mindestens eine der folgenden Diagnosen ambulant (gesichert) oder stationär (Hauptdiagnose) erhalten: J12*, J13*, J14*, J15*
D10-13	O	Anteil der Grippe und Pneunomie Patient:innen ohne Krankenhausaufenthalt aufgrund von Grippe oder Pneumonie innerhalb des Beobachtungsjahres (risikoadjustiert)	Patient:innen ohne einen stationären Krankenhausfall im Beobachtungsjahr aufgrund von ICDs (Hauptdiagnose) der entsprechenden Krankheitsgruppe	Grippe und Pneumonie Patient:innen
D10-14	O	Anteil der Grippe und Pneunomie Patient:innen mit weniger als zwei Krankenhausaufenthalten aufgrund von Grippe oder Pneumonie innerhalb des Beobachtungsjahres (risikoadjustiert)	Patient:innen mit weniger als zwei stationären Krankenhausaufenthalten aufgrund von ICDs (Hauptdiagnose) der Krankheitsgruppe	Grippe und Pneumonie Patient:innen
**11 HNO-Infektionen**				
D11-1	V	HNO-Infektionen: Anzahl der gemeinsam behandelten Patient:innen mit mindestens einer der Diagnosen im Netzwerk	Anzahl der gemeinsam behandelten Patient:innen mit mindestens einer der Diagnosen im Netzwerk	
D11-2	V	Patient:innen mit HNO-Infektionen: Anteil der Patient:innen im Verhältnis zu den Netzwerkpatient:innen	Anzahl der gemeinsam behandelten Patient:innen mit mindestens einer der Diagnosen im Netzwerk	Anzahl der Patient:innen im Netzwerk
D11-3	G	Multimorbiditätsrate Patient:innen mit HNO-Infektionen	Patient:innen der Krankheitsgruppe, die mindestens zwei verschiedene Krankheitsgruppen im Beobachtungsjahr kodiert bekamen	Patient:innen der Krankheitsgruppe

Nr.	Kat.*	Indikatorbeschreibung	Zähler	Nenner
D11-4	G	Mortalitätsrate Patient:innen mit HNO Infektionen	Patient:innen der Krankheitsgruppe, die im Beobachtungsjahr verstarben	Patient:innen der Krankheitsgruppe
D11-5	V	Rate der Patient:innen der Krankheitsgruppe mit mindestens einem Abrechnungskontakt zu einem/einer Fachärzt:in für (1) Allgemeinmedizin / Praktische/r Ärzt:in (Hausärzt:in) / hausärztliche Innere Medizin (2) Hals-Nasen-Ohren-Heilkunde (3) Pneumologie (4) Radiologie innerhalb des Beobachtungsjahrs	Patient:innen der Krankheitsgruppe, für die mindestens eine Abrechnungsposition von einem / einer Fachärzt:in für (1) – (4) abgerechnet wurde	Patient:innen der Krankheitsgruppe
D11-6	V	Rate der Patient:innen der Krankheitsgruppe mit mindestens einem Abrechnungskontakt zu zwei verschiedenen Fachärzt:innen für (1) – (4) innerhalb des Beobachtungsjahrs	Patient:innen der Krankheitsgruppe, für die mindestens eine Abrechnungsposition von zwei verschiedenen Fachärzt:innen für (1) – (4) unterschiedlicher BSNR und mit der Art der ärztlichen Inanspruchnahme "O" abgerechnet wurde	Patient:innen der Krankheitsgruppe
D11-7	V	Rate der Patient:innen der Krankheitsgruppe mit mindestens einem Abrechnungskontakt zu einem/ Fachärzt:in für (2) – (4) mit Überweisung innerhalb des Beobachtungsjahrs	Patient:innen der Krankheitsgruppe, für die mindestens eine Abrechnungsposition von einem/ Fachärzt:in für (2) – (4) mit Überweisung abgerechnet wurde	Patient:innen der Krankheitsgruppe
D11-8	V	Anteil der Patient:innen mit Abrechnungskontakt in mindestens einem von vier Quartalen bei oben genannten Arztgruppen	Patient:innen mit Abrechnungskontakt in mindestens einem von vier Quartalen	Patient:innen der Krankheitsgruppe
D11-9	V	Rate der Sinusitispatient:innen, die im Beobachtungsjahr ein Antibiotikum verschrieben bekamen	Sinusitispatient:innen, die im Beobachtungsjahr ein Antibiotikum (ATC J01) verschrieben bekamen	Sinusitispatient:innen definiert als Patient der Gruppe mit HNO Infektionen und mindestens eine der folgenden Diagnosen ambulant (gesichert) oder stationär (Hauptdiagnose) J32 oder J01
D11-10	V	Rate der HNO-Infektionen Patient:innen mit Otitis media oder Myringitis, die im Beobachtungsjahr ein Antibiotikum verordnet bekamen	Rate der Grippe und Pneumonie Patient:innen mit Otitis media oder Myringitis, die im Beobachtungsjahr ein Antibiotikum (ATC J01) verordnet bekamen	Patient:innen der Gruppe Grippe und Pneumonie mit Otitis media oder Myringits mit mindestens einer Diagnose stationär (Hauptdiagnose) oder ambulant (gesichert) H65*/ H66*/ H73*/ H73*/ H67* / H68* / H69* / H70*
D11-11	V	Rate der HNO-Infektionen Patient:innen mit Otitis media oder Myringitis, die im Beobachtungsjahr empfohlene Antibiotika (Amoxicillin (Penicilline), Sulfonamide/Trimethoprim oder Phenoxymethyl-Penicilline) verordnet bekamen	Rate der Grippe und Pneumonie Patient:innen mit Otitis media oder Myringitis, die im Beobachtungsjahr Amoxicillin (Penicilline) (ATC J01CA / J01E / J01CE) verordnet bekamen. Hierzu zählen Chinolone (ATC J01M) nicht.	Grippe und Pneumonie Patient:innen mit Otitis media oder Myringits mit mindestens einer Diagnose stationär (Hauptdiagnose) oder ambulant (gesichert) H65*/ H66*/ H73*/ H73*/ H67* / H68* / H69* / H70*
D11-12	V	Rate der Pharyngitispatient:innen, die empfohlene Antibiotika (Phenoxymethylpenicillin oder Erythromycin) verordnet bekamen	Phrayngitispatient:innen, die Phenoxymethylpenicillin oder Erythromycin (ATC J01CE02 oder J01FA01) verschrieben bekamen	Pharyngitispatient:innen, definiert als Patient:innen der Gruppe mit HNO Infektionen und mindestens einer der folgenden Diagnosen ambulant (gesichert) oder stationär (Hauptdiagnose) R07.0, J03, J02, J06.0, J35

Nr.	Kat.*	Indikatorbeschreibung	Zähler	Nenner
D11-13	V	Rate der Pharyngitispatient:innen, die Antibiotika verordnet bekamen, welche nicht den empfohlenen Phenoxymethylpenicillin oder Erythromycin entsprechen	Pharyngitis Patient:innen, die ATC J01A / J01D / J01E / J01F / J01M / J01X, aber nicht J01CE02 / J01FA01 verordnet bekamen	Pharyngitispatient:innen definiert als Patient mit mindestens eine der folgenden Diagnosen ambulant (gesichert) oder stationär (Hauptdiagnose) R07.0, J03, J02, J06.0, J35
D11-13	O	Anteil der Patient:innen mit HNO Infektionen ohne Krankenhausaufenthalt aufgrund von HNO Infektionen innerhalb des Beobachtungsjahres (risikoadjustiert)	Patient:innen ohne einen stationären Krankenhausfall im Beobachtungsjahr aufgrund von ICDs (Hauptdiagnose) der entsprechenden Krankheitsgruppe	Patient:innen mit HNO Infektionen
D11-15	O	Anteil der Patient:innen mit HNO Infektionen mit weniger als zwei Krankenhausaufenthalten aufgrund von HNO Infektionen innerhalb des Beobachtungsjahres (risikoadjustiert)	Patient:innen mit weniger als zwei stationären Krankenhausaufenthalten aufgrund von ICDs (Hauptdiagnose) der Krankheitsgruppe	Patient:innen mit HNO Infektionen
**12 Depressive Störungen**				
D12-1	V	Patient:innen mit depressive Störungen: Anteil der Patient:innen im Verhältnis zu den Netzwerkpatient:innen	Anzahl der gemeinsam behandelten Patient:innen mit mindestens einer der Diagnosen im Netzwerk	
D12-2	V	Patient:innen mit depressive Störungen: Anteil der Patient:innen im Verhältnis zu den Netzwerkpatient:innen	Anzahl der gemeinsam behandelten Patient:innen mit mindestens einer der Diagnosen im Netzwerk	Anzahl der Patient:innen im Netzwerk
D12-3	V	Multimorbiditätsrate Patient:innen mit Depressiven Störungen	Patient:innen der Krankheitsgruppe, die mindestens zwei verschiedene Krankheitsgruppen im Beobachtungsjahr kodiert bekamen	Patient:innen der Krankheitsgruppe
D12-4	G	Mortalitätsrate Patient:innen mit Depressiven Störungen	Patient:innen der Krankheitsgruppe, die im Beobachtungsjahr verstarben	Patient:innen der Krankheitsgruppe
D12-5	G	Rate der Patient:innen der Krankheitsgruppe mit mindestens einem Abrechnungskontakt zu einem/einer Fachärzt:in für (1) Allgemeinmedizin / Praktische/r Ärzt:in (Hausärzt:in) / hausärztliche Innere Medizin (2) Frauenheilkunde (3) Neurologie / Neurochirurgie (4) Psychiatrie und Psychotherapie (5) Neurologie und Psychiatrie (6) Psychosomatische Medizin und Psychotherapie (7) Psychotherapeutisch tätigen Arzt / Psychologischen Psychotherapeut innerhalb des Beobachtungsjahrs	Patient:innen der Krankheitsgruppe, für die mindestens eine Abrechnungsposition von einem / einer Fachärzt:in für (1) – (7) abgerechnet wurde	Patient:innen der Krankheitsgruppe
D12-6	V	Rate der Patient:innen der Krankheitsgruppe mit mindestens einem Abrechnungskontakt zu zwei verschiedenen Fachärzt:innen für (1) – (7) innerhalb des Beobachtungsjahrs	Patient:innen der Krankheitsgruppe, für die mindestens eine Abrechnungsposition von zwei verschiedenen Fachärzt:innen für (1) – (7) unterschiedlicher BSNR und mit der Art der ärztlichen Inanspruchnahme "O" abgerechnet wurde	Patient:innen der Krankheitsgruppe
D12-7	V	Rate der Patient:innen der Krankheitsgruppe mit mindestens einem Abrechnungskontakt zu einem/ Fachärzt:in für (2) – (7) mit Überweisung innerhalb des Beobachtungsjahrs	Patient:innen der Krankheitsgruppe, für die mindestens eine Abrechnungsposition von einem/ Fachärzt:in für (2) – (7) abgerechnet wurde	Patient:innen der Krankheitsgruppe
D12-8	V	Anteil der Patient:innen mit Abrechnungskontakt in mindestens einem von vier Quartalen bei oben genannten Arztgruppen	Patient:innen mit Abrechnungskontakt in mindestens einem von vier Quartalen	Patient:innen der Krankheitsgruppe

Nr.	Kat.*	Indikatorbeschreibung	Zähler	Nenner
D12-9	V	Rate der Patient:innen mit Depressiven Störungen, die im Beobachtungsjahr mindestens einmal ein psychosomatisches Gespräch in Anspruch nahmen	Patient:innen mit Depressiven Störungen, für die ambulant im Beobachtungsjahr mindestens einmal eine der folgenden Abrechnungspositionen verrechnet wurde: EBM 35100 / 35110 / 22221 / 22222	Patient:innen mit Depressiven Störungen
D12-10	V	Rate der Patient:innen mit Depressiven Störungen, die Anxiolytika, Hypnotika oder Sedativa im Beobachtungsjahr verordnet bekamen	Patient:innen mit Depressiven Störungen die Anxiolytika, Hypnotika oder Sedativa (ATC N05B oder N05C) mindestens einmal im Beobachtungsjahr verordnet bekamen	Patient:innen mit Depressiven Störungen
D12-11	V	Rate der Patient:innen mit Depressiven Störungen, die im Beobachtungsjahr Antidepressiva und zugleich Anxiolytika, Hypnotika oder Sedativa verordnet bekamen	Patient:innen mit Depressiven Störungen, die im Beobachtungsjahr Antidepressiva (ATC N06A) und zugleich mindestens einmal Anxiolytika, Hypnotika oder Sedativa (ATC N05B oder N05C) verordnet bekamen	Patient:innen mit Depressiven Störungen, die Antidepressiva (ATC Code N06A) verschrieben bekamen
D12-12	V	Rate der Patient:innen mit Depressiven Störungen mit mindestens mittelschwerer Episode, die im Beobachtungsjahr ein Antidepressivum verordnet bekamen	Patient:innen mit Depressiven Störungen mit mindestens mittelschwerer Depression, die ein Antidepressivum (ATC N06a) verordnet bekamen	Patient:innen mit Depressiven Störungen mit mindestens mittelschwerer Depression definiert aus der Gruppe der Patient:innen mit Depressiven Störungen und ambulant (gesichert) oder stationär (Hauptdiagnose) mindestens eine der folgenden Diagnosen: ICD F32.1 / F32.2 / F32.3 / F33.1 / F33.2 / F33.3 / F33.4
D12-13	V	Rate der Patient:innen mit Depressiven Störungen mit mindestens mittelschwerer Episode, die im Beobachtungsjahr nachdem sie ein Antidepressivum erhielten innerhalb desselben oder des Folgequartals eine weitere Verschreibung für ein Antidepressivum erhielten	Patient:innen mit Depressiven Störungen mit mindestens mittelschwerer Episode, die nachdem sie zum ersten Mal ein Antidepressivum (ATC N06A) erhielten, innerhalb desselben oder des Folgequartals eine weitere Verschreibung für ein Antidepressivum erhielten	Patient:innen mit Depressiven Störungen mit mindestens mittelschwerer Depression definiert als entweder ambulant gesichert oder stationär (Hauptdiagnose) mindestens eine der folgenden Diagnosen: F32.1 / F32.2 / F32.3 / F33.1 / F33.2 / F33.3 / F33.4. Hier ist der Nenner eingegrenzt auf Patient:innen, die im ersten, zweiten oder dritten Quartal eine Verschreibung bekamen.
D12-14	O	Anteil der Patient:innen mit Depressiven Störungen ohne Krankenhausaufenthalt aufgrund von Depressiven Störungen innerhalb des Beobachtungsjahres (risikoadjustiert)	Patient:innen ohne einen stationären Krankenhausfall im Beobachtungsjahr aufgrund von ICDs (Hauptdiagnose) der entsprechenden Krankheitsgruppe	Patient:innen mit Depressiven Störungen
D12-15	O	Anteil der Patient:innen mit Depressiven Störungen mit weniger als zwei Krankenhausaufenthalten aufgrund von Depressiven Störungen innerhalb des Beobachtungsjahres (risikoadjustiert)	Patient:innen mit weniger als zwei stationären Krankenhausaufenthalten aufgrund von ICDs (Hauptdiagnose) der Krankheitsgruppe	Patient:innen mit Depressiven Störungen
**13 Diabetes mellitus**				
D13-1	V	Diabetes mellitus Typ-1: Anzahl der gemeinsam behandelten Patient:innen mit mindestens einer der Diagnosen im Netzwerk	Diabetes Patient:innen, im Netzwerk definiert als Diabetes Patient:innen, mit stationärer Hauptdiagnose oder ambulant gesicherter E10.* Diagnose	

Nr.	Kat.*	Indikatorbeschreibung	Zähler	Nenner
D13-2	V	Diabetes mellitus Typ-2: Anzahl der gemeinsam behandelten Patient:innen mit mindestens einer der Diagnosen im Netzwerk	Diabetes Patient:innen, definiert als Diabetes Patient:innen, mit stationärer Hauptdiagnose oder ambulant gesicherter E11.* Diagnose	
D13-3	V	Patient:innen mit Diabetes mellitus Typ-1: Anteil der Patient:innen im Verhältnis zu den Netzwerkpatient:innen	Diabetes Patient:innen, definiert als Diabetes Patient:innen, mit stationärer Hauptdiagnose oder ambulant gesicherter E10* Diagnose	Patient:innen im Netzwerk
D13-4	V	Patient:innen mit Diabetes mellitus Typ-2: Anteil der Patient:innen im Verhältnis zu den Netzwerkpatient:innen	Diabetes Patient:innen, definiert als Diabetes Patient:innen, mit stationärer Hauptdiagnose oder ambulant gesicherter E11.* Diagnose	Patient:innen im Netzwerk
D13-5	G	Multimorbiditätsrate Diabetes mellitus Typ-2 Patient:innen	Patient:innen der Krankheitsgruppe und Diabetes mellitus Typ-2 Patient:innen, die mindestens zwei verschiedene Krankheitsgruppen im Beobachtungsjahr kodiert bekamen	Diabetes Patient:innen Typ-2
D13-6	G	Multimorbiditätsrate Diabetes mellitus Typ-1 Patient:innen	Patient:innen der Krankheitsgruppe und Diabetes mellitus Typ-1 Patient:innen, die mindestens zwei verschiedene Krankheitsgruppen im Beobachtungsjahr kodiert bekamen	Diabetes Patient:innen Typ-1
D13-7	V	Rate der Diabetes Patient:innen (Diabetes mellitus Typ-1), die im Beobachtungsjahr im DM1-DMP eingeschrieben waren	Diabetes Patient:innen Typ-1, die im Beobachtungszeitraum im DM1-DMP Programm eingeschrieben waren	Diabetes Patient:innen Typ-1
D13-8	V	Rate der Diabetes Patient:innen (Diabetes mellitus Typ-2), die im Beobachtungsjahr im DM2-DMP eingeschrieben waren	Diabetes Patient:innen Typ-2, die im Beobachtungszeitraum im DM2-DMP Programm eingeschrieben waren	Diabetes Patient:innen Typ-2
D13-9	G	Mortalitätsrate Diabetes mellitus Typ-1 Patient:innen	Diabetes Patient:innen Typ-1, die im Beobachtungsjahr verstarben	Diabetes Patient:innen Typ-1
D13-10	G	Mortalitätsrate Diabetes mellitus Typ-2 Patient:innen	Diabetes Patient:innen Typ-2, die im Beobachtungsjahr verstarben	Diabetes Patient:innen Typ-2
D13-11	V	Rate der Patient:innen mit Diabetes mellitus Typ-1 mit mindestens einem Abrechnungskontakt zu einem/einer (1) Fachärzt:in für Allgemeinmedizin / Praktische/r Ärzt:in (Hausärzt:in) / hausärztliche Innere Medizin (2) Nephrologie (3) diabetologischen Schwerpunktpraxis (4) Augenarzt innerhalb des Beobachtungsjahrs	Diabetes Patient:innen Typ-1, für die mindestens eine Abrechnungsposition von einem / einer Fachärzt:in für (1) – (4) abgerechnet wurde	Diabetes Patient:innen Typ-1
D13-12	V	Rate der Patient:innen mit Diabetes mellitus Typ-2 mit mindestens einem Abrechnungskontakt zu einem/einer Fachärzt:in für Allgemeinmedizin / Praktische/r Ärzt:in (Hausärzt:in) / hausärztliche Innere Medizin innerhalb des Beobachtungsjahrs	Diabetes Patient:innen Typ-2, für die mindestens eine Abrechnungsposition von einem / einer Fachärzt:in für (1) – (4) abgerechnet wurde	Diabetes Patient:innen Typ-2
D13-13	V	Rate der Patient:innen mit Diabetes mellitus Typ-1 mit mindestens einem Abrechnungskontakt zu zwei verschiedenen Fachärzt:innen für (1) – (4) innerhalb des Beobachtungsjahrs	Diabetes Patient:innen Typ-1, für die mindestens eine Abrechnungsposition von zwei verschiedenen Fachärzt:innen für (1) – (4) unterschiedlicher BSNR und mit der Art der ärztlichen Inanspruchnahme "O" abgerechnet wurde	Diabetes Patient:innen Typ-1

Nr.	Kat.*	Indikatorbeschreibung	Zähler	Nenner
D13-14	V	Rate der Patient:innen mit Diabetes mellitus Typ-2 mit mindestens einem Abrechnungskontakt zu zwei verschiedenen Fachärzt:innen für (1) – (4) innerhalb des Beobachtungsjahrs	Diabetes Patient:innen Typ-2, für die mindestens eine Abrechnungsposition von zwei verschiedenen Fachärzt:innen für (1) – (4) unterschiedlicher BSNR und mit der Art der ärztlichen Inanspruchnahme "O" abgerechnet wurde	Diabetes Patient:innen Typ-2
D13-15	V	Rate der Patient:innen mit Diabetes mellitus Typ-1, die mindestens zweimal im Beobachtungsjahr ambulanten Kontakt zu einem/einer Hausärzt:in oder Internist:in hatte	Diabetes Patient:innen Typ-1, bei denen mindestens zweimal eine Abrechnung von einem/einer Fachärzt:in der folgenden Facharztgruppen erfolgte: Allgemeinmediziner:in (Hausärzt:in), Ärzt:in / Praktische/r Ärzt:in (Hausärzt:in), Internist:in (Hausärzt:in), Internist:in)	Diabetes Patient:innen Typ-1
D13-16	V	Rate der Patient:innen mit Diabetes Typ-2, die mindestens zweimal im Beobachtungsjahr ambulanten Kontakt zu einem/einer Hausärzt:in oder Internist:in hatte	Diabetes Patient:innen Typ-2, bei denen mindestens zweimal eine Abrechnung von einem/einer Fachärzt:in der folgenden Facharztgruppen erfolgte: Allgemeinmediziner:in (Hausärzt:in), Ärzt:in / Praktische/r Ärzt:in (Hausärzt:in), Internist:in (Hausärzt:in), Internist:in)	Diabetes Patient:innen Typ-2
D13-17	V	Rate der Patient:innen mit Diabetes mellitus Typ-1 mit mindestens einem Abrechnungskontakt zu einem/einer Fachärzt:in für (2)- (4) mit Überweisung innerhalb des Beobachtungsjahrs	Diabetes mellitus Typ-1 Patient:innen, für die mindestens eine Abrechnungsposition von einem/einer Fachärzt:in für (2) – (4) mit Überweisung abgerechnet wurde	Diabetes Patient:innen Typ-1
D13-18	V	Rate der Patient:innen mit Diabetes mellitus Typ-2 mit mindestens einem Abrechnungskontakt zu einem/einer Fachärzt:in für (2) – (4) mit Überweisung innerhalb des Beobachtungsjahrs	Diabetes Typ-2 Patient:innen, für die mindestens eine Abrechnungsposition von einem/einer Fachärzt:in für (2) – (4) mit Überweisung abgerechnet wurde	Diabetes Patient:innen Typ-2
D13-19	V	Anteil der Patient:innen mit Diabetes mellitus Typ-1 mit Abrechnungskontakt in mindestens einem von vier Quartalen bei oben genannten Arztgruppen	Diabetes Typ-1 Patient:innen mit Abrechnungskontakt in mindestens einem von vier Quartalen	Diabetes Patient:innen Typ-1
D13-20	V	Anteil der Patient:innen mit Diabetes mellitus Typ-2 mit Abrechnungskontakt in mindestens einem von vier Quartalen bei oben genannten Arztgruppen	Diabetes Typ-2 Patient:innen mit Abrechnungskontakt in mindestens einem von vier Quartalen	Diabetes Patient:innen Typ-2
D13-21	V	Rate der Diabetes mellitus Typ-1 Patient:innen, die im Beobachtungsjahr an einer DMP Schulung teilnahmen	Diabetes Patient:innen Typ-1, für die eine Schulung abgerechnet wurde (EBM 91114E/N/W (KV WL) / 91118E/N/W (KVWL) / 91120 E/N/W (KVWL) / 91122(W) (KVWL) / 91124(E/N/W) (KVWL) / 91126 (KVWL) / 91128(N/W) (KVWL) / 90307A (KVNO) / 98009A (KVNO) / 98016A (KVNO) / 98017A (KVNO) / 98018A (KVNO) / 99435 (KVHH) / 99436 (KVHH) 97006 (KVHH) / 97008 (KVHH) / 97011 (KVHH) / 97014 (KVHH) / 97016 (KVHH) / 97018 (KVHH) / 97023 (KVHH) / 97025 (KVHH) / 97027 (KVHH) / 99746A (KVSH) / 99746B (KVSH) / 99746C (KVSH) / 99746D (KVSH) / 99746F (KVSH) / 99746G (KVSH) / 99747A (KVSH) / 99748B (KVSH) / 99749A (KVSH) / 99749B (KVSH) / 99749C (KVSH) / 99749D (KVSH) /	Diabetes Typ-1 Patient:innen mit DMP Einschreibung im Beobachtungsjahr

Nr.	Kat.*	Indikatorbeschreibung	Zähler	Nenner
			99737A (KVSH) / 99737B (KVSH) / 99749F (KVSH) / 99749G (KVSH))	
D13-22	V	Rate der Diabetes Typ-2 Patient:innen, die im Beobachtungsjahr an einer DMP Schulung teilnahmen	Diabetes Patient:innen Typ-2, für die eine Schulung abgerechnet wurde (90244 (E, N, W) (KVWL) / 90246 (E, N, W) (KVWL) / 90247 (T, E, N, W) (KVWL) / 90249 (E, N, W) (KVWL) / 90251 (E, N, W) (KVWL) / 90252 (E, N, W) (KVWL) / 90254 (E, N, W) (KVWL) / 90255 (E, N, W) (KVWL) / 90256 (E, N, W) (KVWL) / 90258 (W) (KVWL) / 90260 (N, W) (KVWL) / 90265 (E, N, W) (KVWL) / 90267 (E, N, W) (KVWL) / 90269 (E, N, W) (KVWL) / 90272 (E, N, W) (KVWL) / 90274 (E, N, W) (KVWL) / 90276 (E, N, W) (KVWL) / 90278 (E, N, W) (KVWL) / 98013 (KV NO) / 98014 (KV NO) / 98016 (KV NO) / 98017 (KV NO) / 98018 (KV NO) / 98022 (KV NO) / 98023 (KV NO) / 98024 (KV NO) / 99436 (KV HH)/ 99195 (KVHH) / 99196 (KVHH) / 99197 (KVHH) / 99198 (KVHH) / 99199 (KVHH) / 99200 (KVHH) / 99215 (KVHH) / 99216 (KVHH) / 99217 (KVHH) / 99218 (KVHH) / 99219 (KVHH) / 99220 (KVHH) / 99221 (KVHH) / 99222 (KVHH) / 99762(A,B) (KVSH) / 99791(D,E,F,G,H,I,J,K,L,M,N,O) (KVSH) / 99759 (KVSH) / 99761 (KVSH) / 99762 (KVSH) / 99762(C,D) (KVSH) / 99765 (KVSH) / 99768 (KVSH) / 99771 (KVSH) / 99773 (KVSH) / 99774 (KVSH) / 99775 (KVSH) / 99776 (KVSH) / 99777 (KVSH) / 99778 (KVSH) / 99783 (KVSH)	Diabetes Typ-2 Patient:innen mit DMP Einschreibung im Beobachtungsjahr
D13-23	V	Rate der Patient:innen mit Diabetes mellitus Typ-1, die eine Grippeimpfung im Beobachtungsjahr erhielten	Diabetes Patient:innen Typ-1I, die im Beobachtungsjahr mindestens eine Grippeimpfung (EBM 89111 / 89112 oder ATC J07BB) erhielten	Diabetes Patient:innen Typ-1I
D13-24	V	Rate der Patient:innen mit Diabetes mellitus Typ-2, die eine Grippeimpfung im Beobachtungsjahr erhielten	Diabetes Patient:innen Typ-2, die im Beobachtungsjahr mindestens eine Grippeimpfung (EBM 89111 / 89112 oder ATC J07BB) erhielten	Diabetes Patient:innen Typ-2
D13-25	V	Rate der Patient:innen mit Diabetes mellitus Typ-1, bei denen im Beobachtungsjahr eine Augenhintergrunduntersuchung durchgeführt wurde	Diabetes Patient:innen Typ-1, für die im Beobachtungsjahr mindestens einmal ambulant eine Augenhintergrunduntersuchung 06333 (90770 (KVWL) / 90311 (KVNO) / 97022 (KVHH) / 99750 (KVSH)) abgerechnet wurde	Diabetes Patient:innen Typ-1
D13-26	V	Rate der Patient:innen mit Diabetes mellitus Typ-2, bei denen im Beobachtungsjahr eine Augenhintergrunduntersuchung durchgeführt wurde	Diabetes Patient:innen Typ-2, für die im Beobachtungsjahr mindestens einmal ambulant eine Augenhintergrunduntersuchung 06333 (90770 (KVWL) / 90311 (KVNO) / 97022 (KVHH) / 99750 (KVSH)) abgerechnet wurde	Diabetes Patient:innen Typ-2
D13-27	V	Rate der Diabetes mellitus Typ-1 Patient:innen mit mindestens einem HDL Test im Beobachtungsjahr	Diabetes Patient:innen Typ-1, die mindestens einen HDL (EBM 32061) im Beobachtungsjahr abgerechnet bekamen	Diabetes Patient:innen Typ-1

Nr.	Kat.*	Indikatorbeschreibung	Zähler	Nenner
D13-28	V	Rate der Diabetes mellitus Typ-2 Patient:innen mit mindestens einem HDL Test im Beobachtungsjahr	Diabetes Patient:innen Typ-2, die im Beobachtungsjahr mindestens einen HDL (EBM 32061) abgerechnet bekamen	Diabetes Patient:innen Typ-2
D13-29	V	Rate der Diabetes mellitus Typ-1 Patient:innen mit einer Hba1c Bestimmung ambulant im Beobachtungsjahr	Diabetes Patient:innen Typ-1, für die mindestens einmal ambulant eine Hba1c Bestimmung abgerechnet wurde (EBM 32094 / 90310 (KVNO) / 90321 (KVNO))	Diabetes Patient:innen Typ-1
D13-30	V	Rate der Diabetes mellitus Typ-2 Patient:innen mit einer Hba1c Bestimmung ambulant im Beobachtungsjahr	Diabetes Patient:innen Typ-2, für die mindestens einmal ambulant eine Hba1c Bestimmung abgerechnet wurde (EBM 32094 / 90310 (KVNO) / 90321 (KVNO))	Diabetes Patient:innen Typ-2
D13-31	V	Rate der Diabetes mellitus Typ-1 Patient:innen mit mindestens zwei HbA1c Bestimmungen und mindestens einem LDL Test im Beobachtungsjahr	Diabetes Typ-1 Patient:innen, die mindestens zweimal einen HbA1c Test (EBM 32094 / 90310 (KVNO) / 90321 (KVNO)) und mindestens einen LDL Test (EBM 32062) im Beobachtungsjahr abgerechnet bekamen	Diabetes Patient:innen Typ-1
D13-32	V	Rate der Diabetes Typ-2 Patient:innen, die im Beobachtungsjahr mindestens zwei HbA1c Bestimmungen und mindestens einen LDL Test erhielten	Diabetes Typ-2 Patient:innen, die mindestens zweimal einen HbA1c Test (EBM 32094 / 90310 (KVNO) / 90321 (KVNO)) und mindestens einen LDL Test (EBM 32062) im Beobachtungsjahr abgerechnet bekamen	Diabetes Patient:innen Typ-2
D13-33	V	Rate der Diabetes mellitus Typ-1 Patient:innen mit Verschreibung von oralen Antidiabetika im Beobachtungsjahr	Diabetes Patient:innen Typ-1 mit Verschreibung von oralen Antidiabetika (ATC A10B) im Beobachtungszeitraum	Diabetes Patient:innen Typ-1
D13-34	V	Anteil der KHK Patient:innen mit Diabetes Typ II, die im Beobachtungsjahr Lipidsenker verordnet bekamen	KHK Patient:innen, die auch Diabetes Typ 2 Patient:innen sind und mindestens einmal im Beobachtungsjahr Lipidsenker (ATC C10A / C10B) verordnet bekamen	KHK Patient:innen mit mindestens 2 ambulanten gesicherten oder einer stationären E11.* Diagnose/n
D13-35	V	Rate der Diabetes Typ-2 Patient:innen mit Verschreibung von oralen Antidiabetika im Beobachtungsjahr	Diabetes Patient:innen Typ-2 mit Verschreibung von oralen Antidiabetika (ATC A10B) im Beobachtungszeitraum	Diabetes Patient:innen Typ-2
D13-36	V	Rate der Patient:innen mit Diabetes mellitus Typ-2 mit Metformin Verschreibung im Beobachtungsjahr	Diabetes Typ-2 Patient:innen mit Verschreibung von Metformin (ATC A10BA02 oder A10BD*) im Beobachtungsjahr	Diabetes Patient:innen Typ-2
D13-37	V	Rate der Diabetes mellitus Typ-1 Patient:innen mit Verschreibung von Insulin oder Analoga im Beobachtungsjahr	Patient:innen Typ-1 mit Verschreibung von Insulin oder Analoga (ATC A10A* und / oder A10B*) im Beobachtungszeitraum	Diabetes Patient:innen Typ-1
D13-38	V	Rate der Diabetes Typ-2 Patient:innen mit Verschreibung von Insulin oder Analoga im Beobachtungsjahr	Patient:innen Typ-2 mit Verschreibung von Insulin oder Analoga (ATC A10A* und / oder A10B*) im Beobachtungszeitraum	Diabetes Patient:innen Typ-2
D13-39	O	Rate der Diabetes mellitus Typ-1 Patient:innen mit mindestens einem Myokardinfarkt innerhalb des Beobachtungsjahres	Diabetes Patient:innen Typ-1, die einen Myokardinfarkt (stationäre Haupt- oder Nebendiagnose ICD I21*, I22*, I23*) im Beobachtungsjahr diagnostiziert bekamen	Diabetes Typ-1 Patient:innen
D13-40	O	Rate der Diabetes mellitus Typ-2 Patient:innen mit mindestens einem Myokardinfarkt innerhalb des Beobachtungsjahres	Diabetes Patient:innen Typ-2, die einen Myokardinfarkt (stationäre Haupt- oder Nebendiagnose ICD I21*, I22*, I23*) im Beobachtungsjahr diagnostiziert bekamen	Diabetes Typ-2 Patient:innen

Nr.	Kat.*	Indikatorbeschreibung	Zähler	Nenner
D13-41	O	Anteil der Diabetes mellitus Typ-1 Patient:innen ohne Krankenhausaufenthalt aufgrund von Diabetes innerhalb des Beobachtungsjahres (risikoadjustiert)	Diabetes Patient:innen Typ-1 ohne stationären Krankenhausfall im Beobachtungsjahr aufgrund von ICDs (Hauptdiagnose) der entsprechenden Diagnoseuntergruppe Diabetes Typ-1	Diabetes Typ-1 Patient:innen
D13-42	O	Anteil der Diabetes mellitus Typ-2 Patient:innen mit weniger als zwei Krankenhausaufenthalten aufgrund von Diabetes innerhalb des Beobachtungsjahres (risikoadjustiert)	Diabetes Patient:innen Typ-2 mit weniger als zwei stationären Krankenhausaufenthalten aufgrund von ICDs (Hauptdiagnose) der Diagnoseuntergruppe Diabetes Typ-2	Diabetes Typ-2 Patient:innen
D13-43	O	Anteil der Diabetes mellitus Typ-1 Patient:innen mit weniger als zwei Krankenhausaufenthalten aufgrund von Diabetes innerhalb des Beobachtungsjahres (risikoadjustiert)	Diabetes Patient:innen Typ-1 mit weniger als zwei stationären Krankenhausaufenthalten aufgrund von ICDs (Hauptdiagnose) der Diagnoseuntergruppe Diabetes Typ-1	Diabetes Typ-1 Patient:innen
D13-44	O	Anteil der Diabetes mellitus Typ-2 Patient:innen ohne Krankenhausaufenthalt aufgrund von Diabetes innerhalb des Beobachtungsjahres (risikoadjustiert)	Diabetes Patient:innen Typ-2 ohne stationären Krankenhausfall im Beobachtungsjahr aufgrund von ICDs (Hauptdiagnose) der entsprechenden Diagnoseuntergruppe Typ-2	Diabetes Typ-2 Patient:innen
**14 Gonarthrose**				
D14-1	V	Gonarthrose: Anzahl der gemeinsam behandelten Patient:innen mit mindestens einer der Diagnosen im Netzwerk	Anzahl der gemeinsam behandelten Patient:innen mit mindestens einer der Diagnosen im Netzwerk	
D14-2	V	Patient:innen mit Gonarthrose: Anteil der Patient:innen im Verhältnis zu den Netzwerkpatient:innen	Anzahl der gemeinsam behandelten Patient:innen mit mindestens einer der Diagnosen im Netzwerk	Anzahl der Patient:innen im Netzwerk
D14-3	G	Multimorbiditätsrate Gonarthrose Patient:innen	Patient:innen der Krankheitsgruppe, die mindestens zwei verschiedene Krankheitsgruppen im Beobachtungsjahr kodiert bekamen	Patient:innen der Krankheitsgruppe
D14-4	G	Mortalitätsrate Gonarthrose Patient:innen	Patient:innen der Krankheitsgruppe, die im Beobachtungsjahr verstarben	Patient:innen der Krankheitsgruppe
D14-5	V	Rate der Patient:innen der Krankheitsgruppe mit mindestens einem Abrechnungskontakt zu einem/einer Fachärzt:in für (1) Allgemeinmedizin / Praktische/r Ärzt:in (Hausärzt:in) / hausärztliche Innere Medizin (2) Anästhesiologie (3) Chirurgie (4) Rheumatologie (der Inneren Medizin) (5) Orthopäden / Unfallchirurgen (6) Psychosomatische Medizin und Psychotherapie (7) Radiologie (8) Physikalische und Rehabilitative Medizin innerhalb des Beobachtungsjahrs	Patient:innen der Krankheitsgruppe, für die mindestens eine Abrechnungsposition von einem / einer Fachärzt:in für (1) – (8) abgerechnet wurde	Patient:innen der Krankheitsgruppe
D14-6	V	Rate der Patient:innen der Krankheitsgruppe mit mindestens einem Abrechnungskontakt zu zwei verschiedenen Fachärzt:innen für (1) – (8) in (Hausärzt:in) / hausärztliche Innere Medizin innerhalb des Beobachtungsjahrs	Patient:innen der Krankheitsgruppe, für die mindestens eine Abrechnungsposition von zwei verschiedenen Fachärzt:innen für (1) – (8) unterschiedlicher BSNR und mit der Art der ärztlichen Inanspruchnahme "O" im Beobachtungsjahr abgerechnet wurde	Patient:innen der Krankheitsgruppe
D14-7	V	Rate der Patient:innen der Krankheitsgruppe mit mindestens einem Abrechnungskontakt zu einem/ Fachärzt:in für (2) – (8) mit Überweisung innerhalb des Beobachtungsjahrs	Patient:innen der Krankheitsgruppe, für die mindestens eine Abrechnungsposition von einem/ Fachärzt:in für (2) – (8) abgerechnet wurde	Patient:innen der Krankheitsgruppe

Nr.	Kat.*	Indikatorbeschreibung	Zähler	Nenner
D14-8	V	Anteil der Patient:innen mit Abrechnungskontakt in mindestens einem von vier Quartalen bei oben genannten Arztgruppen	Patient:innen mit Abrechnungskontakt in mindestens einem von vier Quartalen	Patient:innen der Krankheitsgruppe
D14-9	V	Rate der Gonarthrose Patient:innen, die innerhalb des Beobachtungsjahrs keine Opioide verordnet bekamen	Gonarthrose Patient:innen, die nicht mindestens einmal im Beobachtungsjahr ATC N02A verordnet bekamen	Gonarthrose Patient:innen
D14-10	O	Anteil der Gonarthrose Patient:innen ohne Krankenhausaufenthalt aufgrund von Gonarthrose innerhalb des Beobachtungsjahres (risikoadjustiert)	Patient:innen ohne einen stationären Krankenhausfall im Beobachtungsjahr aufgrund von ICDs (Hauptdiagnose) der entsprechenden Krankheitsgruppe	Gonarthrose Patient:innen
D14-11	V	Anzahl der Patient:innen mit Gonarthrose, die im Beobachtungszeitraum eine Knieendoprothese eingesetzt bekamen	Gonarthrose Patient:innen mit stationärem Aufenthalt und der Prozedur OPS 5-822 oder 5-823	Gonarthrose Patient:innen
D14-12	O	Rate der Gonarthrose Patient:innen mit Einsatz einer Knieendoprothese, die innerhalb von 6 Monaten keine Thrombose oder Lungenembolie hatten	Gonarthrose Patient:innen, die im selben oder in einem der zwei folgenden Quartale nach einer Knieendoprothese (OPS 5-822) keine Thrombose hatten (ambulant G oder Z, Krankenhaus stationär und Krankenhaus ambulant Hauptdiagnose: ICD I80.1 / I80.2 / I80.3 / 182.2 / I26*)	Gonarthrose Patient:innen mit Einsatz einer Knieendoprothese (OPS 5-822). Berücksichtigt sind nur Patient:innen mit Indexevent in Quartal 1 und 2.
D14-13	O	Anteil der Gonarthrose Patient:innen mit weniger als zwei Krankenhausaufenthalten aufgrund von Gonarthrose innerhalb des Beobachtungsjahres (risikoadjustiert)	Patient:innen mit weniger als zwei stationären Krankenhausaufenthalten aufgrund von ICDs (Hauptdiagnose) der Krankheitsgruppe	Gonarthrose Patient:innen

* Kategorien der Indikatoren: G Patientencharakteristika, V – Informationen Patientenpfaden, O - Ergebnisindikatoren

A5 –Table of excluded indicators

Disease	Indikator	Exclusion reason
Ischaemic heart diseases	Share of CHD patients receiving antiplatelet agents	Redundant indicator - already depicted in an included indicator
	Share of patients in whom secondary prevention of cardiovascular events was used and aspirin or antiplatelet agents were prescribed	Not fully depictable in routine data
	Share of patients with ischemic vascular disease in whom aspirin or another antithrombotic was used	Not fully depictable in routine data
	Share of patients who received antiplatelet agents after stent implantation	Not fully depictable in routine data
	Share of patients with stable coronary artery disease who are prescribed aspirin and anti-atherosclerotic medications	Not fully depictable in routine data
	Share of patients with cardiovascular disease receiving annual influenza vaccination	Not fully depictable in observation time
	Share of patients with stable coronary artery disease who were vaccinated against pneumonia	Not fully depictable in routine data
	Share of patients with a diagnosis of stable coronary artery disease and chronic kidney disease who are prescribed an ACE inhibitor or ARB	Modification of the indicator required
	Share of patients with stable coronary artery disease and hypertension who were prescribed an ACE inhibitor or ARB	Indicator is subject to a specific subgroup
	Share of coronary artery disease patients who were prescribed antiplatelet therapy	Redundant indicator - already depicted in an included indicator
	Share of patients with coronary artery disease and prior myocardial infarction prescribed beta-blocker	Redundant indicator - already depicted in an included indicator
	Share of patients with coronary artery disease who were prescribed a lipid-lowering therapy	Redundant indicator - already depicted in an included indicator
	Share of patients discharged for AMI, CABG, or PTCA who took aspirin or another antithrombotic during the one year observation period	Not fully depictable in routine data
	Share of patients who had a myocardial infarction and who are currently being treated with ACE-I or ARB if ACE-I intolerant, dual anti-platelet therapy, beta-blocker and a statin	Redundant indicator - already depicted in an included indicator
	Share of patients with a history of myocardial infarction who are currently being treated with an ACE-I or ARB if ACE-I intolerant, aspirin or anticoagulant drug therapy and a statin and a beta-blocker for those patients with left ventricular systolic dysfunction	Not fully depictable in routine data
	The share of patients with coronary heart disease with a prescription for aspirin, an alternative anti-platelet therapy, or an anti-coagulant	Not fully depictable in routine data
	Share of patients with coronary heart disease who have had influenza immunisation in the last year	Redundant indicator - already depicted in an included indicator
	Share of patients with ischemic vascular diseases that use aspirin or another antithrombotic	Not fully depictable in routine data
	Share of patients who had an acute myocardial infarction and are prescribed a beta-blocker	Redundant indicator - already depicted in an included indicator
	Share of patients with coronary artery disease who are prescribed an antiplatelet/anticoagulant drug	Redundant indicator - already depicted in an included indicator
	Share of diabetes patients with a history of diabetes and not on lipid lowering drug	Redundant indicator - already depicted in an included indicator
	Share of diabetes patients with cardiovascular disease that receive appropriate treatment (beta-blocking agents, ACE-I, anti platelet therapy, lipid lowering therapy, long acting calcium channel antagonist)	Redundant indicator - already depicted in an included indicator

Disease	Indikator	Exclusion reason
Ischaemic heart diseases	Share of diabetes patients receiving intensive insulin treatment after acute MI.	Not fully depictable in routine data
	Share of diabetes patients with at least one CHD-related event (death, acute MI. coronary syndromes, and angina)	Not fully depictable in routine data
	Share of patients with coronary heart disease who had a least one LDL test within the observation year	Redundant indicator - already depicted in an included indicator
	Share of patients with coronary heart disease who had a least one HDL test within the observation year	Redundant indicator - already depicted in an included indicator
	Share of patients with coronary heart disease who had a prescription fill for a statin or fibrate within the observation year	No clinical evidence for the indicator
	Share of patients with atrial fibrillation, receiving anticoagulants/antiplatelets within the observation year	Redundant indicator - already depicted in an included indicator
	Share of CHD patients with at least one prescription for a lipid-lowering agent within the observation year	Redundant indicator - already depicted in an included indicator
	Share of atherosclerosis patients with prescription of lipid-lowering agents within the observation year	Redundant indicator - already depicted in an included indicator
	Share of patients with diabetes and/or coronary artery disease screened for depression	Not fully depictable in routine data
	Share of CHD patients with diabetes and/or left ventricular systolic dysfunction who received ACE-I or ARB therapy	Redundant indicator - already depicted in an included indicator
	Share of patients with coronary heart disease who have an influenza immunisation	Redundant indicator - already depicted in an included indicator
	Share of patients with coronary artery disease with a prescription of an antiplatelet therapy	Not fully depictable in routine data
	Share of patients undergoing PCI (percutaneous coronary intervention) with major adverse cardiac and cerebrovascular events within 7 days	Wrong focus of indicator
	Share of CHD patients doing rehabilitation sports	Data availability
	Rate of CHD patients prescribed at least one nicotine replacement during the observation year.	Not fully depictable in routine data
Heart failure	Share of patients with heart failure prescribed ACE inhibitors (ACEI) or angiotensin receptor blockers (ARB)	Redundant indicator - already depicted in an included indicator
	Rate of patients with heart failure and a beta blocker prescription	Redundant indicator - already depicted in an included indicator
	Among of patients with heart failure due to left ventricular systolic dysfunction who are treated with an ACE-I or ARB, the Share of patients who are additionally currently treated with a beta-blocker licensed for heart failure	Redundant indicator - already depicted in an included indicator
	Share of primary health care patients with CHF who are taking ACE inhibitors or ARBs	Not fully depictable in routine data
	Share of patients with heart failure due to left ventricular dysfunction on ACE inhibitor therapy	Redundant indicator - already depicted in an included indicator
	Share of patients hospitalised for heart failure who were being treated with both Renin angiotensin aldosterone system inhibitors and beta blockers after 12–18 months	Not fully depictable in routine data
	Share of patients hospitalized for heart failure who had a follow-up contact within 4 weeks of discharge	Redundant indicator - already depicted in an included indicator
	Share of patients with heart failure prescribed an ACE inhibitor or ARB within 1 year	Redundant indicator - already depicted in an included indicator
	Share of patients with heart failure prescribed a beta-blocker within 1 year	Redundant indicator - already depicted in an included indicator
	Share of patients with a current diagnosis of heart failure due to left ventricular systolic dysfunction, who are treated with an ACE-I or ARB	Indicator is subject to a specific subgroup

Disease	Indikator	Exclusion reason
Heart failure	Share of patients with atrial fibrillation with a prescription for warfarin therapy	Redundant indicator - already depicted in an included indicator
	Share of heart failure patients doing rehabilitation sports	Data availability
	Rate of heart failure patients prescribed at least one nicotine replacement during the observation year.	Not fully depictable in routine data
Other diseases of the circulatory system	Share of patients with peripheral arterial disease with a record in the preceding 15 months that aspirin or an alternative anti-platelet is being taken	Not fully depictable in routine data
Bronchitis & COPD	Share of asthma and COPD patients with non-beneficial combination of inhalation medications	Not focussed on an included disease
	Share of patients with COPD or asthma with a hospital stay	Redundant indicator - already depicted in an included indicator
	Share of patients receiving pharmacotherapy for COPD exacerbations	Not fully depictable in routine data
	Share of patients with COPD exacerbations in whom a long-acting bronchodilator was used	Not fully depictable in routine data
	Share of COPD patients requiring hospitalization for COPD-related exacerbations within one month	Redundant indicator - already depicted in an included indicator
	Share of patients aged 18-75 years with acute bronchitis/bronchiolitis who were prescribed antibiotics for systemic use and received the recommended antibiotics	Modification of the indicator required, not fully ascertainable in routine data
	Share of patients with acute bronchitis/bronchiolitis who were prescribed antibiotics for systemic use and received quinolones	Redundant indicator - already depicted in an included indicator
	Share of patients with COPD who have had influenza immunisation in the last year	Redundant indicator - already depicted in an included indicator
	Share of patients with an outpatient or emergency department visit with a diagnosis of acute bronchitis who were not prescribed or dispensed antibiotics on or within 3 days of the initial date of service during the observation year	Redundant indicator - already depicted in an included indicator
	Share of patients with acute exacerbation of chronic obstructive pulmonary disease with antibiotics	Not fullly depictable in routine data
	Share of patients with acute bronchitis who were not prescribed an antibiotic	Redundant indicator - already depicted in an included indicator
	Share of patients with acute exacerbation of COPD treated with broad-spectrum penicillin and / or clavulanic acid	Redundant indicator - already depicted in an included indicator
	Share of patients with acute exacerbation of COPD treated with macrolides	Redundant indicator - already depicted in an included indicator
	Share of patients with COPD treated with quinolones	Redundant indicator - already depicted in an included indicator
	Rate of COPD patients prescribed at least one nicotine replacement during the observation year.	Not fully depictable in routine data
Mental and behavioural disorders due to use of alcohol or opioids	Share of individuals with alcohol dependence with at least one prescription for appropriate pharmacotherapy during the observation year	Not fully depictable in routine data
	Share of individuals with opioid dependence with at least one prescription for appropriate pharmacotherapy during the oberservation year	Indicator is subject to a specific subgroup

Disease	Indikator	Exclusion reason
Back pain [dorsopathies]	Share of patients with back pain receiving injections of nonsteroidal anti-inflammatory drugs	Not fully depictable in routine data
	Share of patients on intravenous medications in patients with low back pain	Not fully depictable in routine data
	Share of patients prescribed the following medications for acute back pain: Phenylbutazone, dexamethasone, other oral steroids, colchicine, and/or antidepressants	Modification in line with national medical guidelines
Hypertension	Share of diabetic patients with hypertension receiving appropriate antihypertensive treatment (beta-blocker, low-dose thiazide, ACE-I inhibitor, angiotensin II receptor antagonist, long-acting calcium channel antagonist).	Redundant indicator - already depicted in an included indicator
	Share of diabetes patients with hypertension with a ACD inhibitor or ARB prescription within the observation year	Redundant indicator - already depicted in an included indicator
	Share of patients with hypertension with at least one antiplatelet drug prescription within the observation year	Not fully depictable in routine data
	Share of patients with diabetes who receive an ACEI or ARB or DRI or ACEI/ARB/DRI Combination during the observation year	Redundant indicator - already depicted in an included indicator
	Share of diabetes patients with diabetic nephropathy and hypertension treated with ACE inhibitors or AT1 receptor antagonists.	Modification of the indicator required
	Share of hypertension patients with prescription of antihypertensive drugs	Redundant indicator - already depicted in an included indicator
Gastroenteritis and other	Share of patients with chronic constipation or impaction with two or more agents with low-to-moderate anticholinergic activity or use of a highly anticholinergic agent	No evidence for the indicator
Influenza and pneumonia	Share of patients hospitalized for bacterial pneumonia	Redundant indicator - already depicted in an included indicator
	Share of patients with pneumonia with narrow-spectrum penicillin	Redundant indicator - already depicted in an included indicator
	Share of patients with pneumonia treated with broad-spectrum penicillin and / or clavulanic acid	Redundant indicator - already depicted in an included indicator
	Share of patients with pneumonia treated with macrolides	Redundant indicator - already depicted in an included indicator
	Share of patients with pneumonia treated with cephalosporins	Redundant indicator - already depicted in an included indicator
	Share of patients with pneumonia treated with quinolones	Redundant indicator - already depicted in an included indicator
	Share of flu and influenza patients with flu vaccination	Redundant indicator - already depicted in an included indicator
Ear nose throat infections	Share of patients diagnosed with viral upper respiratory tract infection who were not prescribed antibiotics	Redundant indicator - already depicted in an included indicator
	Share of patients diagnosed with seasonal allergic rhinitis and treated with injectable corticosteroids	Not fully depictable in routine data
	Share of patients older than 1 year with acute upper respiratory infection prescribed antibacterials for systemic use	Redundant indicator - already depicted in an included indicator
	Share of patients older than 1 year with acute upper respiratory infection prescribed antibacterials for systemic use receiving the recommended antibacterials	Redundant indicator - already depicted in an included indicator
	Share of patients older than 1 year with acute upper respiratory infection prescribed antibacterials for systemic use receiving quinolones	Redundant indicator - already depicted in an included indicator
	Share of patients older than 1 year with acute tonsillitis prescribed antibacterials for systemic use	Indicator is subject to a specific subgroup
	Share of patients older than 1 year with acute tonsillitis prescribed antibacterials for systemic use receiving the recommended antibacterials	Indicator is subject to a specific subgroup

Disease	Indikator	Exclusion reason
Ear nose throat infections	Share of patients older than 1 year with acute tonsillitis prescribed antibacterials for systemic use receiving quinolones	Indicator is subject to a specific subgroup
	Share of patients older than 18 years with acute/chronic sinusitis prescribed antibacterials for systemic use receiving the recommended antibacterials	Redundant indicator - already depicted in an included indicator
	Share of patients older than 18 years with acute/chronic sinusitis prescribed antibacterials for systemic use receiving quinolones	Redundant indicator - already depicted in an included indicator
	Share of patients older than two years with acute otitis media/myringitis prescribed antibacterials for systemic use receiving the recommended antibacterials	Redundant indicator - already depicted in an included indicator
	Share of patients older than two years with acute otitis media/myringitis prescribed antibacterials for systemic use receiving quinolones	Redundant indicator - already depicted in an included indicator
	Share of patients who were prescribed a second antibiotic within five days of the first antibiotic prescription for acute bacterial sinusitis	Not fully depictable in routine data
	Share of patients with streptococcal throat infection treated with penicillin V, amoxicillin, or erythromycin for 10 days or with a single injection of benzathine penicillin	Redundant indicator - already depicted in an included indicator
	Share of patients prescribed with antibiotics for at least 3 weeks after diagnosis of chronic sinusitis	Not fully depictable in routine data
	Share of patients with acute otitis media treated with antibiotics	Redundant indicator - already depicted in an included indicator
	Share of patients with acute tonsillitis/pharyngitistreated with antibiotics	Redundant indicator - already depicted in an included indicator
	Share of patients with acute sinusitis with narrow-spectrum penicillin	Redundant indicator - already depicted in an included indicator
	Share of patients with acute otitis media with narrow-spectrum penicillin	Redundant indicator - already depicted in an included indicator
	Share of patients with acute tonsillitis/pharyngitis with narrow-spectrum penicillin	Redundant indicator - already depicted in an included indicator
	Share of patients with acute sinusitis that receive antibiotics for at least 10 days	Not fully depictable in routine data
	Share of patients with acute sinusitis treated with broad-spectrum penicillin and / or clavulanic acid	Redundant indicator - already depicted in an included indicator
	Share of patients with acute otitis media treated with broad-spectrum penicillin and / or clavulanic acid	Redundant indicator - already depicted in an included indicator
	Share of patients with acute sinusitis treated with macrolides	Redundant indicator - already depicted in an included indicator
	Share of patients with acute tonsillitis/pharyngitis treated with broad-spectrum penicillin and / or clavulanic acid	Redundant indicator - already depicted in an included indicator
	Share of patients with acute otitis media treated with macrolides	Redundant indicator - already depicted in an included indicator
	Share of patients with acute tonsillitis/pharyngitis treated with macrolides	Redundant indicator - already depicted in an included indicator
	Share of patients with acute sinusitis treated with cephalosporins	Redundant indicator - already depicted in an included indicator
	Share of patients with acute otitis media treated with cephalosporins	Redundant indicator - already depicted in an included indicator
	Share of patients with tonsillitis/pharyngitis treated with cephalosporins	Redundant indicator - already depicted in an included indicator
	Share of patients with acute sinusitis treated with quinolones	Redundant indicator - already depicted in an included indicator
	Share of patients with acute otitis media treated with quinolones	Redundant indicator - already depicted in an included indicator

Disease	Indikator	Exclusion reason
Depressive disorders	Share of patients with depression receiving antidepressant pharmacotherapy	Redundant indicator - already depicted in an included indicator
	Share of patients with major depressive episode who received adequate antidepressant therapy	Redundant indicator - already depicted in an included indicator
	Share of patients with depression who are prescribed an anti-anxiety medication as the sole treatment for depression	Redundant indicator - already depicted in an included indicator
	Share of patients with depression with a prescription for benzodiazepines or other medications for 21 days in patients older than 65 years	Redundant indicator - already depicted in an included indicator
	Share of depression patients still on any prescribed antidepressant 180 days after the first prescription date	Redundant indicator - already depicted in an included indicator
	Share of patients with depression with at least one prescription for antidepressants in the observation year.	Not fully depictable in routine data
	Percent of patients with major depression or dysthymia who have been taking antidepressants for at least 6 months.	Redundant indicator - already depicted in an included indicator
	Share of patients 18 years and older diagnosed with a new episode of major depression, treated with an antidepressant, and taking an antidepressant for at least 180 days	Redundant indicator - already depicted in an included indicator
	Share of patients 18 years and older diagnosed with a new episode of major depression, treated with an antidepressant, and taking an antidepressant for at least 84 days	Redundant indicator - already depicted in an included indicator
Diabetes mellitus	Share of patients with diabetes older than 40 years who are prescribed statins	Not in line with medical guidelines
	Share of diabetes patients older than 40 years who were prescribed aspirin or another antithrombotic drug	Not fully depictable in routine data
	Share of diabetes patients 55 years and older who were prescribed angiotensin-converting enzyme (ACE) inhibitors or angiotensin receptor blockers (ARBs)	No evidence for the indicator
	Share of diabetes patients with a dental examination within the last 12 months	Data availability
	Share of diabetes patients who received influenza vaccination	Redundant indicator - already depicted in an included indicator
	Share of diabetes patients with adherence to ACEIs/ARBs	Not fully depictable in routine data
	Share of patients with diabetes and serious mental illness with an eye examination	The indicator strongly limits the reference population
	Share of patients aged 18 years and older with a diagnosis of diabetes mellitus who had a lower extremity neurological exam performed at least once within 12 months	Not fully depictable in routine data
	Share of patients with proven bottleneck syndrome receiving surgical nerve decompression	Not fully depictable in routine data
	Share of patient prevalence of retinopathy and maculopathy within the population known to have diabetes	Not fully depictable in the observation period
	Share of patients with a leg amputation above the ankle within patients with diabetes	Redundant indicator - already depicted in an included indicator
	Share of patients with a stroke within patients with diabetes	Wrong focus of indicator
	Shareof diabetic vulnerable elderly not on anticoagulant/antiplatelet therapy with daily aspirin prescription	Not fully depictable in routine data
	Share of diabetes patients with one or more HbA1c tests annually	Redundant indicator - already depicted in an included indicator
	Share of diabetes patients with use of an oral hypoglycaemic agent and HbA1c level not monitored in the previous six months	Redundant indicator - already depicted in an included indicator
	Share of diabetes patients with a long-acting oral hypoglycaemic agent (glibenclamide or glimepiride) use and HbA1c level not monitored in the previous 6 months	Redundant indicator - already depicted in an included indicator
	Share of diabetes patients with use of insulin and HbA1c level not monitored in the previous 6 months	Redundant indicator - already depicted in an included indicator

Disease	Indikator	Exclusion reason
Diabetes mellitus	Share of diabetes patients with use of insulin or oral hypoglycaemic medicines, use of medicines that may increase or decrease blood glucose concentration and HbA1c level not monitored in the previous 6 months	Redundant indicator - already depicted in an included indicator
	Share of diabetes patients with use of glibenclamide or glimepiride and renal function not monitored in the previous year	Redundant indicator - already depicted in an included indicator
	Share of patients with diabetes and microalbuminuria or proteinuria without bilateral renal artery stenosis receiving ACE- I treatment.	Redundant indicator - already depicted in an included indicator
	Share of diabetic foot patients receiving appropriate treatment (pressure relief, revascularization, antibiotics, resection of necrotic tissue, amputation, etc.)	Not fully depictable in routine data
	Share of diabetic patients with diabetic retinopathy receiving appropriate treatment (conservative treatment, laser coagulation, vitrectomy, cataract extraction)	Redundant indicator - already depicted in an included indicator
	Share of patients 65 years and older with diabetes who received an influenza vaccination	Redundant indicator - already depicted in an included indicator
	Share of diabetes patients under 65 years of age with an influenza vaccination	Redundant indicator - already depicted in an included indicator
	Share of patients with diabetes with a triglyceride measurement within the observation year	No evidence for the indicator
	Share of diabetes patients with hypertension with a A1c measurement within the observation year	Redundant indicator - already depicted in an included indicator
	Share of patients with diabetes mellitus older than 40 years who were prescribed antiplatelet agents in the observation year	Not fully depictable in routine data
	Share of patients aged 18 to 75 years with type 1 or type 2 diabetes who had an eye (retinal) examination	Redundant indicator - already depicted in an included indicator
	Share of patients with a dilated eye examination in last 12 months	Redundant indicator - already depicted in an included indicator
	Share of patients with diabetes who have a hemoglobin A1c testing within the observation year	Redundant indicator - already depicted in an included indicator
	Share of patients with diabetes with a hemoglobin A1c record within the previous 15 months	Redundant indicator - already depicted in an included indicator
	Share of patients with diabetes with a hemoglobin A1c record within the previous 15 months	Redundant indicator - already depicted in an included indicator
	Share of patients with diabetes who have total cholesterol examination in the previous 15 months	Redundant indicator - already depicted in an included indicator
	Share of patients with diabetes mellitus on ACE inhibitor therapy	Redundant indicator - already depicted in an included indicator
	Share of diabetes patients with a major amputation	Not fully depictable in routine data
	Share of diabetes patients with consultation in a diabetology practice	Redundant indicator - already depicted in an included indicator
	Share of diabetes patients enrolled in disease management program for diabetes	Redundant indicator - already depicted in an included indicator
	Share of diabetes patients with laboratory examinations	Redundant indicator - already depicted in an included indicator
Gonarthrosis [arthrosis of knee]	Share of patients with hemarthrosis within 14 days after arthroscopy for primary degenerative changes	Not fully depictable in routine data
	Share of patients with postoperative wound infections within 30 days after arthroscopy for primary degenerative changes	Not fully depictable in routine data
	Share of gonarthrosis patients with a physiotherapy prescription	Data availability
